# Periodic Lorentz gas with small scatterers

**DOI:** 10.1007/s00440-023-01197-6

**Published:** 2023-03-15

**Authors:** Péter Bálint, Henk Bruin, Dalia Terhesiu

**Affiliations:** 1 MTA-BME Stochastics Research Group, Budapest University of Technology and Economics, Műegyetem rkp. 3., Budapest, 1111 Hungary; 2 Department of Stochastics, Institute of Mathematics, Budapest University of Technology and Economics, Műegyetem rkp. 3., Budapest, 1111 Hungary; 3 Faculty of Mathematics, University of Vienna, Oskar Morgensternplatz 1, 1090 Vienna, Austria; 4 Institute of Mathematics, University of Leiden, Niels Bohrweg 1, 2333 CA Leiden, The Netherlands

**Keywords:** Lorentz gas, Small scatterers, Billiards, Limit theorems, Nagaev–Guivarc’h method, Primary: 37D50, Secondary 37A60, 60F05, 60F17, 82C05, 82C40

## Abstract

We prove limit laws for infinite horizon planar periodic Lorentz gases when, as time *n* tends to infinity, the scatterer size $\rho$ may also tend to zero simultaneously at a sufficiently slow pace. In particular we obtain a non-standard Central Limit Theorem as well as a Local Limit Theorem for the displacement function. To the best of our knowledge, these are the first results on an intermediate case between the two well-studied regimes with superdiffusive $\sqrt{n\log n}$ scaling (i) for fixed infinite horizon configurations—letting first $n\rightarrow \infty$ and then $\rho \rightarrow 0$—studied e.g. by Szász and Varjú (J Stat Phys 129(1):59–80, 2007) and (ii) Boltzmann–Grad type situations—letting first $\rho \rightarrow 0$ and then $n\rightarrow \infty$—studied by Marklof and Tóth (Commun Math Phys 347(3):933–981, 2016) .

## Introduction

In this paper we are interested in limit laws for infinite horizon planar periodic Lorentz gases with small scatterers. The Lorentz gas, a popular model of mathematical physics introduced by Lorentz in 1905 [23], is a dynamical system on the infinite billiard table obtained by removing strictly convex scatterers from ${{\mathbb {R}}}^2$. We study the periodic model when these scatterers are round disks of radius $\rho \in (0,1/2)$ positioned at the points of the Euclidean lattice ${{\mathbb {Z}}}^2$. This table can be split up into countably many compact cells, each congruent to the unit square, which can be also regarded as the 2-dimensional flat torus. As usual, a point particle on the table moves with a unit velocity vector along straight lines inside the table, and collides elastically—angle of incidence equals angle of reflection—at the scatterers. This billiard flow produces a billiard map for the Poincaré section of outgoing collisions. The phase space of the billiard map in a single cell is ${{\mathcal {M}}}= \partial O \times [-\frac{\pi }{2}, \frac{\pi }{2}]$, where *O* is a round disk at the origin with radius $\rho$. The phase space representing all cells together is ${\widehat{{{\mathcal {M}}}}} = {{\mathcal {M}}}\times {{\mathbb {Z}}}^2$ and the displacement function $\kappa _\rho :{{\mathcal {M}}}\rightarrow {{\mathbb {Z}}}^2$ indicates the difference in cell numbers going from one collision to the next. As *O* is strictly convex, the billiard is dispersing, and the dynamics has good hyperbolicity properties.

For any $\rho \in (0,1/2)$, the horizon is infinite which means that the time between two consecutive collisions—and accordingly, $\kappa _\rho :{{\mathcal {M}}}\rightarrow {{\mathbb {Z}}}^2$—is unbounded. This corresponds to *corridors*, that is, infinite strips on ${{\mathbb {R}}}^2$ parallel to some direction $\xi \in {{\mathbb {Z}}}^2\setminus \{0\}$ which do not intersect any of the scatterers of the infinite billiard table. Both the number and the geometry of these corridors depend on $\rho$. Hence, the value of the parameter $\rho$ strongly affects the asymptotic properties of the dynamics, and in particular, plays a central role in our exposition.

### Recalling limit laws for fixed $\rho \in (0,1/2)$ as time $n \rightarrow \infty$

A consequence of the infinite horizon is the superdiffusive behaviour of $\kappa _\rho$ with $\rho \in (0,1/2)$ fixed, captured in the first place in the Central Limit Theorem (CLT) with non-standard normalization. To recall this result along with its refinements, we introduce some notation to be used throughout this paper. Let $T_{\rho }:{{\mathcal {M}}}\rightarrow {{\mathcal {M}}}$ be the billiard map, recall that it preserves the canonical invariant probability measure $\mu$. Set

1 $$\begin{aligned} \kappa _{n,\rho }=\sum _{j=0}^{n-1}\kappa _\rho \circ T_\rho ^j, \qquad \Sigma =\frac{1}{\pi } \begin{pmatrix} 1&{}0\\ 0&{}1 \end{pmatrix}. \end{aligned}$$

Choosing the initial point on ${{\mathcal {M}}}$ according to $\mu$, we can regard $\kappa _{n,\rho }$ as a family of random variables. Throughout we let $\implies$ stand for convergence in distribution. We recall the CLT with non-standard normalization: for every $\rho \in (0,\frac{1}{2})$ there exists a positive definite matrix $\Sigma _{\rho }$ such that:

2 $$\begin{aligned} \text {For fixed }\rho \in (0,1/2),\quad \frac{\kappa _{n,\rho }}{\sqrt{n\log n}}\implies \mathcal {N}(0,\Sigma _{\rho }) \text { as } n\rightarrow \infty . \end{aligned}$$

This result was conjectured by Bleher [5] and proved rigorously via two different methods: Szász and Varjú [31], and Chernov and Dolgopyat [11]. In the setting above, the requirement of having two non-parallel corridors (present in [11, 31]) is automatically satisfied because the scatterers are positioned at the lattice points.

It is important to note that there is an explicit formula for $\Sigma _{\rho }$, which involves the scatterer geometry for fixed $\rho$, see for example [11, Formula (2.1)]. A computation (similar to our proof of Lemma A.4) shows that

3 $$\begin{aligned} \lim _{\rho \rightarrow 0} (4\pi \rho ^2) \Sigma _{\rho }=\Sigma , \end{aligned}$$

where $\Sigma$ is the diagonal matrix given in (1). To compare these results with our Theorem A below, we point out the following direct consequence of (2) and (3):

4 $$\begin{aligned} \frac{\kappa _{n,\rho }}{(\sqrt{4\pi }\rho )^{-1}\sqrt{n\log n}} \implies \mathcal {N}(0,\Sigma ) \text { as first } n\rightarrow \infty \text { and then } \rho \rightarrow 0. \end{aligned}$$

The method of proof in [31] relies on the existence of a Young tower for $T_\rho$ as in [9, 34] and an abstract result of Bálint and Gouëzel [4] along with several additional properties of $(\kappa _\rho , T_\rho )$ established in [31]. One notable feature of this method is that it provides a refinement of the CLT (2), namely the Local Limit Theorem (LLT):

5 $$\begin{aligned} \text {For fixed }\rho \in (0,1/2),\quad (n\log n)\mu (\kappa _{n,\rho }=0)\rightarrow \Phi _{\Sigma _{\rho }}(0)\text { as } n\rightarrow \infty , \end{aligned}$$

where $\Phi _{\Sigma _{\rho }}$ is the density of the Gaussian random variable on the r.h.s of (2).

The method of proof in [11] exploits exponential mixing for the sequence $\{\kappa _\rho \circ T_\rho ^n\}_{n\ge 1}$. The authors of that work develop an argument based on standard pairs to establish a bound on the correlations for $\kappa _\rho$:

6 $$\begin{aligned} \begin{array}{l} \text {For fixed }\rho \in (0,1/2),\text { there exist }\hat{\vartheta }_{\rho }\in (0,1) \text { and }\hat{C}_\rho > 0\\ \text {so that }\left| \int _{{{\mathcal {M}}}} \kappa _\rho \cdot \kappa _\rho \circ T_\rho ^n\,d\mu \right| \le \hat{C}_\rho \cdot \hat{\vartheta }_{\rho }^n \text { for all } n\ge 1.\\ \end{array} \end{aligned}$$

Using (6), the CLT (2) is proved in [11, Proof of Theorem 8 a)] via blocking type arguments; we refer to Denker [17] for a classical reference. Furthermore, as shown in [11, Proof of Theorem 8], the limit law (2) together with a tightness argument for a truncated version of $\kappa _\rho$ provides another refinement of the CLT, namely, the Weak Invariance Principle (WIP):

7 $$\begin{aligned} \begin{array}{l} \text {For fixed }\rho \in (0,1/2) \text { and } s\in (0,1), \ \frac{\kappa _{\lfloor ns \rfloor ,\rho }+ \{ns\}(\kappa _{\lfloor ns \rfloor +1,\rho }- \kappa _{\lfloor ns \rfloor ,\rho }) }{\sqrt{n\log n}} \text { converges as }\\ n\rightarrow \infty \text { to a Brownian motion with mean } 0\text { and variance }\Sigma _{\rho }. \end{array} \end{aligned}$$

Similar versions of the CLT (2) and the WIP (7) hold for the flight time function taking values in ${{\mathbb {R}}}^2$, see [11].

In a different direction, a further important consequence of the LLT (5) established in [31] is that it allows one to study mixing of the infinite measure preserving billiard dynamics on the entire lattice ${\widehat{{{\mathcal {M}}}}} = {{\mathcal {M}}}\times {{\mathbb {Z}}}^2$. This can be modelled by a ${{\mathbb {Z}}}^2$ extension

$$\begin{aligned} {\hat{T}}_{\rho }^n(\theta ,\phi ,\ell ) = ( T_{\rho }^n(\theta ,\phi ), \ell +\kappa _{n,\rho }(\theta ,\phi )), \qquad (\theta ,\phi ) \in {{\mathcal {M}}},\ \ell \in {{\mathbb {Z}}}^2. \end{aligned}$$

The dynamics ${\hat{T}}_\rho$ preserves the measure ${\hat{\mu }}=\mu \times \text{ Leb}_{{{\mathbb {Z}}}^2}$, where $\text{ Leb}_{{{\mathbb {Z}}}^2}$ denotes the counting measure. Let us introduce the notation ${{\mathcal {M}}}_{\xi }:={{\mathcal {M}}}\times \{\xi \} \subset {\widehat{{{\mathcal {M}}}}}$ for $\xi \in {{\mathbb {Z}}}^2$, and furthermore, for brevity, let ${{\mathcal {M}}}_0:={{\mathcal {M}}}\times \{(0,0)\}$, where the label 0 refers here to the origin in ${{\mathbb {Z}}}^2$. An immediate consequence of (5) is:

8 $$\begin{aligned}&\text {For fixed }\rho \in (0,1/2),\ (n\log n) \mu (\kappa _{n,\rho }=0) =(n\log n) {\hat{\mu }}({{\mathcal {M}}}_0\cap {\hat{T}}_\rho ^{-n}{{\mathcal {M}}}_0) \nonumber \\&\quad \rightarrow \Phi _{\Sigma _{\rho }}(0) \text { as } n\rightarrow \infty . \end{aligned}$$

A first refinement of the LLT (5) and of the mixing statement (8) was obtained by Pène [28] who proved the analogue of these statements for the class of dynamically Hölder observables. Later on, Pène and Terhesiu [29], building on the results in [4], obtained sharp error rates in the LLT and the mixing for dynamically Hölder observables, including observables supported on compact sets. Furthermore, [29] establish optimal error rates for mean zero observables.

### Recalling results as first $\rho \rightarrow 0$ and then $n \rightarrow \infty$ (Boltzmann–Grad limit)

In [24, 25], Marklof and Strömbergsson studied the Boltzmann–Grad limit of the periodic Lorentz gas. This corresponds to letting the scatterer size $\rho \rightarrow 0$ and investigating the displacement in the rescaled continuous time $T=\rho t$ (so that the mean free path remains bounded). In particular, [24] proves that, in this Boltzmann–Grad limit, the displacement of the particle converges, on any finite time interval, to an explicitly given Markov process. Marklof and Tóth [26] then studied the large time asymptotic of this Markov process, and obtained the CLT and the WIP with non-standard normalization $\sqrt{T\log T}$.

These results on the Boltzmann–Grad limit scenario hold in any dimension, not just in $d=1, 2$ as the results mentioned in the previous subsection. For more details, we refer to the original references. What is most relevant for us is that [26, Theorem 1.1] and [26, Theorem 1.3] are reduced to discrete time statements that can be formulated in terms of the behavior of $\kappa _{n,\rho }$ in the limits $\rho \rightarrow 0$ first and then $n\rightarrow \infty$. In particular, [26, Theorem 1.2] states for $d=2$ that:

9 $$\begin{aligned} \frac{\kappa _{n,\rho }}{(\sqrt{4\pi }\rho )^{-1}\sqrt{n\log n}} \implies \mathcal {N}(0,\Sigma ) \qquad \text { as } \rho \rightarrow 0\text { followed by } n\rightarrow \infty , \end{aligned}$$

where $\kappa _{n,\rho }$ and $\Sigma$ are as in (1),^1^ while [26, Theorem 1.4] is the corresponding WIP which, when $d=2$, reads as (7) with the main difference of the limit paths: $\rho \rightarrow 0$ followed by $n\rightarrow \infty$, as opposed to fixed $\rho$.

In [26], the authors state that *an open problem is to consider the joint limit* $\rho \rightarrow 0$ *and* $n\rightarrow \infty$. In the Boltzmann–Grad limit scenario with *diffusive behaviour*, this type of question is answered by Lutsko and Tóth in [22] for *random Lorentz gases*, in dimension $d=3$, where, on top of the initial condition, additional randomness comes from the *random placement of the scatterers*. However, their model is very different from the model considered in [26] and it is characterized by diffusion (Brownian motion with *standard normalization*).

### Main results as $\rho \rightarrow 0$ and $n \rightarrow \infty$ in the joint limit

Our main result takes a step in answering the open question in [26] for the planar periodic Lorentz gas. It reads as follows.

#### Theorem A

Let $\kappa _{n,\rho }$ and $\Sigma$ be as in (1), and let

$$\begin{aligned} b_{n,\rho }=\frac{\sqrt{n\log (n/\rho ^2)}}{\sqrt{4\pi }\ \rho }. \end{aligned}$$

There exists a function $M(\rho )$ with $M(\rho )\rightarrow \infty$ as $\rho \rightarrow 0$ such that,

$$\begin{aligned} \frac{\kappa _{n,\rho }}{b_{n,\rho }}\implies \mathcal {N}(0,\Sigma ), \text { as } n\rightarrow \infty \text{ and } \rho \rightarrow 0 \text { such that } M(\rho )=o(\log n). \end{aligned}$$

A precise expression of $M(\rho )$ is given in Theorem 7.1 in Sect. 7. At this stage we mention that $M(\rho )$ depends on the rate of correlation decay for Hölder observables as $\rho \rightarrow 0$. How this decay rate depends on $\rho$ is not known and we do not attempt to study this in the present paper. However, we comment on some relevant aspects of correlation decay below.

In the remainder of this section, we make some further comments on how our results compare to various other works, and on some key ingredients of our argument.

*Comments on the rate of correlation decay* Statistical limit laws in dynamical settings in general, and our results in particular, are strongly related to effective bounds on time correlations. For several decades, it has been a major problem to prove exponential decay of correlations for Hölder observables in dispersing billiards, that is, bounds of the form:

10 $$\begin{aligned} \left| \int _{{{\mathcal {M}}}} \psi _1\cdot \psi _2\circ T_\rho ^n \,d\mu \right| \le C_\rho (\psi _1,\psi _2)\cdot \hat{\theta }_{\rho }^n \text { for all } n\ge 0, \end{aligned}$$

where $\psi _1:{{\mathcal {M}}}\rightarrow {{\mathbb {R}}}$ and $\psi _2:{{\mathcal {M}}}\rightarrow {{\mathbb {R}}}$ are centered, Hölder continuous observables, and $\hat{\theta }_{\rho }<1$ may depend on the Hölder exponent, while $C_\rho (\psi _1,\psi _2)>0$ on the Hölder norm of these functions, and both constants depend also on $\rho$ (i.e. on the billiard table). Several powerful methods have been designed to prove bounds of the form (10), in particular using quasi-compactness of the transfer operator on Young towers [34] or anisotropic Banach spaces [14], coupling of standard pairs [10, Chapter 7] or most recently, Birkhoff cones [13]. However, each of these methods involve some non-constructive compactness argument which is the reason why there is no explicit information available on how the rate of decay (i.e. $C_\rho$ and $\hat{\theta }_{\rho }$) depends on $\rho$. For instance, in the framework of quasi-compact transfer operators, this corresponds to having effective bounds on the essential spectral radius, but not on the spectral gap.

In fact, depending on the method, $\psi _1$ and $\psi _2$ may belong to a larger space (that contains Hölder functions), however, these spaces do not contain the unbounded observable $\kappa _{\rho }$. Hence, even for fixed $\rho$, it requires additional effort to obtain correlation bounds for unbounded observables, in particular, to derive (6). As mentioned above, in our context of the infinite horizon Lorentz gas, (6) was proved by Chernov and Dolgopyat in [11, Proposition 9.1], which is an important reference for our work. Let us also mention [3, Lemma 3.2] on a similar bound for the induced return time arising in dispersing billiards with cusps, and the more recent paper [32] where correlation bounds for unbounded observables are studied in an axiomatic framework that includes further billiard models. Nonetheless, all these works consider the large time asymptotics of a fixed billiard system. To treat the simultaneous scaling of $\rho \rightarrow 0$ and $n\rightarrow \infty$, in Appendix C of the present paper we extend [11, Proposition 9.1] in two directions. On the one hand, on top of the mere existence of some $\hat{C}_\rho > 0$ and $\hat{\vartheta }_{\rho }<1$ in (6), we explicitly relate these constants to $C_\rho$ and $\hat{\theta }_{\rho }$ of (10), as expressed in (66).^2^ On the other hand, to exploit correlation bounds of the type (6) when taking the joint limit, these have to be combined with the action of the perturbed transfer operator $R_{\rho }(t)$ (introduced below) as stated in our Proposition C.1.

*Comparison with results on the random Lorentz gas* To compare our Theorem A with the results of Lutsko and Tóth on the random Lorentz gas, it is important to emphasize that although both [22] and our paper consider a joint limit of scatterer radius tending to 0 and time tending to infinity simultaneously, the settings of these two papers are quite different. In particular, the starting point of Lutsko and Tóth is the Boltzmann–Grad limit of the random Lorentz gas, and accordingly, [22] can handle situations when time tends to infinity at a *sufficiently slow* pace in relation to the scatterer size tending to 0. In contrast, the starting point of our work is the superdiffusive limit in the infinite horizon periodic Lorentz gas with fixed scatterer size (see Sect. 1.1 for a summary of previous results), and accordingly we can handle situations when time tends to infinity at a *sufficiently fast* pace in relation to the scatterer size tending to 0.

It is also important to note that under the condition $M(\rho )=o(\log n)$ we have

$$\begin{aligned} \frac{b_{n,\rho }}{(\sqrt{4\pi }\rho )^{-1}\sqrt{n\log n}}\rightarrow 1, \end{aligned}$$

which shows that our Theorem A is indeed a direct analogue of both (4) and (9). To simplify the exposition, we omit the case $d=1$ (i.e., the Lorentz tube), but believe that similar results can be obtained by the same arguments.

*Further comments on some corollaries of our result and some elements of our proofs* A main advantage of the current method of proof via spectral methods is that it allows us to obtain (with no additional effort) the LLT (5) and the mixing statement (8) with appropriate limit paths $\rho \rightarrow 0$ simultaneously with $n\rightarrow \infty$, as opposed to fixed $\rho$. For the LLT we refer to Theorem 7.3 and for the mixing result we refer to Corollary 7.5.

We mention up front that unlike in the *fixed* $\rho$ scenario with main results recalled in Sect. 1.1, we cannot exploit the existence of a Young tower because it seems undoable to build such a tower in a fashion that it depends continuously on $\rho$. Instead, we prove Theorem A via the Nagaev method on Banach spaces of distributions introduced by Demers and Zhang [14–16] in the spirit of the spaces constructed in Demers and Liverani [12]. See Aaronson and Denker [1, 2] for a classical reference on the Nagaev method in (Gibbs Markov) dynamics beyond the CLT with standard normalization (that is $\sqrt{n}$). However, as we shall explain below, the standard pairs argument in [11] plays a crucial role in our proof.

We end this introduction summarizing the main steps and challenges of our proofs. A main difficulty comes from the fact that as $\rho \rightarrow 0$, more and more corridors open up and controlling their number and geometry is a non-trivial task. Another challenge for the proofs of Theorem A and the LLT in Theorem 7.3 comes from the fact that the spaces in [14–16] cannot be used in a straightforward way *even* in the infinite horizon case with *fixed* $\rho$.

The Nagaev method requires: (1) the existence of a Banach space $({{\mathcal {B}}}, \Vert \,\Vert _{{{\mathcal {B}}}})$ on which the transfer operator $R_\rho$ of $T_\rho$ has a spectral gap; (2) that the perturbed transfer operator ($R_\rho (t) \psi =R(e^{it\kappa _\rho }\cdot \psi )$ for $\psi \in {{\mathcal {B}}}$) has sufficiently good continuity estimates $\Vert R_\rho (t)-R_\rho (0)\Vert _{{{\mathcal {B}}}}\le C |t|^\nu$; the larger $\nu > 0$, the better.

Regarding 1), using a Lasota–Yorke inequality on a strong space ${{\mathcal {B}}}$ and a weak space ${{\mathcal {B}}}_w$, Demers and Zhang [14–16] established the spectral gap for every fixed $\rho$, see Sect. 4. This is the main reason why we resorted to the use of such Banach spaces.

Regarding 2), as in Keller and Liverani [20], one could work with the weak space. For infinite horizon billiards, continuity estimates in the strong or weak Banach spaces in [14–16] have not been obtained previously. In Sect. 5.2, we give continuity estimates in such Banach spaces (strong or weak); the estimates there rely heavily on a version of the growth lemma, namely Proposition 3.1. These continuity estimates are $O(|t|^\nu )$ for $\nu <1/2$ with explicit dependence on $\rho$, in both the strong and the weak spaces. This exponent $\nu$ is too small to obtain the asymptotics of the leading eigenvalue of $R_\rho (t)$ directly. Therefore we resort to a decomposition of the eigenvalue in several pieces (see the proof of Proposition 6.3) and exploit the standard pairs arguments in [11] to deal with some parts of the estimate, see Appendix C. Along the way, we give a new proof of the LLT (5) for fixed $\rho$ which is new at an abstract level as well, namely by working on the Banach spaces [14–16] in the absence of good continuity estimates but in the presence of exponential decay of correlations.

The paper is organised as follows: In Sect. 2 we recall some basic properties of hyperbolic billiards and also estimate widths of corridors that open up as $\rho \rightarrow 0$. Section 3 gives the Growth Lemmas, following [14–16] but with estimates made explicit in terms of $\rho$, and including sums over unbounded number of corridors (this is the reason why the estimates are worse than for the usual Growth Lemmas). Section 4 introduces the Banach spaces and recalls the proof of the spectral gap property for the unperturbed transfer operator $R_\rho$, showing that the $\rho$-dependence can be controlled. Section 5 is devoted to the continuity estimates of the perturbed transfer operator $R_\rho (t)$ and Sect. 6 gives the asymptotics of the corresponding leading eigenvalue. The precise statements and proofs of the limit theorems are gathered in Sect. 7.

The appendices give further technical details on corridor sums (Appendix A), distortion (Appendix B) and decay of correlations by a combination of tower and standard pair arguments (Appendix C).

## Preliminaries on Lorentz gas on ${{\mathbb {Z}}}^2$

Our general reference on hyperbolic billiards is Chernov and Markarian [10], the conventions of which are followed in our exposition, except for some minor differences. In particular, we use coordinates $(\theta ,\phi ) \in {{\mathbb {S}}}^1 \times [-\frac{\pi }{2}, \frac{\pi }{2}]$ on ${{\mathcal {M}}}$, where

- $\theta \in {{\mathbb {S}}}^1$ in clockwise orientation describes the collision point on the scatterer (so the corresponding point on $\partial O$ is $(\rho \sin \theta , \rho \cos \theta )$);
- $\phi \in [-\frac{\pi }{2}, \frac{\pi }{2}]$ denotes the outgoing angle that the billiard trajectory makes after a collision at a point with coordinate $\theta$ with the outward normal vector $\textbf{N}_\theta$ at this point (so $\phi = \frac{\pi }{2}$ corresponds to an outgoing trajectory tangent to *O* in the positive $\theta$-direction).

In these coordinates $(\theta ,\phi )$, the measure $\mu$ has the same form $d\mu = \frac{1}{4\pi } \cos \phi \, d\phi \, d\theta$ for all values of the radius $\rho > 0$. Integrals involving the displacement function $\kappa _\rho$, however, do depend on $\rho$. If the flight between $(x,\ell )$ and $( T_\rho (x), \ell + \kappa _\rho (x))$ goes through a corridor for a long time before hitting a scatterer at the boundary of this corridor, then the angle at which the second scatterer is hit is close to $\pm \frac{\pi }{2}$. This sparks another long flight in the same corridor, i.e., $\Vert \kappa _\rho (T_\rho x)\Vert$ is large, too.

In the remainder of this section, we record some properties of $T_\rho$ and $\kappa _\rho$. In Sect. 2.1 the geometry of corridors is described, with special emphasis on the asymptotics of small $\rho$. In Sect. 2.2 we focus on the singularities, which, in addition to strong hyperbolicity, are the other main feature of the map $T_{\rho }:{{\mathcal {M}}}\rightarrow {{\mathcal {M}}}$. In Sect. 2.3, the hyperbolic properties of $T_{\rho }:{{\mathcal {M}}}\rightarrow {{\mathcal {M}}}$ are discussed. Some lemmas of technical character are moved to Appendices A and B.

*Notation:* For functions (or sequences) *f* and *g*, we use the Vinogradov notation $f \ll g$ and the Landau big *O* notation interchangeably: there is a constant $C > 0$ such that $f \le C g$. Similarly $f \asymp g$ means that there exists $C>1$ such that $C^{-1} g \le f \le Cg$.

### Corridors and their widths

Let $O_\ell$ denote the circular scatterer of radius $\rho$ placed at lattice point $\ell \in {{\mathbb {Z}}}^2$. The computation of $\mu (x \in \partial O_0 \times [-\frac{\pi }{2}, \frac{\pi }{2}]: \kappa _\rho (x) = (p,q))$ is based on the division of the phase space in corridors. These are infinite strips in rational directions given by $\xi \in {{\mathbb {Z}}}^2 {\setminus } \{ 0 \}$ for $\rho$ sufficiently small, that are disjoint from all scatterers (but maximal with respect to this property), and they are periodically repeated under integer translations. As soon as $\rho < \frac{1}{2}$, there are infinite corridors parallel to the coordinate axes. If $\rho < \frac{1}{4} \sqrt{2}$, then corridors at angles of $\pm 45^\circ$ open up, and the smaller $\rho$ becomes, the more corridors open up at rational angles.

**Fig. 1:**
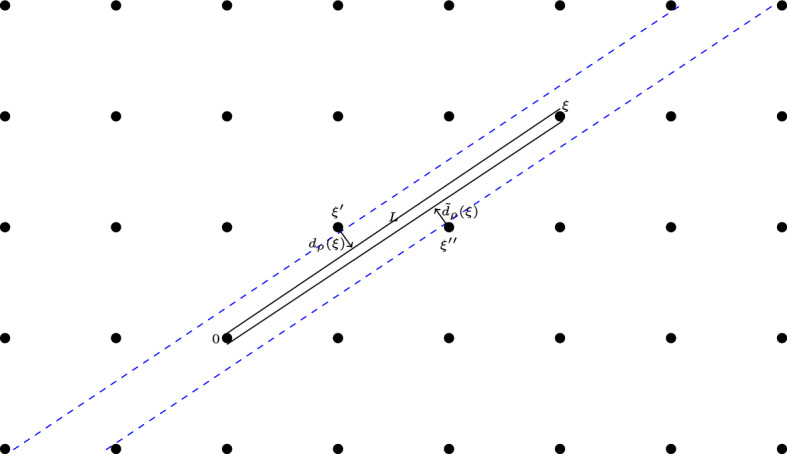
Corridors tangent to the scatterers at 0 and $\xi = (3,2)$

Given $0 \ne \xi \in {{\mathbb {Z}}}^2$ and $\rho > 0$ sufficiently small, there are two corridors simultaneous tangent to $O_0$ and $O_\xi$, one corridor on either side of the arc connecting 0 and $\xi$. The widths of the corridors are denoted by $d_{\rho }(\xi )$ and $\tilde{d}_{\rho }(\xi )$, see Fig. 1.

#### Lemma 2.1

If $\rho = 0$ and $\xi = (p,q) \in {{\mathbb {Z}}}^2$ is expressed in lowest terms, then

$$\begin{aligned} d_0(\xi ) = {\tilde{d}}_0(\xi ) = \frac{1}{|\xi |}. \end{aligned}$$

For $\rho > 0$, the actual width of the corridor is then $d_{\rho }(\xi ) = {\tilde{d}}_{\rho }(\xi ) = \max \{0, |\xi |^{-1} - 2\rho \}$.

#### Remark 2.2

Let us call these two corridors in the direction $\xi$ the $\xi$-corridors. They open up only when $\rho < d_0(\xi )/2 = {\tilde{d}}_0(\xi )/2$. For $\rho = 0$, the common boundary (called $\xi$-boundary) of the two $\xi$-corridors is the line through 0 and $\xi$. The other boundaries are lines parallel to the $\xi$-boundary, going through lattice points that are called $\xi '$ and $\xi ''$ in the below proof. For $\xi = (p,q)$ (with $\gcd (p,q) = 1$), these points $\xi ' = (p',q')$, $\xi '' = (p'',q'')$ are uniquely determined by $\xi$ in the sense that $p'/q'$ and $p''/q''$ are convergents preceding *p*/*q* in the continued fraction expansion of *p*/*q*. In particular $|\xi '|, |\xi ''| \le |\xi |$. In the sequel, we usually only need one of these two $\xi$-corridors, and we take the one with $\xi '$ in its other boundary.

#### Proof

If $(p,q) = (0, \pm 1)$ or $(\pm 1, 0)$, then clearly $d_0(\xi ) = {\tilde{d}}_0(\xi ) = 1$, so we can assume without loss of generality that $p \ge q > 0$. Let *L* be the arc connecting (0, 0) to (*p*, *q*). The corridors associated to $\xi$ intersect $[0,p] \times [0,q]$ in diagonal strips on either side of *L*.

Let $\frac{q}{p} = [0;a_1, \dots , a_n=a]$ be the standard continued fraction expansion with $a \ge 1$, and the previous two convergents are denoted by $q'/p'$ and $q''/p''$, say $q''/p''< q/p <q'/p'$ (the other inequality goes analogously). Therefore $q'p-qp' = 1$ and $q''p'-q'p'' = -1$. Also

$$\begin{aligned} \frac{(a-1)q' + q''}{(a-1)p'+p''}< \frac{q}{p} < \frac{q'}{p'} \end{aligned}$$

are the best rational approximations of *q*/*p*, belonging to lattice points $\xi '$ above *L* and $\xi ''$ below *L*. The vertical distance between $\xi '$ and the arc *L* is $|q'-p'\frac{q}{p}| = \frac{1}{p}|q'p-p'q| = \frac{1}{p}$. The vertical distance between *L* and $\xi ''$ is

$$\begin{aligned} ((a-1)p'+p'')\frac{q}{p} - ((a-1)q'+q'')= & {} \frac{1}{p} ( (a-1)(qp'-q'p) + qp''-q''p )\\= & {} \frac{1}{p} ( 1-a + ( aq'+q'')p'' - (ap'+p'')q'' ) \\= & {} \frac{1}{p} ( 1-a + a(q'p'' - q''p')) = \frac{1}{p}. \end{aligned}$$

The corridor’s diameter is perpendicular to $\xi$, so $d_0(\xi )$ is computed from this vertical distance as the inner product of the vector $(0,1/p)^T$ and the vector $\xi = (p,q)^T$ rotated over $90^\circ$:

$$\begin{aligned} \frac{1}{\sqrt{p^2+q^2}} \left\langle \begin{pmatrix} 0 \\ 1/p \end{pmatrix} \, \ \begin{pmatrix} -q \\ p \end{pmatrix} \right\rangle = \frac{1}{\sqrt{p^2+q^2}} = \frac{1}{|\xi |}. \end{aligned}$$

The computation for ${\tilde{d}}_0(\xi ) = |\xi |^{-1}$ is the same. $\square$

### Singularities of the billiard map

**Fig. 2:**
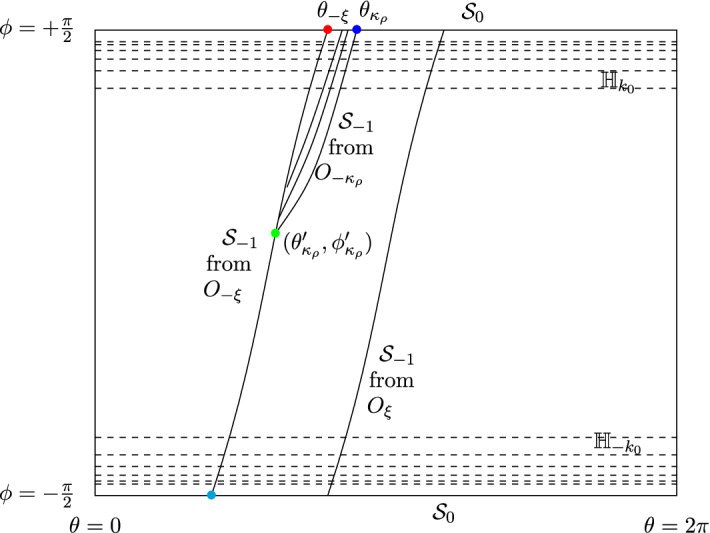
The parameter subset ${{\mathcal {M}}}_0$ with singularity lines and $\kappa _\rho = \xi '-M\xi$

In the coordinates $(\theta ,\phi ,\ell ) \in {{\mathbb {S}}}^1 \times [-\frac{\pi }{2}, \frac{\pi }{2}] \times {{\mathbb {Z}}}^2$ (or $\times {{\mathbb {Z}}}$ if it is a Lorentz tube), the size of the scatterers $\rho$ doesn’t appear, but it comes back in the formula of the billiard map $T_{\rho }$ and in its hyperbolicity. Also the curvature of the scatterers is ${{\mathcal {K}}}\equiv 1/\rho$. We recall some notation from the Chernov and Markarian book [10] (going back to the work of Sinaĭ), bearing in mind that we have to redo several of their estimates to track the precise dependence on $\rho$. The phase space is ${\widehat{{{\mathcal {M}}}}} = {{\mathcal {M}}}\times {{\mathbb {Z}}}^2 = \bigcup _{\ell \in {{\mathbb {Z}}}^2} {{\mathcal {M}}}_\ell$, where each ${{\mathcal {M}}}_\ell$ is a copy of the cylinder ${{\mathbb {S}}}^1 \times [-\frac{\pi }{2}, \frac{\pi }{2}]$, see Fig. 2.

Let ${{\mathcal {S}}}_0 = \{ \phi = \pm \frac{\pi }{2} \}$ be the discontinuity of the billiard map corresponding to grazing collisions. The forward and backward discontinuities are

$$\begin{aligned} {{\mathcal {S}}}_n = \cup _{i=0}^n T_{\rho }^{-i}({{\mathcal {S}}}_0) \quad \text { and } \quad {{\mathcal {S}}}_{-n} = \cup _{i=0}^n T_{\rho }^{i}({{\mathcal {S}}}_0), \end{aligned}$$

so that $T_{\rho }^n:{{\mathcal {M}}}\setminus {{\mathcal {S}}}_n \rightarrow {{\mathcal {M}}}\setminus {{\mathcal {S}}}_{-n}$ is a diffeomorphism. We line the curve ${{\mathcal {S}}}_0$ with homogeneity strips ${{\mathbb {H}}}_k$ bounded by curves $|\pm \frac{\pi }{2}-\phi | = k^{-r_0}$ and $|\pm \frac{\pi }{2}-\phi | = (k+1)^{-r_0}$, $k \ge k_0$, for a fixed number $r_0 > 1$. The standard value is $r_0 = 2$, but as distortion results and some other estimates improve when $r_0$ is larger, we choose the optimal value of $r_0$ later.

**Fig. 3:**
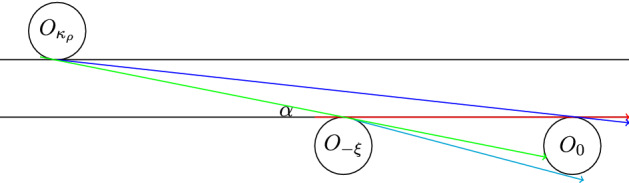
A corridor collision map from $O_{-\xi }$ and $O_{-\kappa _\rho }$ to $O_0$

The set ${{\mathcal {S}}}_{-1}$ consists of multiple curves inside ${{\mathcal {M}}}_0$, one for each scatterer from which a particle can reach $O_0$ in the next collision. In Fig. 2 we consider the corridor in the direction of $\xi \in {{\mathbb {Z}}}^2$, and drew the parts of ${{\mathcal {S}}}_{-1}$ coming from scatterers $O_\xi$, $O_{-\xi }$ and $O_{-\kappa _\rho }$ for some scatterer on the other side of this corridor.

#### Lemma 2.3

For the $\xi$-corridor, let $(\theta _{-\xi }, \frac{\pi }{2}) \in {{\mathcal {M}}}_0$ be the point of intersection of ${{\mathcal {S}}}_0$ and the part of ${{\mathcal {S}}}_{-1}$ associated to the scatterer $O_{-\xi }$, and $(\theta _{\kappa _\rho }, \frac{\pi }{2}) \in {{\mathcal {M}}}_0$, $\kappa _\rho = \xi '-M\xi$, be the point of intersection of ${{\mathcal {S}}}_0$ and the part of ${{\mathcal {S}}}_{-1}$ associated to the scatterer $O_{\kappa _\rho } = O_{\xi '-M\xi }$ at the other side (i.e., the $\xi '$-boundary) of the $\xi$-corridor, see Fig. 3. Let $(\theta '_{\kappa _\rho }, \phi '_{\kappa _\rho })$ be the intersection of the parts of ${{\mathcal {S}}}_{-1}$ associated to the scatterers $O_{-\xi }$ and the scatterer $O_{\kappa _\rho }$, see Fig. 2. Then

$$\begin{aligned} |\theta _{-\xi } - \theta _{\kappa _\rho }| = \frac{d_\rho (\xi )}{|\xi | M} \left( 1+{{\mathcal {O}}}\left( \frac{\rho }{|\xi | M} \right) \right) \end{aligned}$$

and

$$\begin{aligned} \frac{\pi }{2}-\phi '_{\kappa _\rho } = \sqrt{ \frac{2 d_{\rho }(\xi )}{\rho M} } \left( 1-{{\mathcal {O}}}\left( \frac{\rho }{|\xi |} - \frac{1}{M} + \frac{\sqrt{d_{\rho }(\xi ) \rho }}{|\xi | \sqrt{M} }\right) \right) . \end{aligned}$$

#### Proof

The angle $\theta _{-\xi }$ refers to the point where the common tangent line of $O_0$ and $O_{-\xi }$ touches $O_0$. For the value $\theta _{\kappa _\rho }$, $\kappa _\rho = \xi '-M\xi$, we take the common tangent line to $O_0$ and $O_{\kappa _\rho }$ which has slope $\frac{d_{\rho }(\xi )}{M |\xi |} \left( 1+{{\mathcal {O}}}(\frac{\rho }{|\xi | M} ) \right)$. This is then also $|\theta _{-\xi }-\theta _{\kappa _\rho }|$.

**Fig. 4:**
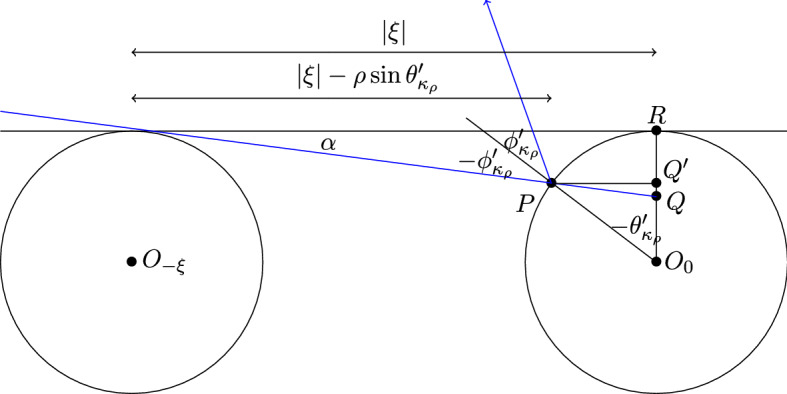
Illustration of the proof of Lemma 2.3

Now for the other endpoint of this piece of ${{\mathcal {S}}}_{-1}$, consider the common tangent line to $O_{-\xi }$ and $O_{\kappa _\rho }$ which has slope $\tan \alpha := \frac{d_{\rho }(\xi )}{(M-1) |\xi |} (1+{{\mathcal {O}}}(\frac{\rho }{|\xi |(M-1)}))$, hitting the scatterer $O_0$ in point *P* and when extended inside $O_0$ hits the vertical line through the center $O_0$ in point *Q*. Let also *R* be the tangent point of $O_0$ to the corridor, and $Q'$ is the point on $O_0R$ at the same horizontal height as *P*, see Fig. 4. Then $|RQ| = |\xi |\sin \alpha$ whereas $|O_0Q'| = \rho - (|\xi | - \rho \sin \theta '_{\kappa _\rho }) \sin \alpha = \rho \cos \theta '_{\kappa _\rho }$. The latter gives

$$\begin{aligned} \theta '_{\kappa _\rho } = \sqrt{ \frac{2|\xi |}{\rho } \sin \alpha \left( 1-{{\mathcal {O}}}(\frac{\rho }{|\xi |} \sin \theta )\right) } = \sqrt{ \frac{2d_\rho (\xi )}{\rho M} \left( 1-{{\mathcal {O}}}(\frac{\rho }{|\xi |} - \frac{1}{M} )\right) }. \end{aligned}$$

The triangle $\triangle PO_0Q$ has angles $\phi '_{\kappa _\rho }$, $\alpha +\frac{\pi }{2}$ and $\theta '_{\kappa _\rho }$, which add up to $\pi$. Hence

11 $$\begin{aligned} \frac{\pi }{2} - \phi '_{\kappa _\rho } = \alpha +\theta '_{\kappa _\rho } = \sqrt{ \frac{2d_\rho (\xi )}{\rho M} } \left( 1-{{\mathcal {O}}}\left( \frac{\rho }{|\xi |} - \frac{1}{M} + \frac{\sqrt{d_{\rho }(\xi ) \rho }}{|\xi | \sqrt{M} }\right) \right) \end{aligned}$$

as claimed. $$\square $$

### Hyperbolicity of the Lorentz gas with small scatterers

The derivative $DT_{\rho }: {{\mathcal {T}}}{{\mathcal {M}}}\rightarrow {{\mathcal {T}}}{{\mathcal {M}}}$ preserves the unstable cone field

12 $$\begin{aligned} {{\mathcal {C}}}^u_x = \left\{ (d\theta , d\phi ) \in {{\mathcal {T}}}_x{{\mathcal {M}}}: 1 \le \frac{1}{2\pi } \frac{d\phi }{d\theta } \le 1 + \frac{\rho }{\tau _{\min }} \right\} . \end{aligned}$$

This is [10, p. 74] in the coordinates $\theta = r/2\pi \rho$, and we can sharpen this cone by replacing $\tau _{\min }$ by $\tau (x)$, the flight time at *x* before the next collision. The derivative of the inverse of the billiard map preserves the stable cone field

13 $$\begin{aligned} {{\mathcal {C}}}^s_x = \{ (d\theta , d\phi ) \in {{\mathcal {T}}}_x{{\mathcal {M}}}: -1-\frac{\rho }{\tau _{\min }} \le \frac{1}{2\pi } \frac{d\phi }{d\theta } \le -1 \}. \end{aligned}$$

Clearly, these cone-fields are transversal uniformly over ${{\mathcal {M}}}$, and ${{\mathcal {S}}}_n$ is a unstable (or stable) curve if $n > 0$ (or $n < 0$).

In the billiard literature it is common to use a pseudo-norm, the *p*-norm for unstable vectors, defined as $\Vert dx \Vert _p = \cos \phi \, dr$. When restricted to the unstable cone, the p-norm is non-degenerate. With the notation ${{\mathcal {R}}}(x) = \frac{2}{\rho \cos \phi }$, the expansion/contraction factor $\Lambda$ on unstable vectors in the p-norm satisfies

$$\begin{aligned} \Lambda \ge 1 + \tau (x) {{\mathcal {R}}}(x) \ge 1 + \tau _{\min } {{\mathcal {R}}}_{\min } = 1 + \frac{2\tau _{\min }}{\rho }. \end{aligned}$$

This proves uniform hyperbolicity of the billiard map.

In our coordinates the *p*-norm can be also expressed as $\Vert dx \Vert _p = 2\pi \, \rho \, \cos \phi \, d\theta$, and it is related to the standard Euclidean norm as

$$\begin{aligned} \Vert dx \Vert = \frac{ \sqrt{1+(\frac{d\phi }{dr})^2} }{\cos \phi } \Vert dx\Vert _p = \frac{ \sqrt{4\pi ^2 \rho ^2 +(\frac{d\phi }{d\theta })^2} }{2\pi \rho \cos \phi } \Vert dx\Vert _p. \end{aligned}$$

The expansion of $DT_{\rho }$ of unstable vectors is uniform in the *p*-norm, see [10, Formula (3.40)]:

$$\begin{aligned} \frac{ \Vert DT_{\rho }(dx)\Vert _p}{\Vert dx\Vert _p} = 1+\frac{\tau (x)}{\cos \phi } ({{\mathcal {K}}}+ \frac{d\phi }{dr}) = \frac{\tau (x)}{\rho \cos \phi } \left( 1+\frac{1}{2\pi } \frac{d\phi }{d\theta } + \frac{\rho \cos \phi }{\tau (x)}\right) . \end{aligned}$$

Expressed in Euclidean norm, this gives, for $DT_{\rho }(dx) = (d\theta _1, d\phi _1)$,

14 $$\begin{aligned} \frac{\Vert DT_{\rho }(dx)\Vert }{\Vert dx \Vert } = \sqrt{\frac{4\pi ^2\rho ^2 + (\frac{d\phi _1}{d\theta _1})^2}{4\pi ^2\rho ^2 + (\frac{d\phi }{d\theta })^2} } \ \frac{\tau (x)}{\rho \cos \phi _1}\ \left( 1+\frac{1}{2\pi } \frac{d\phi }{d\theta } + \frac{\rho \cos \phi }{\tau (x)} \right) . \end{aligned}$$

For later use, if $T_{\rho }(x)$ is in the homogeneity strip ${{\mathbb {H}}}_k$, then $\cos \phi _1 \approx k^{-r_0}$.

## Growth lemmas

As already mentioned in the introduction, the main line of our argument uses perturbed transfer operators acting on the Banach spaces constructed in [14] and [16]. These works, as essentially all other methods studying statistical properties of hyperbolic billiards, rely on appropriately formulated growth lemmas, which quantify the competition of the two main dynamical effects, singularities and expansion, in these systems. The constructions of [14] and [16] involve several exponents, which thus are present in our setting, too. Additionally, we have to introduce some further exponents as we study perturbed transfer operators. Before stating the growth lemmas, here we include a table summarizing the role and the interrelation of these exponents. Essentially, we use the same notation as in [14] except for some subscripts ${}_0$, and in fact some of the constants reduce to their value in [14] if $r_0=2$.

15 $$\begin{aligned} {\left\{ \begin{array}{ll} r_0 \ge 2&{} \text {is the exponent of the homogeneity strips:}\\ &{} \qquad {{\mathbb {H}}}_{\pm k} = \{ |\pm \frac{\pi }{2} - \varphi | \in [(k+1)^{-r_{0}}, k^{-r_{0}})\},\\ 0<\nu<\frac{1}{2}-\frac{1}{2r_{0}} &{} \text {the exponent of } \kappa _\rho \text { in the continuity estimate for the}\\ &{} \text {transfer operator,}\\ \varsigma _{0}=1-\frac{2r_{0}\nu }{r_{0-1}} &{} \text {upper bound on }\varsigma \text { in the Jensenized growth lemma, see } 25, \\ \alpha _{0}< \min \left( \frac{1}{2(r_{0+1})},\varsigma _{0}\right) &{} \text {needed for [14, Lemma 3.7] for general } r_{0},\\ s_0 = \frac{1-\alpha _{0}{(r_{0+1})}}{2r_{0}} > 0 &{} \text {used in Lemma}~6.1,\\ 0< q_{0}< p_{0}< \frac{1}{r_{0+1}} &{} \text {cf. Lemma}~{\textrm{B}}.2,\\ 0< \beta _{0} < \min \{\frac{\alpha _{0}}{2}, p_{0}-q_{0}\}. &{} \end{array}\right. }\nonumber \\ \end{aligned}$$

We use a class ${{\mathcal {W}}}^s$ of *admissible stable leaves* defined as $C^2$ leaves *W* in the phase space such that all its tangent lines are in the stable cone bundle, their second derivative is uniformly bounded, *W* is contained in a single homogeneity strip, $\kappa _\rho (x)$ is constant on *W* and there is a $\rho$-dependent upper bound on |*W*|, namely

16 $$\begin{aligned} \sup _{W \in {{\mathcal {W}}}^s} |W| =\delta _0:= c\rho ^{\nu }, \end{aligned}$$

where the small $c > 0$, to be fixed below, is independent of $\rho$.

Let $W \in {{\mathcal {W}}}^s$ be an admissible stable leaf. The preimage $T_{\rho }^{-1}(W)$ is cut by the discontinuity lines ${{\mathcal {S}}}_1$ and boundaries of homogeneity strips into at most countably many pieces $V_i$. Note that we may have to cut the pieces $V_i$ further into curves $W_i$ of length $\le \delta _0$.

### The growth lemma in terms of $V_i$

The particle can reach the scatterer $O_0$ at the origin from corridors in all directions, indexed by $(\xi , \xi ') \in {\Psi }$, see Fig. 3. If the previous scatterer is $\pm \xi$ itself, we call this a trajectory from the $\xi$-boundary; if the previous scatterer is at lattice point $\xi '-M\xi$, the trajectory comes in from the $\xi '$-boundary, see Remark 2.2. To each such scatterer and homogeneity strip ${{\mathbb {H}}}_k$ belongs at most one $V_i$, and the contraction $|T_{\rho }V_i|/|V_i|$ is governed by (14), where the distortion $T_{\rho }:V_i \rightarrow T_{\rho }V_i$ is uniformly bounded, see Appendix B.

#### Proposition 3.1

Assume $0 \le \nu < \frac{1}{2}-\frac{1}{2r_0}$. Then there is a constant $C > 0$, uniform in $\rho ,\nu$ and $r_0$ such that

$$\begin{aligned} \sum _i |\kappa _\rho (V_i)|^{\nu } \ \frac{|T_{\rho }V_i|}{|V_i|} \le C \left( \rho + \rho ^{-\nu } \, \delta _0 \right) \end{aligned}$$

for every stable leaf $W \in {{\mathcal {W}}}^s$.

#### Remark 3.2

(i) Since $|W| \le \delta _0 \le c \rho ^{\nu }$, there is $\theta _* < 1$ such that

$$\begin{aligned} \sum _{V_i} |\kappa _\rho (V_i)|^{\nu } \ \frac{|T_{\rho }V_i|}{|V_i|} \le 3C (\rho +c) \le \theta _*, \end{aligned}$$

for $\rho$ sufficiently small, and *c* chosen appropriately small. In addition, we assume that

17 $$\begin{aligned} \delta _1 \in (0, \delta _0/2) \ \text { is such that } \ \theta _* e^{C_d \delta _1^{1/(r_0+1)} } =: \theta _1 < 1 \end{aligned}$$

for distortion constant $C_d$ from Lemma B.2;

(ii) As later we will need $\nu > \frac{1}{3}$, we can take $r_0=5$ and $\nu = \frac{3}{8}$.

#### Proof

The homogeneous admissible preimage curves $T_{\rho }^{-1}W=\cup _i V_i$ are obtained by partitioning according to

- incoming corridors $$\xi $$;
- for a fixed corridor $\xi$, the scatterer on which $V_i$ is located. Accordingly, $\kappa _\rho (V_i)=M\xi -\xi '$ for some $M \in {{\mathbb {N}}}$, and the summation is over *M*;
- for a fixed scatterer, the homogeneity strip containing $V_i$, that is, $V_i\subset {{\mathbb {H}}}_k$ for some *k*.

If *W* is on the scatterer $O_{0}$ and $V_i$ is on the scatterer $O_{\xi '-M\xi }$, then both of these scatterers are tangent to the same corridor. The trajectory makes and angle $\sim \frac{d_{\rho }(\xi )}{M |\xi |}$ with the corridor and there is a lower bound on the collision angle given by (11). This puts restrictions on how *M* is related to *k*; as reflected by allowed intersections of homogeneity strips and *M*-cells on Fig. 2. In particular

18 $$\begin{aligned} k \ge C(\rho d_{\rho }(\xi )^{-1} M)^{\frac{1}{2r_0}} \end{aligned}$$

which determines the range of *k* for *M* fixed.

We sum over the homogeneity strips for $\xi$ and *M* fixed on the $\xi '$ boundary.

$$\begin{aligned} \sum _{V_i \in {{\mathcal {M}}}_{\xi '-M\xi }} |\kappa _\rho (V_i)|^{\nu } \ \frac{|T_{\rho }V_i|}{|V_i|}&\ll \frac{\rho |\xi |^{\nu } M^\nu }{|\xi | M} \sum _{k \ge (\max \{ C(\frac{\rho M}{d_{\rho }(\xi )} , 1\} )^{\frac{1}{2r_0} }} \frac{1}{k^{r_0}} \\&\ll \rho ^{\frac{1}{2r_0}+\frac{1}{2}} |\xi |^{\nu -1} d_{\rho }(\xi )^{\frac{1}{2}-\frac{1}{2r_0}} M^{\nu -\frac{3}{2}+\frac{1}{2r_0} } \\&\ll \rho ^{\frac{1}{2r_0}+\frac{1}{2}} |\xi |^{\nu -\frac{3}{2}+\frac{1}{2r_0}} M^{\nu -\frac{3}{2}+\frac{1}{2r_0} }, \end{aligned}$$

where we used that the exponent $\frac{1}{2}-\frac{1}{2r_0}$ of $d_{\rho }(\xi )$ is non-negative. By our assumption that $\nu <\frac{1}{2}-\frac{1}{2r_0}$, this expression is summable over *M*, and therefore the sum over the $\xi '$-boundary of the entire $\xi$-corridor is

$$\begin{aligned} \sum _{\text {corridor }\xi } |\kappa _\rho (V_i)|^{\nu } \ \frac{|T_{\rho }V_i|}{|V_i|}\ll & {} \rho ^{\frac{1}{2}+\frac{1}{2r_0} } |\xi |^{\nu -\frac{3}{2}+\frac{1}{2r_0} }. \end{aligned}$$

The sum over homogeneity strips for $\xi$ fixed on the $\xi$-boundary is no different:

$$\begin{aligned} \sum _{V_i \in {{\mathcal {M}}}_{-\xi }} |\kappa _\rho (V_i)|^{\nu } \ \frac{|T_{\rho }V_i|}{|V_i|}&\ll \frac{\rho |\xi |^{\nu } }{ |\xi | } \sum _{k \ge 1} \frac{1}{k^{r_0}} \ll \rho |\xi |^{\nu -1}. \end{aligned}$$

Next we sum over all opened-up corridors, indexed by all the “visible” lattice points inside a sector of angle $|W|/\sqrt{1+4\pi ^2}$, because only trajectories from scatterers within such a narrow sector can hit $O_0$ at coordinates in *W*. The “visible” corridors will be denoted by ${\Psi }_W$. It can happen that a single corridor, or even a single scatterer in a corridor blocks the entire sector, and we reserve one term for $|\xi | \ge 1$ (which is the worst case because the contraction of $T_{\rho }$ is the weakest). Apart from this corridor, and since we need an upper bound, we can replace we replace |*W*| by a stable curve of length $\delta _0$, and apply Lemma A.6 for $a = 1-\nu$ and $a = \frac{3}{2} - \nu - \frac{1}{2r_0}$. This gives

$$\begin{aligned} \sum _{V_i} |\kappa _\rho (V_i)|^{\nu } \ \frac{|T_{\rho }V_i|}{|V_i|}&\ll \rho + \sum _{(\xi ,\xi ')\in {\Psi }_W} \rho |\xi |^{\nu -1} + \rho ^{\frac{1}{2}+\frac{1}{2r_0}} |\xi |^{\nu -\frac{3}{2}+\frac{1}{2r_0}} \\&\ll \rho + \rho ^{-\nu } \delta _0 + \rho ^{1-\nu } \log (1/\rho ) + \rho ^{1-\nu } \delta _0^{-1} \\&\qquad + \rho ^{-\nu } \delta _0 + \rho ^{1-\nu } \log (1/\rho ) + \rho ^{2-\nu } \delta _0^{-1} \\&\ll \rho + \rho ^{-\nu } \delta _0 + \rho ^{1-\nu } \log (1/\rho ) + \rho ^{1-\nu } \delta _0^{-1}. \end{aligned}$$

Since $\delta _0 = c\rho ^{\nu }$ and $\nu < \frac{1}{2}$, this completes the proof. $\square$

### The growth lemma in terms of $W_i$

The pieces of preimage leaf $V_i \subset T_{\rho }^{-1}(W)$ emerge by natural cutting at the discontinuity set ${{\mathcal {S}}}_1$ and the homogeneity strips, but even so, their lengths can be larger than $\delta _0$, the bound of admissible stable leaves. We therefore need to cut them into shorter pieces, denoted as $W_i$. In the worst case, each $V_i$ needs to be cut into $\delta _0^{-1}$ pieces, which gives the estimate

19 $$\begin{aligned} \sum _i |\kappa _\rho (W_i)|^{\nu } \ \frac{|T_{\rho }W_i|}{|W_i|} \le C \left( \rho \delta _0^{-1} + \rho ^{-\nu } \right) \ll \rho ^{-\nu }. \end{aligned}$$

Although this estimate suffices for some purposes, it is not always good enough for larger iterates $T_{\rho }^n$. The next lemma (which follows [14, Lemma 3.2] or [16, Lemma 3.3]) achieves an estimate, uniform in *n*, for $\nu =0$.

For the next lemma we recall some notation used in [16]. For $W \in {{\mathcal {W}}}^s$, we construct the components ${{\mathcal {G}}}_k(W)$ of $T_{\rho }^{-k}W$ inductively on $k=0,\dots ,n$. That is ${{\mathcal {G}}}_0(W) = \{ W \}$, and to obtain ${{\mathcal {G}}}_{k+1}(W)$ first we apply Proposition 3.1 to each curve in ${{\mathcal {G}}}_k(W)$, and then we partition curves that are longer then $\delta _0$ into pieces of length between $\delta _0$ and $\delta _0/2$. We enumerate the leaves of the *k*-th generation ${{\mathcal {G}}}_k(W)$ as $W_i^k$.

#### Lemma 3.3

There is a constant $C_s>0$, independent of $\rho$, such that

20 $$\begin{aligned} \sum _{W_i^n\in {{\mathcal {G}}}_n(W)} \frac{|T_{\rho }^nW_i^n|}{|W_i^n|}\le C_s, \end{aligned}$$

and

21 $$\begin{aligned} \sum _{W_i^n\in {{\mathcal {G}}}_n(W)} \frac{|W_i^n|^\varsigma }{|W|^\varsigma } \, \frac{|T_{\rho }^nW_i^n|}{|W_i^n|}\le C_s^{1-\varsigma }, \end{aligned}$$

for all $$\varsigma \in [0,1)$$.

#### Proof

Define ${{\mathcal {L}}}_k$ as the collection of indices such that $W_i^k \in {{\mathcal {G}}}_k(W)$ is long, i.e., $|W_i^k| \ge \delta _1$ for $i \in {{\mathcal {L}}}_k$, and ${{\mathcal {I}}}_n(W_j^k)$ as the collection indices of $W_i^n$ such that their most recent long ancestor is $W_j^k \in {{\mathcal {G}}}_k(W)$. If for some $W^n_{i_1}$ no such long ancestor exists, then set $k(i_1)=0$ and $W_{i_1}^n$ belongs to ${{\mathcal {I}}}_n(W)$; if $W^n_{i_2}$ is itself long, then set $k(i_2)=n$. Fix some $j \in {{\mathcal {L}}}_k$. As for $W^i_n\in {{\mathcal {I}}}_n(W_j^k)$ the preimages under $T_{\rho }^{n-k}$ of $T_{\rho }^{n-k}W^i_n$ need not be cut artificially (they are already short), and due to the distortion bound from Lemma B.2,

22 $$\begin{aligned} \sum _{i \in {{\mathcal {I}}}_n(W^k_j)} \frac{|T_{\rho }^{n-k}W_i^n|}{|W_i^n|}\le \theta _1^{n-k}, \quad \text { for } \theta _1 = \theta _*e^{C_d |\delta _1|^{\frac{1}{r_0+1}}}. \end{aligned}$$

Recall that by our assumption $\delta _1$ is so small that $\theta _1 < 1$. In the estimate below, we group $W_i^n\in {{\mathcal {G}}}_n(W)$ according to their most recent long ancestors.

23 $$\begin{aligned} \sum _i \frac{|T_{\rho }^nW_i^n|}{|W^n_i|}= & {} \sum _{k=1}^n \sum _{W_j^k \in {{\mathcal {L}}}_k(W)} \sum _{i \in {{\mathcal {I}}}_n(W_j^k)} \frac{|T_{\rho }^nW_i^n|}{|W^n_i|} + \sum _{i \in {{\mathcal {I}}}_n(W)} \frac{|T_{\rho }^nW_i^n|}{|W^n_i|} \nonumber \\\le & {} \sum _{k=1}^n \sum _{W_j^k \in {{\mathcal {L}}}_k(W)} \left( \sum _{i \in {{\mathcal {I}}}_n(W_j^k)} \frac{|T_{\rho }^{n-k}W_i^n|}{|W^n_i|} \right) e^{\delta _1^{1/r_0+1} C_d} \frac{|T_{\rho }^kW_j^k|}{|W^k_j|} + \theta _1^n \nonumber \\\le & {} \sum _{k=1}^n \sum _{W_j^k \in {{\mathcal {L}}}_k(W)} \theta _1^{n-k} \delta _1^{-1} |T_{\rho }^kW_j^k| +\theta _1^n \nonumber \\\le & {} C\delta _1^{-1}|W| \sum _{k=1}^n \theta _1^{n-k} +\theta _1^n\le C_s, \end{aligned}$$

where we have used that for fixed *k* and ${W_j^k \in {{\mathcal {L}}}_k(W)}$, (i) $|W_j^k|\ge \delta _1$, (ii) the $T_{\rho }^kW_j^k$ are pairwise disjoint subcurves of *W*, and (iii) $|W|\le \delta _1$. By Jensen’s inequality and (23),

$$\begin{aligned} \sum _i \frac{|W_i^n|^\varsigma }{|W|^{\varsigma }} \frac{|T_{\rho }^nW_i^n|}{|W^n_i|}= & {} \sum _i \left( \frac{|W|}{|W_i^n|} \right) ^{1-vs} \frac{|T_{\rho }^nW_i^n|}{|W|} \le \left( \sum _i \frac{|T_{\rho }^nW_i^n|}{|W^n_i|} \right) ^{1-\varsigma } \ll C_s^{1-\varsigma }, \end{aligned}$$

which proves the second statement. $\square$

It is worth including the following bound, which follows from (22) by Jensen inequality:

24 $$\begin{aligned} \sum _{i \in {{\mathcal {I}}}_n(W)} \frac{|W_i^n|^\varsigma }{|W|^{\varsigma }} \frac{|T_{\rho }^n W_i^n|}{|W_i^n|}\le \theta _1^{(1-\varsigma )n}, \qquad \text { for all } \varsigma \in [0,1). \end{aligned}$$

#### Remark 3.4

For further reference, we state a version of (21) for $\nu >0$, $n=1$. Let $\varsigma _0=1-\frac{2r_0\nu }{r_0-1}$.

25 $$\begin{aligned} \sum _i |\kappa _\rho (W_i)|^{\nu } \ \frac{|T_{\rho }W_i|}{|W_i|} \frac{|W_i|^\varsigma }{|W|^{\varsigma }} \ll \rho ^{-\nu }, \qquad \text { for all } \varsigma \in [0,\varsigma _0). \end{aligned}$$

This follows by Jensen’s inequality from (19), applied with $\frac{\nu }{1-\varsigma }$ in place of $\nu$. The condition $\varsigma <\varsigma _0$ ensures that $\frac{\nu }{1-\varsigma }<\frac{1}{2}-\frac{1}{2r_0}$. For the choices $r_0=5$, $\nu =\frac{3}{8}$ we have $\varsigma _0=\frac{1}{16}$.

## Banach spaces and spectral gap

For the exponents $p_0$ and $q_0$ defined in (15) we define the Banach spaces (of distributions) $C^{p_0}, {{\mathcal {B}}}, {{\mathcal {B}}}_w, (C^{q_0})'$ in analogy to [16],^3^ We recall that $(C^{q_0})'$ is the topological dual of $C^{q_0}$.

Given $W\in {{\mathcal {W}}}^s$, let $m_W$ be the Lebesgue measure on *W*, and define

$$\begin{aligned} |\psi |_{W,\alpha ,p_0}:= |W|^{\alpha } \cos W\, |\psi |_{C^{p_0}}, \qquad |\psi |_{C^{p_0}}:= |\psi |_{C^0} + H^{p_0}_W(\psi ),s \end{aligned}$$

for $\alpha \ge 0$, $\cos W = |W|^{-1} \int _W \cos \phi \, dm_W$ (note that $\cos W \ll k^{-r_0}$ if $W \subset {{\mathbb {H}}}_{\pm k}$), and $H^{p_0}_W(\psi )$ the Hölder constant of $\psi$ along *W*. Also let $d_W(W_1, W_2)$ stand for the distance between leaves as in [14, Sect. 3.1] or [16, Sect. 3.1]; in particular, if $W_1$ and $W_2$ belong to the same homogeneity strip, $d_W(W_1, W_2)$ is the $C^1$ distance of their graphs in the $(\theta ,\phi )$ coordinates, and otherwise infinite.

Given $W\in {{\mathcal {W}}}^s$ and $h\in C^1(W)$, define the *weak norm*.^4^

26 $$\begin{aligned} \Vert h\Vert _{{{\mathcal {B}}}_w}:=\sup _{W\in {{\mathcal {W}}}^s}\;\sup _{{\mathop { |\psi |_{W,0,p_0}\le 1}\limits ^{|\psi | \in C^{p_0}(W) }} } \int _W h\psi \, dm_W. \end{aligned}$$

With $q_0 < p_0$ fixed we define the distance between functions $d(\psi _1,\psi _2)$ in the same way as in [14, Sect. 3.1]. We define the *strong stable norm* by

27 $$\begin{aligned} \Vert h\Vert _s:=\sup _{W\in {{\mathcal {W}}}^s}\;\sup _{{\mathop {|\psi |_{W,\alpha _0,q_0}\le 1}\limits ^{\psi \in C^{q_0}(W)}}} \int _W h\psi \, dm_W. \end{aligned}$$

Choosing $\varepsilon _0 \in (0,\delta _0)$ and $\beta _0 \in (0, \min \{ \alpha _0, p_0-q_0\})$, we define the *strong unstable norm* by

28 $$\begin{aligned} \Vert h\Vert _u:=\sup _{\varepsilon \le \varepsilon _0} \sup _{{\mathop { d(W_1, W_2)\le \varepsilon }\limits ^{W_1, W_2\in {{\mathcal {W}}}^s}}} \sup _{\begin{array}{c} {1}{\psi _i \in C^{p_0}(W),\ }\\ { |\psi _i|_{C^1(W)} \le 1 } \\ { d_{q_0}(\psi _1,\psi _2) \le \varepsilon } \end{array}} \frac{1}{\varepsilon ^{\beta _0}}\left| \int _{W_1} h\psi _1\, dm_{W} - \int _{W_2} h\psi _2\, dm_{W}\right| . \end{aligned}$$

The *strong norm* is defined by $\Vert h\Vert _{{{\mathcal {B}}}}=\Vert h\Vert _s+c_u\Vert h\Vert _u$, where we will fix $c_u\ll 1$ (but independent of $\rho$) at the beginning of Sect. 5.2.

Since $C^{p_0}\subset {{\mathcal {B}}}\subset {{\mathcal {B}}}_w\subset (C^{q_0})'$ (see Sect. 4.1), we have $\Vert h\Vert _{{{\mathcal {B}}}_w}+\Vert h\Vert _{{{\mathcal {B}}}}\le C\Vert h\Vert _{C^1}$. As in [16], we define ${{\mathcal {B}}}$ to be the completion of $C^1$ in the strong norm and ${{\mathcal {B}}}_w$ to be the completion in the weak norm.

### Transfer operator on ${{\mathcal {B}}}$

Throughout we let $R_\rho : L^1(m)\rightarrow L^1(m)$ be the transfer operator of the billiard map $T_\rho$. We recall that [14, Lemmas 3.7$-$3.10] ensure that: i) $R_\rho (C^1)\subset {{\mathcal {B}}}$ and as a consequence *R* is well defined on ${{\mathcal {B}}}$; ${{\mathcal {B}}}_w$; ii) the unit ball of ${{\mathcal {B}}}$ is compactly embedded in ${{\mathcal {B}}}_w$, and iii) $C^{p_0}\subset {{\mathcal {B}}}\subset {{\mathcal {B}}}_w\subset (C^{q_0})'$.

It follows that $R_\rho$ is well defined on ${{\mathcal {B}}}$ and ${{\mathcal {B}}}_w$, and we also let $R_\rho$ denote the extension of this transfer operator to ${{\mathcal {B}}}_w$.

### Lasota–Yorke inequalities

Using Proposition 3.1 with $\nu =0$ and Lemma 3.3 we obtain the analogue of the Lasota–Yorke inequality [16, Proposition 2.3]. As our set-up fits [16], our only concern is the dependence on $\rho$. It is important to point out that our all estimates in Sect. 3 and Appendix B are independent of $\rho$, except that $\delta _1<\delta _0\ll \rho ^{\nu }$.

#### Lemma 4.1

(Weak norm) There exists a uniform constant $C>0$ so that for all $h\in {{\mathcal {B}}}$ and for all $n\ge 0$,

$$\begin{aligned} \Vert R_\rho ^n h\Vert _{{{\mathcal {B}}}_w}\le C\cdot C_s\,\Vert h\Vert _{{{\mathcal {B}}}_w}, \end{aligned}$$

where $C_s$ is given by (20).

#### Proof

For $$W\in {{\mathcal {W}}}^s$$, $$h\in C^1({{\mathcal {M}}}_0)$$, $$\psi \in C^{p_0}(W)$$ with $$|\psi |_{W,\alpha _0,p_0}\le 1$$,

$$\begin{aligned} \int _W R_\rho ^n h \psi \, dm_W=\sum _{W_i^n\in {{\mathcal {G}}}_n(W)}\int _{W_i^n} h \frac{J_{W_i^n} T_\rho ^n}{|DT_{\rho }^n|}\psi \circ T_\rho ^n\, dm_W. \end{aligned}$$

Using the present definition of the weak norm,

$$\begin{aligned} \int _W R_\rho ^n h \psi \, dm_W\le \sum _{W_i^n\in {{\mathcal {G}}}_n(W)}\int _{W_i^n} \Vert h\Vert _{{{\mathcal {B}}}_w} \frac{|J_{W_i} T_\rho |_{C^{p_0}(W_i)}}{|DT_\rho |}|\psi \circ T_\rho |_{C^{p_0}(W_i)}\cos (W_i^n)\, dm_W. \end{aligned}$$

From here on the argument goes almost word for word as the argument in [16, Sect. 4.1], except for the use of equation (20) (the analogue of [16, Lemma 3.3(a)] with $\varsigma =0$). $\square$

#### Lemma 4.2

(Strong stable norm) Take $\delta _1$ as in (17) and $\theta _1$ as in (22). There exists a uniform constant $C>0$ so that for all $h\in {{\mathcal {B}}}$ and all $n\ge 0$,

$$\begin{aligned} \Vert R_\rho ^n h\Vert _{s}\le C \left( \theta _1^{(1-\alpha _0)n}+C_s^{1-\alpha _0}\Lambda ^{-q_0n}\right) \Vert h\Vert _{s}+ C\delta _1^{-\alpha _0}\Vert h\Vert _{{{\mathcal {B}}}_w}. \end{aligned}$$

#### Remark 4.3

The compact term $C\delta _1^{-\alpha _0}\Vert h\Vert _{{{\mathcal {B}}}_w}$ in Lemma 4.2 is the only point in the Lasota–Yorke inequalities where a $\rho$-dependence arises, via $\delta _1 = c \rho ^{\nu }$.

#### Proof

The argument goes almost word for word as the [16, Argument in Sect. 4.2], except for the differences:

i) We use of equation (21) with $\varsigma =\alpha _0$ instead of [16, Lemma 3.3 (b)] (also with $\varsigma =\alpha _0$) in [16, Equation (4.5)]. In particular, using the present definition of the stable norm, with the same notation as in [16, Sect. 4.2], we have the following analogue of [16, Equation (4.5)]:

$$\begin{aligned} \sum _{W_i^n\in {{\mathcal {G}}}_n(W)}&\int _{W_i^n} h \frac{J_{W_i^n} T_\rho ^n}{|DT_\rho ^n|}\left( \psi \circ T_\rho ^n-{\bar{\psi }}_i\right) \, dm_W\\&\ll \Lambda ^{-q_0n} \Vert h\Vert _{s}\sum _{W_i^n\in {{\mathcal {G}}}_n(W)} \frac{|W_i^n|^{\alpha _0}}{|W|^{\alpha _0}} \, \frac{|T_\rho ^nW_i^n|}{|W_i^n|} \, \ll \Lambda ^{-q_0n} \Vert h\Vert _{s}, \end{aligned}$$

where we have used the distortion bounds of Appendix B and Formula (21) (with $\varsigma =\alpha _0$).

ii) To obtain the analogue of [16, Equation (4.6)], as in [16, Sect. 4.2], we split the sum

$$\begin{aligned} \sum _{k=0}^n\sum _ {j\in L_k}\sum _{i\in {{\mathcal {I}}}_n(W_j^k)} |W|^{-\alpha _0} (\cos W)^{-1} \int _{W_i^n} h \frac{J_{W_i^n}T_{\rho }^n}{|DT_{\rho }^n|}\, dm_W \end{aligned}$$

into a term for $k=0$ and further terms for $k=1,\dots , n$. For $k=0$, we use the strong stable norm and (24) (the analogue of [16, Lemma 3.3(a)]) with $\varsigma =\alpha _0$, giving a contribution $\ll \Vert h\Vert _{s} \theta _1^{n(1-\alpha _0)}$. For the terms $k=1,\dots n$, we use the weak norm, (21) (the analogue of [16, Lemma 3.3(b)]) with $\varsigma =\alpha _0$, and the fact that $|W^k_j|\ge \delta _1$ for $j\in {{\mathcal {L}}}_k(W)$, resulting in a contribution of $O(\Vert h\Vert _{{{\mathcal {B}}}_w} \delta _1^{-\alpha _0})$. $\square$

As in [16], dealing with the strong unstable norm is the most delicate part of the Lasota–Yorke inequality. The only difference from [16, Argument in Sect. 4.3] is that we apply (20) (instead of [16, Lemma 3.3 (b)]) multiple times. Note that our bound in (20) is independent of $\rho$, so no $\rho$-dependence arises here.

#### Lemma 4.4

(Strong unstable norm) There exists a uniform constant $C>0$ so that for all $h\in {{\mathcal {B}}}$ and for all $n\ge 0$,

$$\begin{aligned} \Vert R_\rho ^n h\Vert _{u}\le C\cdot C_s \cdot \Lambda ^{-\beta _0 n}\Vert h\Vert _{u}+ C\cdot C_s \cdot n\Vert h\Vert _{s}. \end{aligned}$$

#### Proof

Given $W_1,W_2 \in {{\mathcal {W}}}^s$ with $d(W_1,W_2)\le \varepsilon$, we may identify matched and unmatched pieces in $T_\rho ^{-n}W_{\ell }$, $\ell =1,2$. The estimates of [16] on the length of the *unmatched pieces* apply, thus we may estimate their contribution by the strong stable norm using (20) (instead of [16, Lemma 3.3 (b)]). As the length estimates give $\varepsilon ^{\alpha _0/2}$, $\beta _0<\alpha _0/2$ is essential here (cf. [16, Formulas (4.10) and (4.11)], noting that $\gamma =0$ in our case).

To bound the contribution of the *matched pieces* we use, on the one hand, the strong unstable norm (as in [16, Formula (4.14)]) and, on the other hand, the strong stable norm (as in [16, Formula (4.17)]). Here again we rely on equation (20) which plays the role of [16, Lemma 3.3 (b)]. $\beta _0<p_0-q_0$ ensures that after division by $\varepsilon ^{\beta _0}$ the proof of Lemma 4.4 can be completed. $\square$

## Perturbed transfer operators

A standard way of obtaining limit theorems for dynamical systems is via the perturbed transfer operator method. In Sect. 7 we will use the spectral properties of the family of perturbed transfer operators ${\hat{R}}_\rho (t), t\in {{\mathbb {R}}}$ with ${\hat{R}}_\rho (t) h =R(e^{it\kappa _\rho } h)$, $h\in L^1(m)$.

### Continuity properties

By definition, ${\hat{R}}_\rho (0)=R_\rho$. Take $0 \le \nu < \frac{1}{2} - \frac{1}{2r_0}$ as in Proposition 3.1. In this subsection we show the following continuity estimate:

29 $$\begin{aligned} \Vert ({\hat{R}}_\rho (t)-{\hat{R}}_\rho (0))h\Vert _{{{\mathcal {B}}}} \le C \rho ^{-\nu } |t|^\nu \Vert h\Vert _{{{\mathcal {B}}}} \end{aligned}$$

for some uniform constant *C*.

The argument goes parallel to Sect. 4.2, except that this time we need the estimates (i) for $\nu >0$ and (ii) only for $n=1$, we rely on (19) and (25) instead of Lemma 3.3.

#### Lemma 5.1

Assume (16). Then there exists a uniform constant $C>0$ so that for all $h\in {{\mathcal {B}}}$,

$$\begin{aligned} \Vert R_\rho (e^{it\kappa _\rho }-1) h)\Vert _{{{\mathcal {B}}}_w} \le C \rho ^{-\nu } |t|^{\nu } \Vert h\Vert _{{{\mathcal {B}}}_w}. \end{aligned}$$

#### Proof

The argument goes similarly to the argument in [16, Sect. 4.1] restricted to the case $n=1$. More precisely, for $W\in {{\mathcal {W}}}^s$, $h\in C^1({{\mathcal {M}}}_0)$, $\psi \in C^{p_0}(W)$ with $|\psi |_{W,\alpha _0,p_0}\le 1$,

$$\begin{aligned} \int _W R_\rho (e^{it\kappa _\rho }-1) h \psi \, dm_W=\sum _{i\in {{\mathcal {G}}}_1(W)}\int _{W_i} (e^{it\kappa _\rho }-1) h \frac{J_{W_i} T_\rho }{|DT_{\rho }|}\psi \circ T_\rho \, dm_W. \end{aligned}$$

Using the definition of the weak norm and the inequality $|e^{ix}-1|\le x^\nu$,

$$\begin{aligned} \int _W R_\rho (e^{it\kappa _\rho }-1) h \psi \, dm_W&\le \ |t|^{\nu }\sum _{i\in {{\mathcal {G}}}_1(W)}\int _{W_i}\Vert h\Vert _{{{\mathcal {B}}}_w} |\kappa _\rho (W_i)|^\nu \\&\quad \times \frac{|J_{W_i} T_\rho |_{C^{p_0}(W_i)}}{|DT_\rho |}|\psi \circ T_\rho |_{C^{p_0}(W_i)}\cos (W_i)\, dm_W. \end{aligned}$$

From here on the proof goes the same as the argument in [16, Sect. 4.1] except for the use of equation (19) instead of [16, Lemma 3.3 (b)]. $\square$

#### Lemma 5.2

There exists a uniform constant $C>0$ so that for all $h\in {{\mathcal {B}}}$ and for all $n\ge 0$,

$$\begin{aligned} \Vert R_\rho (e^{it\kappa _\rho }-1) h)\Vert _{s}\le C|t|^{\nu } \rho ^{-\nu } \Vert h\Vert _{s}. \end{aligned}$$

#### Proof

This time we are only concerned with $n=1$, and do not need a contraction of the strong stable norm. Hence, an argument analogous to the proof of Lemma 5.1 suffices, with the weak norm replaced by the strong stable norm. Accordingly, we use (25) with $\varsigma =\alpha _0$ instead of [16, Lemma 3.3 (b)]. $\square$

#### Lemma 5.3

There exists a uniform constant $C>0$ so that for all $h\in {{\mathcal {B}}}$,

$$\begin{aligned} \Vert R_\rho (e^{it\kappa _\rho }-1) h)\Vert _{u}\le C|t|^{\nu }\left( \rho ^{-\nu } \cdot \Vert h\Vert _{u}+\rho ^{-\nu } \cdot \Vert h\Vert _{s}\right) . \end{aligned}$$

#### Proof

As with the proof of Lemma 4.4, the argument goes similar to [16, Argument in Sect. 4.3], restricted to the case $n=1$. The matched and unmatched pieces can be again identified, this time for $T_{\rho }^{-1}W_{\ell }$, $\ell =1,2$. Then, as in the proof of Lemma 5.1, the factors $|t|^{\nu }$ and $|\kappa _\rho |^{\nu }$ arise. Clearly $\kappa _\rho$ is constant on each of the (matched or unmatched) pieces, and takes the same value on any two pieces that are matched. Accordingly, the various contributions can be estimated in the same way as in proof of Lemma 4.4, with the only difference that, by the presence of the factor $|\kappa _\rho |^{\nu }$, throughout the argument (19) is used instead of (20). $\square$

Equation (29) follows from the definition of the norm in ${{\mathcal {B}}}$ together with Lemmas 5.1, 5.2 and 5.3.

### Peripheral spectrum and spectral gap

Choose $1>\sigma >\max \{\Lambda ^{-\beta _0}, \theta _1^{(1-\alpha _0)}, \Lambda ^{-q_0}\}$. By Lemmas 4.1, 4.2 and 4.4 and arguing as in [16, Eq. (2.14)], we obtain the traditional Lasota–Yorke inequality for some $N\ge 1$, provided $c_u$ in the definition of $\Vert \ \Vert _{{{\mathcal {B}}}}$ (below (28)) is chosen small enough in terms of *N*. That is,

30 $$\begin{aligned} \Vert R_\rho ^N h\Vert _{{{\mathcal {B}}}}\le \sigma ^N\Vert h\Vert _{{{\mathcal {B}}}}+C \delta _1^{-\alpha _0} \Vert h\Vert _{{{\mathcal {B}}}_w}. \end{aligned}$$

Combined with the properties collected in Sect. 4.1 (that is, the relative compactness of the unit ball of ${{\mathcal {B}}}$ in ${{\mathcal {B}}}_w$), equation (30) shows that the essential spectral radius of $R_\rho$ is bounded by $\sigma$ and that the spectral radius is 1.

Let $\Pi _\rho$ be the eigenprojection (that is, the projection on the eigenspace of $R_\rho$) corresponding to the eigenvalue 1. In particular, $\Pi _{\rho } \mu =\mu$ is the invariant measure for $T_{\rho }$. Since for every $\rho$, $T_\rho$ is mixing, the peripheral spectrum of $R_\rho$ consists of just the simple eigenvalue at 1. Thus, for every $\rho >0$, the eigenprojection $\Pi _{\rho }$ corresponding to the eigenvalue 1 of $R_{\rho }$ can be also characterized by

31 $$\begin{aligned} \Pi _{\rho }h=\lim _{m\rightarrow \infty }R_{\rho }^m h, \end{aligned}$$

for all $$h\in {{\mathcal {B}}}$$.

Let $Q_{\rho }$ be complementary spectral projection. From here onwards, we exploit that for every $\rho >0$, there exist $\gamma _{\rho }\in (0,1)$ and $C_\rho >0$ so that

32 $$\begin{aligned} \Vert Q_{\rho }^m\Vert _{{{\mathcal {B}}}}\le C_\rho (1-\gamma _{\rho })^m \end{aligned}$$

for every $m\ge 1$. Altogether, $R_{\rho }^m=\Pi _{\rho } +Q_{\rho }^m$, where $Q_{\rho }$ satisfies (32).

## Asymptotics of the dominant eigenvalue

To establish limit theorems (such as Theorem 7.1 below) we study the asymptotics of ${{\mathbb {E}}}_\mu (e^{it\kappa _{m,\rho } } 1)={{\mathbb {E}}}_\mu ({\hat{R}}_{\rho }(t)^m 1),$ as $t\rightarrow 0$ and $m\rightarrow \infty$ via the properties of ${\hat{R}}_{\rho }(t) h= R_{\rho }(e^{it\kappa _\rho } h)$, $h\in {{\mathcal {B}}}$.

We already know that for every $\rho \in (\frac{1}{3},\frac{1}{2})$, 1 is a simple eigenvalue of ${\hat{R}}_{\rho }(0)=R_{\rho }$ when viewed as an operator from ${{\mathcal {B}}}$ to ${{\mathcal {B}}}$. Due to (29), ${\hat{R}}_{\rho }(t)$ is $C^\nu$ (in *t*) from ${{\mathcal {B}}}$ to ${{\mathcal {B}}}$. It follows that for *t* in a neighbourhood of 0, ${\hat{R}}_{\rho }(t)$ has a dominant eigenvalue $\lambda _{\rho }(t)$ (with $\lambda _{\rho }(0)=1$).

Let $\gamma _{\rho }$ be as in equation (32). The continuity properties together with (32) ensure that for any $\delta \in (0,\gamma _{\rho })$ and $t\in B_\delta (0)$,

33 $$\begin{aligned} {\hat{R}}_{\rho }(t)^m=\lambda _{\rho }(t)^m\Pi _{\rho }(t)+Q_{\rho }(t)^m,\quad \Vert Q_{\rho }(t)^m\Vert _{{{\mathcal {B}}}}\le C_\rho (1-\gamma _{\rho })^m, \end{aligned}$$

for some $C_\rho >0$ and $\Pi _{\rho }(t)^2=\Pi _{\rho }(t)$, $\Pi _{\rho }(t)Q_{\rho }(t)=Q_{\rho }(t)\Pi _{\rho }(t)=0$. Further, for all $t\in B_\delta (0)$,

34 $$\begin{aligned} \Pi _{\rho }(t)=\int _{|u-1|=\delta }(u-{\hat{R}}_{\rho }(t))^{-1}\, du, \end{aligned}$$

for all *t* small enough. A standard consequence of (29) and (32) is that for every $\delta \in (0,\gamma _{\rho })$ and for all *u* so that $|u-1|=\delta$,

35 $$\begin{aligned} \Vert (u-{\hat{R}}_{\rho }(t))^{-1}-(u-{\hat{R}}_{\rho }(0))^{-1}\Vert _{{{\mathcal {B}}}}&\le C \rho ^{-\nu } |t|^{\nu }\Vert (u-{\hat{R}}_{\rho }(t))^{-1}\Vert _{{{\mathcal {B}}}}\Vert (u-{\hat{R}}_{\rho }(0))^{-1}\Vert _{{{\mathcal {B}}}} \nonumber \\&\le C \rho ^{-\nu }\gamma _{\rho }^{-2}|t|^{\nu }. \end{aligned}$$

Hence, $$\Vert \Pi _{\rho }(t)-\Pi _{\rho }(0)\Vert _{{{\mathcal {B}}}}\le C\rho ^{-\nu } |t|^{\nu }\rho ^{-\nu }\gamma _{\rho }^{-2}|t|^{\nu }$$.

The rest of this section is allocated to the study the asymptotics of $\lambda _{\rho }(t)$ as $t\rightarrow 0$.

The following property was used in [6, 7, 21] (see [7, assumption (H2)]) for the study of eigenvalues of perturbed transfer operators in the Banach spaces introduced in [12]. Here we use it to obtain an adequate analogue for the present set-up.

### Lemma 6.1

Take $s_0 = \frac{1-\alpha _0(r_0+1)}{2r_0}$ as in (15). Let $h\in {{\mathcal {B}}}$ and $v\in C^{p_0}$. For every corridor with boundaries determined by $O_\xi$ and $O_{\xi '}$, there exists a constant $C > 0$ independent of $\rho$ and $\xi$ so that

$$\begin{aligned} \left| \int h v 1_{\{ \kappa _\rho = \xi '+ N\xi \}} \, dm \right| \le C \Vert h \Vert _s |v|_{C^{q_0}} d_{\rho }(\xi )^{\frac{3}{2}-s_0} |\xi |^{-1} \rho ^{-\frac{1}{2}+s_0} N^{-\frac{5}{2}+s_0}. \end{aligned}$$

### Proof

Let $\{ W_\ell \}_{\ell \in L}$ be the foliation of the set $\{\kappa _\rho = \xi '+\xi N\}$ into stable leaves. We can parametrise these leaves by their endpoints $(\ell , \frac{\pi }{2})$ in ${{\mathcal {S}}}_0$, then *L* is an interval of length $c \ll \frac{d_{\rho }(\xi )}{N^2 |\xi |}$ according to Lemma 2.3. The lengths of these stable leaves $|W_\ell | \le c'$ for another constant $c' \ll \sqrt{\frac{2 d_{\rho }(\xi )}{\rho N}}$, again by Lemma 2.3. The measure $dm_{W_\ell }$ is Lebesgue on the $C^1$ stable leaf $W_\ell$, and it can be parametrised as $(w_\ell (\phi ), \phi )$ where *w* is $C^1$ with $-\frac{1}{2\pi } \frac{\rho + \tau _{\min }}{\tau _{\min }}< w'(\phi ) <- \frac{1}{2\pi }$ because of the direction of the stable cones, see (13).

Let $\nu$ be a measure on *L* that produces the decomposition of Lebesgue measure *m* on $\{ \kappa _\rho = \xi '+\xi N\}$ along stable leaves. We have $\nu \ll m_L$ (and $d\nu /dm_L$ is bounded above). Since we need to partition stable leaves $W_\ell$ by the homogeneity strips ${{\mathbb {H}}}_k$ near ${{\mathcal {S}}}_0$ into pieces $W_{\ell ,k}:= W_\ell \cap {{\mathbb {H}}}_k$, we get an extra sum over $k \ge k(c'):= \lfloor (c')^{-1/r_0} \rfloor$. Then

$$\begin{aligned} \left| \int h v 1_{\{ \kappa _\rho = N\xi +\xi ' \}} \, dm\right|= & {} \left| \int _L \sum _{k \ge k(c')} \int _{W_{\ell ,k}} h\, v \, dm_{W_\ell } \ d\nu (\ell ) \right| \\\ll & {} \left| \int _L |v|_{C^{q_0}} \sum _{k \ge k(c')} \int _{W_{\ell ,k}} h\, \frac{v}{|v|_{C^{q_0}}} \, dm_{W_\ell } \ d\ell \right| \\\le & {} |v|_{C^{q_0}} \Vert h\Vert _s \int _L \left( \sum _{k \ge k(c')} |W_{\ell ,k}|^{\alpha _0-1} \int _{W_{\ell ,k}} \!\!\!\!\!\!\! \cos \phi \, \sqrt{1+|w'(\phi )|^2} \, d\phi \right) \ d\ell \\\ll & {} |v|_{C^{q_0}} \Vert h\Vert _s \int _L \sum _{k \ge k(c')} |W_{\ell ,k}|^{\alpha _0} k^{-r_0-(r_0+1)\alpha _0} \, d\ell \\\le & {} |v|_{C^{q_0}}\, \Vert h\Vert _s\, c\, k(c')^{1-\alpha _0(r_0+1)-r_0} \\\ll & {} |v|_{C^{q_0}} \Vert h\Vert _s |\xi |^{-1} d_{\rho }(\xi )^{\frac{3}{2}-s_0} \rho ^{-\frac{1}{2}+s_0} N^{-\frac{5}{2}+s_0}, \end{aligned}$$

for $s_0 = \frac{1-\alpha _0(r_0+1)}{2r_0}$, as claimed. $\square$

Using (35), Lemmas A.2 and  6.1 we obtain the asymptotics of the eigenvalue in Proposition 6.3 below.

### Lemma 6.2

For $t\in {{\mathbb {R}}}^2$, let ${\bar{A}}(t,\rho )=\sum _{|\xi | \le 1/(2\rho )} \frac{d_{\rho }(\xi )^2 \langle t, \xi \rangle ^2}{|\xi |}$. Then

$$\begin{aligned} \lim _{\rho \rightarrow 0} \frac{\rho }{2} {\bar{A}}(t,\rho ) = \frac{|t|^2}{\pi } = \langle \Sigma t,t \rangle \quad \text { for } \quad \Sigma = \begin{pmatrix} \frac{1}{\pi } &{} 0 \\ 0 &{} \frac{1}{\pi } \end{pmatrix} \quad \text { as defined in 1}. \end{aligned}$$

### Proof

The coordinate axes $p=0$ and $q=0$, and the two diagonals $p=q$ and $p=-q$ divide the plane into eight sectors. Here we count counter-clockwise with the first sector ${\Psi }_1$ directly above the positive *p*-axis. Let $\gamma = \gamma (t,\xi )$ be the angle between the vectors *t* and $\xi$. Let $\alpha = \arctan q/p$ and $\theta$ be the polar angles of $\xi$ and $t \in {{\mathbb {R}}}^2$ respectively, so $\gamma = \theta -\alpha$. For the first sector ${\Psi }_1$, taking into account that for every $\xi$ there are two $\xi '$, we have

$$\begin{aligned} \sum _{(\xi ,\xi ') \in {\Psi }_1} \frac{d_{\rho }(\xi )^2 \langle t, \xi \rangle ^2 }{|\xi |}&= 2|t|^2 \sum _{(\xi ,\xi ') \in {\Psi }_1} \frac{d_{\rho }(\xi )^2 (|\xi | \cos \gamma )^2 }{|\xi |} \\&= 2|t|^2 \sum _{(\xi ,\xi ') \in {\Psi }_1} \frac{d_{\rho }(\xi )^2 (\cos \theta \cos \alpha |\xi | + \sin \theta \sin \alpha |\xi |)^2 }{|\xi |} \\&= 2|t|^2 \sum _{(\xi ,\xi ') \in {\Psi }_1} \frac{d_{\rho }(\xi )^2 (p \cos \theta + q\sin \theta )^2 }{|\xi |}. \end{aligned}$$

The eighth sector ${\Psi }_8$ directly below the positive *p*-axis gives the same result with $-q$ instead of *q*, and sectors ${\Psi }_4$ and ${\Psi }_5$ above and below the negative *p*-axis give the same results as sectors ${\Psi }_8$ and ${\Psi }_1$. Therefore

$$\begin{aligned} \sum _{(\xi ,\xi ') \in {\Psi }_1 \cup {\Psi }_4 \cup {\Psi }_5 \cup {\Psi }_8} \frac{d_{\rho }(\xi )^2 }{|\xi |} = 4 |t|^2 \sum _{(\xi ,\xi ') \in {\Psi }_1} \frac{d_{\rho }(\xi )^2 }{|\xi |} (p^2 \cos ^2 \theta +q^2 \sin ^2 \theta ). \end{aligned}$$

The same result holds the remaining sectors with $\cos \theta$ replaced by $\sin \theta$ and vice versa. Putting the results on all eight sectors together, we get by Lemma A.4

$$\begin{aligned} \sum _{(\xi ,\xi ') \in {\Psi }} \frac{d_{\rho }(\xi )^2 \langle t, \xi \rangle ^2 }{|\xi |}= & {} |t|^2 \sum _{(\xi ,\xi ') \in {\Psi }} \frac{ (|\xi |^{-1}-2\rho )^2 }{|\xi |} (p^2+q^2) \\= & {} |t|^2 \sum _{(\xi ,\xi ') \in {\Psi }} |\xi |^{-1}-4\rho + 4\rho ^2|\xi | \\= & {} |t|^2 \frac{2\pi }{\zeta (2)} \frac{1}{2\rho } (1-\frac{2}{2}+\frac{1}{3}) (1+o(1)) = \frac{2|t|^2}{\rho \pi } (1+o(1)). \end{aligned}$$

Hence $\langle \Sigma t, t\rangle = \lim _{\rho \rightarrow 0} \frac{\rho }{2} {\bar{A}}(t,\rho ) = \frac{|t|^2}{\pi }$, as required. $\square$

For the result on the asymptotics of the eigenvalue in Proposition 6.3, we will also assume some correlation decay type results. Namely, we assume that there exist $\rho$-dependent constants ${\hat{\gamma }}_{\rho }\in (0,1)$ and ${\hat{C}}_\rho > 0$ so that for every $j\ge 1$,

36 $$\begin{aligned} \left| \int _{{{\mathcal {M}}}_0} (e^{it\kappa _\rho }-1)\, (e^{it\kappa _\rho }-1)\circ T_{\rho }^{j}\, d\mu -\Big (\int _{{{\mathcal {M}}}_0} (e^{it\kappa _\rho }-1)\,d\mu \Big )^2 \right| \le {\hat{C}}_\rho |t|^2 (1-{\hat{\gamma }}_{\rho })^j.\nonumber \\ \end{aligned}$$

More generally, we assume that that there exist $\rho$-dependent constants $\bar{\gamma }_{\rho }\in (0,1)$ and $\bar{C}_{\rho }> 0$ so that for every $j\ge 1$ and every $m\ge 0$

37 $$\begin{aligned} \Big |&\int _{{{\mathcal {M}}}_0} (e^{it\kappa _\rho }-1)\cdot R_\rho (0)^{m}(e^{it\kappa _\rho }-1)\, (e^{it\kappa _\rho }-1)\circ T_{\rho }^{j}\, d\mu \nonumber \\&-\int _{{{\mathcal {M}}}_0} (e^{it\kappa _\rho }-1) R_\rho (0)^{m}(e^{it\kappa _\rho }-1)\,d\mu \int _{{{\mathcal {M}}}_0} (e^{it\kappa _\rho }-1)\,d\mu \nonumber \\&-C\Big (\int _{{{\mathcal {M}}}_0} (e^{it\kappa _\rho }-1)\,d\mu \Big ) \int _{{{\mathcal {M}}}_0} (e^{it\kappa _\rho }-1)\, (e^{it\kappa _\rho }-1)\circ T_{\rho }^{j}\, d\mu \nonumber \\&+C\Big (\int _{{{\mathcal {M}}}_0} (e^{it\kappa _\rho }-1)\,d\mu \Big )^3\Big |\le \bar{C}_{\rho }|t|^2 (1-\bar{\gamma }_{\rho })^{m+j}, \end{aligned}$$

where $C=0$ if $m=0$ and $C=1$ if $m\ge 1$. As justified in Proposition C.1 in Appendix C via the argument used in [11, Proof of Proposition 9.1], assumptions (36) and (37) are natural.

### Proposition 6.3

Assume (32), (36) and (37), and let ${\bar{A}}(t,\rho )$ be as defined in Lemma 6.2. Let $\nu \in (\frac{1}{3},\frac{1}{2})$ and $\delta \in (0,\frac{1}{2}\min \{\gamma _{\rho },\hat{\gamma }_{\rho }\})$, ensuring that (33) holds. Then for any $\delta _0\le \delta ^{4/(3\nu -1)}$ and $t\in B_{\delta _0}(0)$,

$$\begin{aligned} 1-\lambda _{\rho }(t)={\bar{A}}(t,\rho ) \frac{\log (1/|t|)}{8\pi \rho }+E(t, \rho ), \end{aligned}$$

where $|E(t,\rho )|\le \bar{C}_{\rho }\,\bar{\gamma }_{\rho }^{-2}\,|t|^2 + C|t|^2 \rho ^{-2}$ for $\bar{C}_{\rho }$ and $\bar{\gamma }_{\rho }$ as in (37) and some uniform constant *C*.

### Remark 6.4

It is possible to shrink $\delta _0$ further to $\delta _0<e^{-\max \{\bar{C}_{\rho }\bar{\gamma }_{\rho }^{-2},\rho ^{-2}\}}$ leading to $E(t,\rho )=o(|t|^2\log |1/t|)$. This would mean that in the proof of main results in Sect. 7 we would work on this very small neighborhood and obtain the same range of *n* and $\rho$ in the final statements. We find it more convenient to work on the neighborhood $B_{\delta _0}(0)$ as in the statement above.

### Remark 6.5

Let $q_{\rho }$ be the flight function taking values in ${{\mathbb {R}}}^2$ as opposed to the displacement function $\kappa _\rho$ taking values in ${{\mathbb {Z}}}^2$. A similar statement holds for the dominant eigenvalue of the perturbed operator $R_\rho (e^{it q_{\rho }})$. The proof is similar to the one below using that $|q_{\rho }-\kappa _\rho | \le 1$.

### Proposition 6.3

In the notation of Banach spaces of distributions (see, for instance, [21]) for $h\in C^{q_0}$ we write $\langle h, \textbf{1}\rangle =\langle \textbf{1}, h\rangle =\int h \textbf{1}\, d m$ and $\langle m, h\rangle =\int h\, d m$, where $\textbf{1}$ is both an element of ${{\mathcal {B}}}$ and of $(C^{q_0})'$. Let $v_{\rho }(t)=\frac{ \Pi _{\rho }(t) 1}{\langle \Pi _{\rho }(t) 1, 1\rangle }$ and recall that $v_{\rho }(0)=\textbf{1}$. Recall also that for every $\rho$, $\lambda _{\rho }(t) v_\rho (t)={\hat{R}}_{\rho }(t) v_{\rho }(t)$ for *t* small enough, and that $\lambda _{\rho }(0)=1$. Since $\langle v_{\rho }(t),\textbf{1}\rangle =1$,

$$\begin{aligned} 1-\lambda _{\rho }(t)&=1-\langle {\hat{R}}_{\rho }(t)v_\rho (t),\textbf{1}\rangle =\mu (1-e^{it\kappa _\rho })+\langle ({\hat{R}}_{\rho }(t)-{\hat{R}}_{\rho }(0))(v_{\rho }(t)-\textbf{1}),\textbf{1}\rangle \\&=: \mu (1-e^{it\kappa _\rho })+ V(t,\rho ). \end{aligned}$$

With the meaning of inner product clarified, for ease of notation from here on we will write $V(t,\rho )=\int _{{{\mathcal {M}}}}(e^{it\kappa _\rho }-1)(v_{\rho }(t)-\textbf{1}) \, d m$. We recall the terminology in Remark 2.2. For $\xi = (p,q)$ with $\gcd (p,q) = 1$, we let $\xi ' = (p',q')$ be the point uniquely determined by $\xi$ in the sense that $p'/q'$ is convergent preceding *p*/*q* in the continued fraction expansion of *p*/*q*; in particular $|\xi '| \le |\xi |$. Recall that ${\Psi }$ is the set of all such pairs $(\xi ,\xi ')$ with $|\xi | \le 1/(2\rho )$. With this specified, we write

$$\begin{aligned} \mu (1-e^{it\kappa _\rho })=\sum _{(\xi ,\xi ')\in {\Psi }}\sum _{N=1}^\infty (e^{it( \xi '+ N\xi )}-1) \mu (\{ \kappa _\rho = \xi '+ N\xi \}). \end{aligned}$$

Using the fact that $\int \kappa _\rho \, d\mu =0$, we compute that

$$\begin{aligned} \mu (1-e^{it\kappa _\rho })&=\sum _{(\xi ,\xi ')\in {\Psi }}\sum _{N=1}^\infty \left( e^{it( \xi '+ N\xi )}-1-it( \xi '+ N\xi )\right) \mu (\{ \kappa _\rho = \xi '+ N\xi \})\\&=\sum _{(\xi ,\xi ')\in {\Psi }}\sum _{N=1}^{1/|t|} \left( e^{it( \xi '+ N\xi )}-1-it( \xi '+ N\xi )\right) \mu (\{ \kappa _\rho = \xi '+ N\xi \}) \\&\quad +O\left( |t|\sum _{(\xi ,\xi ')\in {\Psi }}|\xi |\sum _{N>1/|t|} N\mu (\{ \kappa _\rho = \xi '+ N\xi \}) \right) \\&=\sum _{(\xi ,\xi ')\in {\Psi }}\sum _{N=1}^{1/|t|} \frac{1}{2}\, \langle t, \xi '+ N\xi \rangle ^2 \mu (\{ \kappa _\rho = \xi '+ N\xi \}) +O(|t|^2)\\ {}&:=I(t,\rho )+O(|t|^2), \end{aligned}$$

where the involved constants in the last big *O* are independent of $\rho$. Further, using Lemma A.4,

$$\begin{aligned} I(t,\rho )&=\frac{1}{4\pi \rho }\sum _{|\xi | \le 1/(2\rho )}\frac{d_{\rho }(\xi )^2}{|\xi |} \langle t, \xi \rangle ^2\sum _{N=\max \{1,d_\rho (\xi )/(2\rho )\}}^{1/|t|} \frac{1}{N} \\&\qquad +O\left( |t|^2\sum _{(\xi ,\xi ')\in {\Psi }}\frac{1}{4\pi |\xi |\rho } \sum _{N<\max \{1, d_\rho (\xi )/(2\rho )\}}4\rho ^2\, N\,|\xi |\right) \\&=\frac{1}{4\pi \rho }\sum _{|\xi | \le 1/(2\rho )}\frac{d_{\rho }(\xi )^2}{|\xi |}\langle t, \xi \rangle ^2 \sum _{N=\max \{1,d_\rho (\xi )/(2\rho )\}}^{1/|t|} \frac{1}{N} +O\left( |t|^2\rho ^{-1}\right) \\&=\frac{\log (1/|t|)}{4\pi \rho }\sum _{|\xi | \le 1/(2\rho )}\frac{d_{\rho }(\xi )^2}{|\xi |}\langle t, \xi \rangle ^2 + O\left( |t|^2\rho ^{-1}\log (1/\rho )\right) . \end{aligned}$$

Hence, with ${\bar{A}}(t,\rho )$ as in Lemma 6.2,

$$\begin{aligned} \mu (1-e^{it\kappa _\rho })={\bar{A}}(t,\rho ) \frac{\log (1/|t|)}{4\pi \rho }+O\left( |t|^2\rho ^{-1}\log (1/\rho )\right) . \end{aligned}$$

Thus, $1-\lambda _{\rho }(t)={\bar{A}}(t,\rho ) \frac{\log (1/|t|)}{4\pi \rho }+E(t,\rho )$, where $E(t,\rho )=O\left( |t|^2\rho ^{-1}\log (1/\rho )\right) + V(t,\rho )$. It remains to estimate $V(t,\rho )$. Note that

$$\begin{aligned} v_{\rho }(t)-\textbf{1}=\frac{\mu ((\Pi _{\rho }(t)-\Pi _{\rho }(0)) \textbf{1})}{\mu ( \Pi _{\rho }(t) \textbf{1})}\, \Pi _\rho (0)\textbf{1}+\frac{(\Pi _{\rho }(t)-\Pi _{\rho }(0)) \textbf{1}}{\mu _{\rho }( \Pi _{\rho }(t) 1)}. \end{aligned}$$

Hence,

$$\begin{aligned} V(t,\rho )= & {} \frac{\mu ((\Pi _{\rho }(t)-\Pi _{\rho }(0)) \textbf{1})}{\mu ( \Pi _{\rho }(t) \textbf{1})}\int _{{{\mathcal {M}}}_0} (e^{it\kappa _\rho }-1)\, d \mu \\{} & {} +\frac{\int _{{{\mathcal {M}}}_0} (e^{it\kappa _\rho }-1) (\Pi _{\rho }(t)-\Pi _{\rho }(0)) \textbf{1}\, d m}{\mu ( \Pi _{\rho }(t) \textbf{1})}\\= & {} I_1(t,\rho )+I_2(t,\rho ). \end{aligned}$$

*Estimating* $I_1(t,\rho )$. Since $\int _{{{\mathcal {M}}}_0} \kappa _\rho \, d\mu =0$, we have

$$\begin{aligned} I_1(t,\rho )=\frac{\mu ((\Pi _{\rho }(t)-\Pi _{\rho }(0)) \textbf{1})}{\mu ( \Pi _{\rho }(t) \textbf{1})}\int _{{{\mathcal {M}}}_0} (e^{it\kappa _\rho }-1-it\kappa _\rho )\, d \mu . \end{aligned}$$

Now, by (35) and Lemma 6.1,

38 $$\begin{aligned} \int _{{{\mathcal {M}}}} | (\Pi _{\rho }(t)-\Pi _{\rho }(0))\textbf{1}|\, d\mu&=\sum _{(\xi ,\xi ')\in {\Psi }}\sum _{N=1}^\infty \int _{{{\mathcal {M}}}} 1_{\{ \kappa _\rho = \xi '+ N\xi \}} |(\Pi _{\rho }(t)-\Pi _{\rho }(0)) \textbf{1}| \nonumber \\&\le \sum _{(\xi ,\xi ')\in {\Psi }} |\xi |^{-\frac{5}{2}+s_0} \rho ^{-\frac{1}{2}+s_0}\Vert \Pi _{\rho }(t)-\Pi _{\rho }(0)\Vert _s\sum _{N=1}^\infty N^{-\frac{5}{2}} \nonumber \\&\le C \rho ^{-\nu } \gamma _{\rho }^{-2}|t|^{\nu } \end{aligned}$$

for some uniform *C*. Using also that $|e^{ix}-1-ix|\le x^y$, for any $y\in (0,2]$,

$$\begin{aligned} |I_1(t,\rho )|\le C \rho ^{-\nu } \gamma _{\rho }^{-2}|t|^{\nu }|t|^{2-\nu /2}\int _{{{\mathcal {M}}}_0} |\kappa _\rho |^{2-\nu }\, d \mu \le C \rho ^{-\nu -1} \gamma _{\rho }^{-2}|t|^{\nu /2+2}, \end{aligned}$$

where in the last inequality we have used Lemma A.5. Note that for $|t|\in B_{\delta _0}(0)$ with $\delta _0\le \gamma _{\rho }^{4/(3\nu -1)}$, as in the statement, $|t|^{\nu /2}<\gamma _{\rho }^{2\nu /(3\nu -1)}< \gamma _{\rho }^{2}$ for all $\nu \in (\frac{1}{3},\frac{1}{2})$. Thus, $|I_1(t,\rho )|\le C \rho ^{-\nu -1} |t|^{2}$.

*Estimating* $I_2(t,\rho )$ Recall that (32) holds and that $\delta$ is chosen so that (34) holds. Using the definition of $\Pi _{\rho }(t)$ and noting that for every $\rho$, $(u-{\hat{R}}_{\rho }(0))^{-1}\textbf{1}=(1-u)^{-1}$,

$$\begin{aligned} (\Pi _{\rho }(t)-\Pi _{\rho }(0) 1&=\int _{|u-1|=\delta }(u-{\hat{R}}_{\rho }(t))^{-1}({\hat{R}}_{\rho }(t)-{\hat{R}}_{\rho }(0))(u-{\hat{R}}_{\rho }(0))^{-1}\textbf{1}\, du\\&=\int _{|u-1|=\delta }(1-u)^{-1}(u-{\hat{R}}_{\rho }(t)^{-1}({\hat{R}}_{\rho }(t)-{\hat{R}}_{\rho }(0))\textbf{1}\, du. \end{aligned}$$

Thus,

39 $$\begin{aligned} I_2(t,\rho )&=\int _{{{\mathcal {M}}}_0}(e^{it\kappa _\rho }-1)\int _{|u-1|=\delta }(1-u)^{-1}(u-{\hat{R}}_{\rho }(t))^{-1}({\hat{R}}_{\rho }(t)-{\hat{R}}_{\rho }(0))\textbf{1}\, d u\, d m \nonumber \\&:=J_1(t,\rho )+J_2(t,\rho ), \end{aligned}$$

for

$$\begin{aligned} J_1(t,\rho ):=\int _{{{\mathcal {M}}}_0}(e^{it\kappa _\rho }-1)\int _{|u-1|=\delta }(1-u)^{-1}(u-{\hat{R}}_{\rho }(0))^{-1}({\hat{R}}_{\rho }(t)-{\hat{R}}_{\rho }(0))\textbf{1}\, d u\, d m \end{aligned}$$

and

$$\begin{aligned} J_2(t,\rho )&:= \int _{{{\mathcal {M}}}_0}(e^{it\kappa _\rho }-1)\int _{|u-1|=\delta }(1-u)^{-1}\left( (u-{\hat{R}}_{\rho }(t))^{-1}-(u-{\hat{R}}_{\rho }(0))^{-1}\right) \\&\qquad ({\hat{R}}_{\rho }(t)-{\hat{R}}_{\rho }(0))\textbf{1}\, d u\, d m\\&= \int _{{{\mathcal {M}}}_0}(e^{it\kappa _\rho }-1)\int _{|u-1|=\delta } (1-u)^{-1}(u-{\hat{R}}_{\rho }(t))^{-1}({\hat{R}}_{\rho }(t)-{\hat{R}}_{\rho }(0)) (u-{\hat{R}}_{\rho }(0))^{-1} \\&\qquad \times ({\hat{R}}_{\rho }(t)-{\hat{R}}_{\rho }(0))\textbf{1}\, d u\, d m =: K_1(t,\rho )+K_2(t,\rho ), \end{aligned}$$

where

40 $$\begin{aligned} K_1(t,\rho )=\int _{{{\mathcal {M}}}_0}(e^{it\kappa _\rho }-1)\int _{|u-1|=\delta }&(1-u)^{-1}(u-{\hat{R}}_{\rho }(0))^{-1}({\hat{R}}_{\rho }(t)-{\hat{R}}_{\rho }(0)) \nonumber \\&\times (u-{\hat{R}}_{\rho }(0))^{-1}({\hat{R}}_{\rho }(t)-{\hat{R}}_{\rho }(0))\textbf{1}\, d u\, d m \end{aligned}$$

and

$$\begin{aligned} \nonumber K_2(t,\rho )=\int _{{{\mathcal {M}}}_0}(e^{it\kappa _\rho }-1)&\int _{|u-1|=\delta } (1-u)^{-1} \left( (u-{\hat{R}}_{\rho }(t))^{-1}-(u-{\hat{R}}_{\rho }(0))^{-1}\right) \\&\times ({\hat{R}}_{\rho }(t)-{\hat{R}}_{\rho }(0)) (u-{\hat{R}}_{\rho }(0))^{-1}({\hat{R}}_{\rho }(t)-{\hat{R}}_{\rho }(0))\textbf{1}\, d u\, d m. \end{aligned}$$

We first treat $K_2(t,\rho )$. Note that for *u* in the chosen contour, $\Vert (u-R_\rho (t))^{-1}\Vert _{{{\mathcal {B}}}}\le \gamma _{\rho }^{-1}$. Using (35), for all such *u*,

$$\begin{aligned}&\left\| \left( (u-{\hat{R}}_{\rho }(t))^{-1}-(u-{\hat{R}}_{\rho }(0))^{-1}\right) ({\hat{R}}_{\rho }(t)-{\hat{R}}_{\rho }(0)) (u-{\hat{R}}_{\rho }(0))^{-1}({\hat{R}}_{\rho }(t)-{\hat{R}}_{\rho }(0))\right\| _{{{\mathcal {B}}}} \\&\quad \le C \rho ^{-2\nu }|t|^{3\nu }\gamma _{\rho }^{-3}. \end{aligned}$$

This together with Lemma 6.1 gives that

$$\begin{aligned} |K_2(t,\rho )|&\le \sum _{(\xi ,\xi ')\in {\Psi }} \sum _{N=1}^\infty \int _{{{\mathcal {M}}}_0} \int _{|u-1|=\delta }|1-u|^{-1}1_{\{ \kappa _\rho = \xi '+ N\xi \} } |e^{it\kappa _\rho }-1|\\&\quad \times \left| (u-{\hat{R}}_{\rho }(t))^{-1}-(u-{\hat{R}}_{\rho }(0))^{-1} ({\hat{R}}_{\rho }(t)-{\hat{R}}_{\rho }(0) (u-{\hat{R}}_{\rho }(0))^{-1}({\hat{R}}_{\rho }(t)-{\hat{R}}_{\rho }(0)) \textbf{1}\right| \, d u\, d m\\&\le |t|^{3\nu } \rho ^{-3\nu } \gamma _{\rho }^{-3} \sum _{(\xi ,\xi ')\in {\Psi }} |\xi |^{-\frac{5}{2}+s_0} \rho ^{-\frac{1}{2}+s_0}\sum _{N=1}^\infty |t| N^{-\frac{3}{2}}\le C \rho ^{-3\nu } \gamma _{\rho }^{-3}|t|^{3\nu +1}. \end{aligned}$$

Hence, $|K_2(t,\rho )| \le C \rho ^{-1}\gamma _{\rho }^{-3}\,|t|^{2}\, t^{3\nu -1}=C \rho ^{-1}\gamma _{\rho }^{-3}\,|t|^{2}\, \gamma _{\rho }^4$ for all $|t|\in B_{\delta _0}$ with $\delta _0<\gamma _{\rho }^{4/(3\nu -1)}$. It follows that $|K_2(t,\rho )| \le C \rho ^{-1}|t|^{2}$.

*Estimating* $J_1(t,\rho )$ *in* (39) *and* $K_1(t,\rho )$ *in* (40) These terms are in, some sense, independent of the Banach space ${{\mathcal {B}}}$ (see the explanation below) and can be analysed either via the correlation function (36) or its generalization (37). The rest of the proof is allocated to this type of analysis.

We start with $J_1(t,\rho )$ defined in (39), which is easier using (36). Recall that ${\hat{R}}_{\rho }(0)=R_{\rho }$ and $\int _{|u-1|=\delta } (1-u)^{-2} \, du=0$ due to Cauchy’s theorem. This gives

$$\begin{aligned} J_1(t,\rho )&=\int _{{{\mathcal {M}}}_0}(e^{it\kappa _\rho }-1)\int _{|u-1|=\delta }(1-u)^{-1}\sum _{j=0}^\infty u^{-j-1} R_{\rho }^j\, R_\rho (e^{it\kappa _\rho }-1)\textbf{1}\, d u\, d m \\&\quad -\left( \int _{{{\mathcal {M}}}_0}(e^{it\kappa _\rho }-1)\, d \mu \right) ^2 \int _{|u-1|=\delta }(1-u)^{-1}\sum _{j=0}^\infty u^{-j-1}\, d u\\&=\int _{|u-1|=\delta }(1-u)^{-1}\sum _{j=0}^\infty u^{-j-1}\int _{{{\mathcal {M}}}_0}(e^{it\kappa _\rho }-1) R_\rho ^j\, R_{\rho }(e^{it\kappa _\rho }-1)\textbf{1}\, d m\, d u \\&\quad -\left( \int _{{{\mathcal {M}}}_0}(e^{it\kappa _\rho }-1)\, d \mu \right) ^2 \int _{|u-1|=\delta }(1-u)^{-1}\sum _{j=0}^\infty u^{-j-1}\, d u. \end{aligned}$$

Swapping the order of the integrals is allowed due to (36). The quantity

$$\begin{aligned} \Big (\int _{{{\mathcal {M}}}_0} (e^{it\kappa _\rho }-1) R_\rho ^{j+1}(e^{it\kappa _\rho }-1)\, d\mu -\int _{{{\mathcal {M}}}_0}(e^{it\kappa _\rho }-1)\, d \mu \Big )^2 \end{aligned}$$

decays exponentially fast. Hence, we can write

$$\begin{aligned} J_1(t,\rho )&=\int _{|u-1|=\delta }(1-u)^{-1}\sum _{j=0}^\infty u^{-j-1}\\&\quad \times \Big (\int _{{{\mathcal {M}}}_0} (e^{it\kappa _\rho }-1)\,(e^{it\kappa _\rho }-1)\circ T_{\rho }^{j+1} \, d\mu -\left( \int _{{{\mathcal {M}}}_0}(e^{it\kappa _\rho }-1)\, d \mu \right) ^2 \Big )\, du. \end{aligned}$$

Using Lemma A.5 to control the dependence on $\rho$, $\left( \int _{{{\mathcal {M}}}_0}(e^{it\kappa _\rho }-1)\, d \mu \right) ^2\le C |t|^2\rho ^{-2}$. Next, recall that (32) holds and that $\delta < \frac{1}{2} \min \{ \gamma _{\rho }, {\hat{\gamma }}_{\rho } \}$. Note that for $|u-1|=\delta$, we have $|u|^{-(j+1)}\ll (1-{\hat{\gamma }}_{\rho }/2)^{-(j+1)}$. This together with (36) gives

$$\begin{aligned} |J_1(t,\rho )|&\le C_\rho \,|t|^2 \int _{|u-1|=\delta }|1-u|^{-1}\sum _{j=0}^\infty |u|^{-j-1}\left( 1-{\hat{\gamma }}_{\rho }\right) ^{j+1}\\&\ll {\hat{C}}_\rho \,|t|^2 \sum _{j=1}^\infty \left( \frac{1-{\hat{\gamma }}_{\rho }}{1-{\hat{\gamma }}_{\rho }/2}\right) ^{j+1}\le 2{\hat{C}}_\rho \,|t|^2\, {\hat{\gamma }}_{\rho }^{-1}. \end{aligned}$$

An argument similar to the one above used in estimating $J_1(t,\rho )$ with  (37) instead of (36) allows us to deal with $K_1(t,\rho )$ defined in (40). Compute that

$$\begin{aligned} K_1(t,\rho )=\int _{{{\mathcal {M}}}_0}(e^{it\kappa _\rho }-1)\int _{|u-1|=\delta }&(1-u)^{-1}\sum _{m\ge 1} u^{-m}\sum _{j\ge 1} u^{-j}{\hat{R}}_{\rho }(0)^{j}(e^{it\kappa _\rho }-1)\\&\times {\hat{R}}_{\rho }(0)^{m}(e^{it\kappa _\rho }-1)\, d u\, d m. \end{aligned}$$

Let

$$\begin{aligned} E(t,\rho )&=\int _{{{\mathcal {M}}}_0} (e^{it\kappa _\rho }-1)\,d\mu \,\int _{|u-1|=\delta }(1-u)^{-1}\\&\qquad \times \sum _{j\ge 1} u^{-j}\sum _{m\ge 1} u^{-m}\int _{{{\mathcal {M}}}_0} (e^{it\kappa _\rho }-1) R_\rho (0)^{m}(e^{it\kappa _\rho }-1)\,d\mu \, du \\&-\int _{|u-1|=\delta }(1-u)^{-1}\sum _{j\ge 1} u^{-j}\sum _{m\ge 1} u^{-m}\int _{{{\mathcal {M}}}_0} (e^{it\kappa _\rho }-1)\,d\mu \, \\&\qquad \times \int _{{{\mathcal {M}}}_0} (e^{it\kappa _\rho }-1)\, (e^{it\kappa _\rho }-1)\circ T_{\rho }^{j}\, d\mu \, du\\&-\Big (\int _{{{\mathcal {M}}}_0} (e^{it\kappa _\rho }-1)\,d\mu \Big )^3\int _{|u-1|=\delta }(1-u)^{-1}\sum _{j\ge 1} u^{-j}\sum _{m\ge 1} u^{-m}\, du\\&=(E_1(t,\rho )-E_2(t,\rho ))\,\int _{{{\mathcal {M}}}_0} (e^{it\kappa _\rho }-1)\,d\mu -E_3(t,\rho ). \end{aligned}$$

Using (37), we obtain

$$\begin{aligned} \Big |K_1(t,\rho ) -E(t,\rho ) \Big |\le \bar{C}_{\rho }|t|^2 \sum _{m\ge 1} |u|^{-m}\sum _{j\ge 1} |u|^{-j}(1-\bar{\gamma }_{\rho })^{m+j} \le 4 \bar{C}_{\rho }|t|^2 \bar{\gamma }_{\rho }^{-2}, \end{aligned}$$

where in the last inequality we proceeded as in estimating $J_1$ above.

Finally, we need to argue that *E* is bounded by $|t|^2$. First,

$$\begin{aligned}&E_1(t,\rho )=\int _{|u-1|=\delta }(1-u)^{-1}\sum _{j\ge 1} u^{-j}\sum _{m\ge 1} u^{-m}\int _{{{\mathcal {M}}}_0} (e^{it\kappa _\rho }-1) R_\rho (0)^{m}(e^{it\kappa _\rho }-1)\,d\mu \, du\\&\quad =\int _{|u-1|=\delta }(1-u)^{-2}\sum _{m\ge 1} u^{-m}\int _{{{\mathcal {M}}}_0} (e^{it\kappa _\rho }-1)\,(e^{it\kappa _\rho }-1)\circ T_\rho ^m\,d\mu \, du\\&\quad =\int _{|u-1|=\delta }(1-u)^{-2}\sum _{m\ge 1} u^{-m} \\&\qquad \times \left( \int _{{{\mathcal {M}}}_0} (e^{it\kappa _\rho }-1)\,(e^{it\kappa _\rho }-1)\circ T_\rho ^m\,d\mu \, du - \left( \int _{{{\mathcal {M}}}_0} (e^{it\kappa _\rho }-1)\,d\mu \right) ^2\,d\mu \right) \\&\qquad + \left( \int _{{{\mathcal {M}}}_0} (e^{it\kappa _\rho }-1)\,d\mu \right) ^2\int _{|u-1|=\delta }(1-u)^{-2}\sum _{m\ge 1} u^{-m}\, du= E_1^1(t,\rho )+E_1^2(t,\rho ). \end{aligned}$$

Using (36), we have that $|E_1^1(t,\rho )|\le 2{\hat{C}}_\rho \,|t|^2\, {\hat{\gamma }}_{\rho }^{-1}$.

Also, $E_2(t,\rho )=\int _{|u-1|=\delta }(1-u)^{-2}\sum _{j\ge 1} u^{-j}\int _{{{\mathcal {M}}}_0} (e^{it\kappa _\rho }-1)\, (e^{it\kappa _\rho }-1)\circ T_{\rho }^{j}\, d\mu$ and again by (36) and Cauchy’s theorem, $| E_2(t,\rho )|\le 4{\hat{C}}_\rho \,|t|^2\, {\hat{\gamma }}_{\rho }^{-2}$. Finally, $E_3(t,\rho )=0$. Altogether, $| K_1(t,\rho )|\le 8\bar{C}_{\rho }\,|t|^2\, \bar{\gamma }_{\rho }^{-2}$. $\square$

## Limit theorems and mixing as $\rho \rightarrow 0$

The first result below is the non-standard Gaussian limit law, known to hold when the horizon is infinite. It is a precise version of Theorem A stated in Sect. 1.3.

Our main contribution lies in characterizing the limit paths allowed as $\rho \rightarrow 0$; this is done up to the unknown $\gamma _{\rho }$, $C_\rho$ in (32) and $\bar{C}_{\rho }$, $\bar{\gamma }_{\rho }$ as in (37).

Throughout this section, the notation is the same in Sect. 1.1. In particular, $b_{n,\rho }=\frac{\sqrt{n\log (n/\rho ^2)}}{\sqrt{4\pi }\ \rho }$, and the variance matrix $\Sigma$ are defined as in (1), in agreement with Lemma 6.2. We recall that $\implies$ stands for convergence in distribution with respect to the invariant measure $\mu$.

### Theorem 7.1

Let $\gamma _{\rho }$, $C_\rho$ be as in (32), let $\bar{\gamma }_{\rho }$, $\bar{C}_{\rho }$ be as in (37) and let *C* be as in Proposition 6.3.

Set $M(\rho )=\max \{ C_\rho \gamma _{\rho }^{-2},\rho ^2 \bar{C}_{\rho }\bar{\gamma }_{\rho }^{-2}\} + C$. Let $\rho \rightarrow 0$ and simultaneously $n\rightarrow \infty$ in such a way that $M(\rho )= o(\log n)$. Then

$$\begin{aligned} \frac{\kappa _{n,\rho }}{b_{n,\rho }}\implies {{\mathcal {N}}}(0,\Sigma ). \end{aligned}$$

### Remark 7.2

A similar statement holds for the flight function $q_{\rho }$. The only change in the proof is the use of Remark 6.5 instead of Proposition 6.3.

### Proof

Throughout we let $\delta <\frac{1}{2} \min \{\gamma _{\rho },{\hat{\gamma }}_{\rho }\}$, so that we can use Proposition 6.3 with $\delta _0=\delta ^{4/(3\nu -1)}$. By (33), for $t\in B_{\delta _0}(0)$,

$$\begin{aligned} {{\mathbb {E}}}_\mu (e^{i t\kappa _{n,\rho }} 1)&={{\mathbb {E}}}_\mu ({\hat{R}}_{\rho }(t)^n 1)=\lambda _{\rho }(t)^n\int _{{{\mathcal {M}}}_0}\Pi _{\rho }(t) 1\, d\mu +\int _{{{\mathcal {M}}}_0}Q_{\rho }(t)^n1\, d\mu \\&=\lambda _{\rho }(t)^n\int _{{{\mathcal {M}}}_0}\Pi _{\rho }(t) 1\, d\mu +O(C_\rho \,(1-\gamma _{\rho })^n). \end{aligned}$$

Note that the assumption $M(\rho )=o(\log n)$ ensures that, for $\rho$ small enough, $\frac{t}{b_{n,\rho }}\in B_{\delta _0}(0)$ for all $t\in {{\mathbb {R}}}^2$. Hence, as $n\rightarrow \infty$ and given the range of *n*, equivalently as $\rho \rightarrow 0$,

$$\begin{aligned} \left| {{\mathbb {E}}}_\mu \left( \exp \left( {it \frac{\kappa _{n,\rho }}{b_{n,\rho }}}\right) \right) - \lambda _{\rho } \left( \frac{t}{b_{n,\rho }}\right) ^n\int _{{{\mathcal {M}}}_0}\Pi _{\rho } \left( \frac{t}{b_{n,\rho }}\right) 1\, d\mu \right| \rightarrow 0, \end{aligned}$$

for all $$t\in {{\mathbb {R}}}^2$$.

Also, it follows from (35) that $\Vert \Pi _{\rho }\left( \frac{t}{b_{n,\rho }}\right) -\Pi _{\rho }(0) \Vert _{{{\mathcal {B}}}}\rightarrow 0$, as $n\rightarrow \infty$ and given the range of *n*, equivalently as $\rho \rightarrow 0$. Thus, a standard argument based on the dominated convergence theorem shows that as $n\rightarrow \infty$, equivalently as $\rho \rightarrow 0$,

$$\begin{aligned} \left| {{\mathbb {E}}}_\mu \left( \exp \left( {it \, \frac{\kappa _{n,\rho }}{b_{n,\rho }}}\right) \right) - \lambda _{\rho } \left( \frac{t}{b_{n,\rho }}\right) ^n\right| \rightarrow 0. \end{aligned}$$

It remains to understand $\lambda _{\rho } \left( \frac{t}{b_{n,\rho }}\right) ^n$ as $\rho \rightarrow 0$. Since $\delta _0=\delta ^{4/(3\nu -1)}$, we can apply Proposition 6.3 to obtain

$$\begin{aligned}&n\left( 1-\lambda _{\rho } \left( \frac{t}{b_{n,\rho }}\right) \right) \\ {}&\quad =\frac{n}{8\pi \rho }\,{\bar{A}}\left( \frac{t}{b_{n,\rho }}\,,\,\rho \right) \log (b_{n,\rho }/|t|) +n\, O\left( \left( \bar{C}_{\rho }\bar{\gamma }_{\rho }^{-2} + C\rho ^{-2}\right) \left( \frac{|t|}{b_{n,\rho }}\right) ^2\right) . \end{aligned}$$

By assumption, $M(\rho )=o(\log n)$. Hence, as $\rho \rightarrow 0$,

$$\begin{aligned}&n\, \left( \bar{C}_{\rho }\bar{\gamma }_{\rho }^{-2} + C\rho ^{-2}\right) \left( \frac{|t|}{b_{n,\rho }}\right) ^2\\ {}&\quad = \left( \bar{C}_{\rho }\bar{\gamma }_{\rho }^{-2} + C\rho ^{-2}\right) \frac{4\pi |t|^2\rho ^2}{\log (n/\rho ^2)} =O\left( \frac{M(\rho )}{\log n}\right) \cdot |t|^2= o(1)\cdot |t|^2\rightarrow 0. \end{aligned}$$

Now, given that ${\bar{A}}$ is as in Lemma 6.2,

$$\begin{aligned} \frac{n}{4\pi \rho }\,{\bar{A}}\left( \frac{t}{b_{n,\rho }},\,\rho \right) =\frac{1}{\log (n/\rho ^2)}\frac{1}{\rho }\, \rho ^2\bar{A}\left( t,\rho \right) =\frac{\rho {\bar{A}}(t,\rho )}{\log (n/\rho ^2)}. \end{aligned}$$

Also, using Lemma 6.2 and recalling the range of *n*,

$$\begin{aligned} \lim _{\rho \rightarrow 0}\,&\frac{n}{4\pi \rho }\,\bar{A}\left( \frac{t}{b_{n,\rho }}\, ,\, \rho \right) \log \left( \frac{b_{n,\rho }}{|t|}\right) = \lim _{\rho \rightarrow 0} \frac{\rho {\bar{A}}\left( t,\rho \right) }{\log (n/\rho ^2)}\log \left( \frac{b_{n,\rho }}{|t|}\right) \\&= \lim _{\rho \rightarrow 0}\frac{\rho }{2} \frac{{\bar{A}}(t,\rho )}{\log \left( \frac{\sqrt{n}}{\rho }\right) } \log \left( \frac{\sqrt{n}}{\rho } \frac{\sqrt{\log (n/\rho ^2)} }{ \sqrt{4\pi } |t|} \right) =\langle \Sigma t, t\rangle , \end{aligned}$$

where in the last equality we have used Lemma 6.2 and the uniform convergence theorem for slowly varying functions. Putting the above together,

41 $$\begin{aligned} \lim _{\rho \rightarrow 0} \lambda _{\rho } \left( \frac{t}{b_{n,\rho }}\right) ^n =\lim _{\rho \rightarrow 0} \exp \left( - n \left( 1-\lambda _{\rho } \left( \frac{t}{b_{n,\rho }}\right) \right) \right) =\exp \left( -\frac{1}{2}\langle \Sigma t, t\rangle \right) , \end{aligned}$$

for any $t\in {{\mathbb {R}}}^2$. This completes the proof of Theorem 7.1 by Levy’s continuity theorem. $\square$

The next result gives a local limit theorem as $\rho \rightarrow 0$, again up to the unknown $\gamma _{\rho }$, $C_\rho$ and $\bar{\gamma }_{\rho }$, $\bar{C}_{\rho }$. This is possible due to the present proof based on spectral methods which produces the fine control of the eigenvalue in Proposition 6.3. The present proof of local limit theorem for the infinite horizon is new even for $\rho$ fixed. We recall that the only proof of such a local limit is given in [31] via the abstract results in [4] for Young towers. Our proof relies on Proposition 6.3, which is new in the set-up of the Banach spaces considered here and it relies heavily on Appendix C and on Proposition 3.1 (which provides useful continuity estimates).

In the notation of Theorem 7.1 we let $\Phi _\Sigma$ be the density of a Gaussian random variable distributed according to ${{\mathcal {N}}}(0,\Sigma )$ and recall from Sect. 4.1 that $C^{p_0}\subset {{\mathcal {B}}}$.

### Theorem 7.3

Assume the assumptions and notation of Theorem 7.1; in particular $M(\rho )$ is defined in the same way. Let $v\in C^{p_0}({{\mathcal {M}}})$ and $w\in L^a({{\mathcal {M}}})$, for $a>1$.

Let $\rho \rightarrow 0$ and simultaneously $n\rightarrow \infty$ in such a way that $M(\rho )=o(\log n)$. Then

$$\begin{aligned} \left| \int _{{{\mathcal {M}}}} v1_{\{\kappa _{n,\rho }=N\}} w\circ T_\rho ^n\, d\mu -\frac{{{\mathbb {E}}}_\mu (v)\,{{\mathbb {E}}}_\mu (w)}{(b_{n,\rho })^2}\, \Phi _\Sigma \left( \frac{N}{b_{n,\rho }}\right) \right| \rightarrow 0. \end{aligned}$$

uniformly in $$N\in {{\mathbb {Z}}}^2$$.

### Remark 7.4

A similar statement holds for the flight function $q_{\rho }$. By a similar argument, using Remark 6.5 instead of Proposition 6.3, we obtain $(b_{n,\rho })^2\mu (\{q_{n,\rho }\in V\})\rightarrow \Phi _\Sigma (0)Leb_{{{\mathbb {R}}}^2}(V)$, for any compact neighborhood $V\in {{\mathbb {R}}}^2$ with $Leb_{{{\mathbb {R}}}^2}(\partial V)=0$. A uniform LLT for $q_{\rho }$ can be obtained by, for instance, a straightforward adaptation of the argument used in [27, Proof of Theorem 2.7].

It is known that for every $\rho >0$, $\kappa _\rho$ is aperiodic, i.e., there exists no non-trivial solution to the equation $e^{it\kappa _\rho }g\circ T_\rho =g$. The aperiodicity of $\kappa _\rho$ has been used in [31] (in fact, in [30]) to provide LLT for fixed $\rho$. Given Proposition 6.3 and the aperiodicity of $\kappa _\rho$, the proof of Theorem 7.3 is classic, see [1] and for a variation of it that provides the uniformity in *N*, see, for instance, [28, First part of Proof of Theorem 2]. The proof below recalls the main elements needed to obtain the range of *n* in the statement.

### Theorem 7.3

Let $\delta _0=\delta ^{4/(3\nu -1)}$ be so that (34), (32) and Proposition 6.3 hold for all $|t|\in B_{\delta _0}(0)$. Since $\kappa _\rho$ is aperiodic, a known argument (see [Lemma 4.3 and Theorem 4.1] [1]) shows that $\Vert {\hat{R}}_{\rho }(t)^n\Vert _{{{\mathcal {B}}}}\le C_\rho (1-\gamma _{\rho })^n$, for all $|t|\ge \delta _0$. It follows that $|{{\mathbb {E}}}_\mu ({\hat{R}}_{\rho }(t)^n 1)|\le \Vert {\hat{R}}_{\rho }(t)^n \Vert _{{{\mathcal {B}}}}\le C_\rho \,(1-\gamma _{\rho })^n$ for every $|t| \in (\delta _0,\pi )$. Thus, using that $v\in C^{p_0}\subset {{\mathcal {B}}}$,

42 $$\begin{aligned} \int _{{{\mathcal {M}}}}&v1_{\{\kappa _{n,\rho }=N\}} w\circ T_\rho ^n\, d\mu =\frac{1}{4\pi ^2}\int _{ [-\pi ,\pi ]^2} e^{-itN} \int _{{{\mathcal {M}}}}{\hat{R}}_{\rho }(t)^n v \, w \, d\mu \, dt \nonumber \\&=\frac{1}{4\pi ^2}\int _{ [-\delta _0,\delta _0]^2} e^{-itN} \int _{{{\mathcal {M}}}}{\hat{R}}_{\rho }(t)^n v \, w \, d\mu \, dt +O\left( C_\rho \,(1-\gamma _{\rho })^n\right) \nonumber \\&=\frac{1}{4\pi ^2}\int _{ [-\delta _0,\delta _0]^2} e^{-itN} \lambda _\rho \left( t\right) ^n\int _{{{\mathcal {M}}}} \Pi _{\rho }(t)v \, w \, d\mu \, dt +O\left( C_\rho \,(1-\gamma _{\rho })^n+{\hat{C}}_\rho \,(1-{\hat{\gamma }}_{\rho })^n\right) \nonumber \\&=\frac{1}{4\pi ^2}I(\rho ,t)+O\left( C_\rho \,(1-\gamma _{\rho })^n\right) . \end{aligned}$$

Recall that $w\in L^a$, $a>1$ and set $b=a/(a-1)$. Using the Hölder inequality,

$$\begin{aligned} I(\rho ,t)= & {} \int _{ [-\delta ,\delta ]^2} e^{-itN} \lambda _\rho \left( t\right) ^n\, dt\, \int _{{{\mathcal {M}}}} v \, d\mu \,\int _{{{\mathcal {M}}}} w \, d\mu \\{} & {} +\int _{ [-\delta ,\delta ]^2} e^{-itN} \lambda _\rho \left( t\right) ^n\int _{{{\mathcal {M}}}} (\Pi _{\rho }(t)-\Pi _{\rho }(0))v \, w \, d\mu \, dt \\= & {} \int _{ [-\delta ,\delta ]^2} e^{-itN} \lambda _\rho \left( t\right) ^n\, dt\, \int _{{{\mathcal {M}}}} v \, d\mu \,\int _{{{\mathcal {M}}}} w \, d\mu \\{} & {} + O\left( \Vert w\Vert _{L^a(\mu )}\int _{ [-\delta ,\delta ]^2} \left| \lambda _\rho \left( t\right) ^n\right| \left( \int _{{{\mathcal {M}}}} |(\Pi _{\rho }(t)-\Pi _{\rho }(0))v|^b\, d\mu \right) ^{1/b}\, dt\right) . \end{aligned}$$

Recall $v\in {{\mathcal {B}}}$. Using (35), (29) and Lemma 6.1 and proceeding as in equation (38),

$$\begin{aligned} \Big (\int _{{{\mathcal {M}}}} |(\Pi _{\rho }(t)-\Pi _{\rho }(0))v|^b\, d\mu \Big )^{1/b} \le C \rho ^{-\nu }\gamma _{\rho }^{-2} |t|^{\nu }\le C \rho ^{-2}|t|^\varepsilon , \end{aligned}$$

for some uniform *C* and some $\varepsilon >0$. In the last inequality we have used that $|t|<\delta _0$. Thus,

$$\begin{aligned} I(\rho ,t)&=\int _{ [-\delta _0,\delta _0]^2} e^{-itN} \lambda _\rho \left( t\right) ^n\, dt\, \int _{{{\mathcal {M}}}} v \, d\mu \, \int _{{{\mathcal {M}}}} w \, d\mu \\ {}&\quad + O\left( \Vert w\Vert _{L^a(\mu )}\rho ^{-2} \int _{ [-\delta _0,\delta _0]^2} |t|^{\varepsilon } \left| \lambda _\rho \left( t\right) ^n\right| \, dt\right) . \end{aligned}$$

With a change of variables,

43 $$\begin{aligned} I(\rho ,t)&=\frac{1}{(b_{n,\rho })^2}\int _{ [-\delta _0 b_{n,\rho },\delta _0 b_{n,\rho }]^2} e^{-iu\frac{N}{b_{n,\rho }}} \lambda _{\rho } \left( \frac{u}{b_{n,\rho }}\right) ^n\, du\, \int _{{{\mathcal {M}}}} v \, d\mu \,\int _{{{\mathcal {M}}}} w \, d\mu \nonumber \\&\qquad + O\left( \Vert w\Vert _{L^a(\mu )} \frac{\rho ^{-2}}{(b_{n,\rho })^3}\int _{ [-\delta _0 b_{n,\rho },\delta _0 b_{n,\rho }]^2} |u|^{\varepsilon } \left| \lambda _{\rho } \left( \frac{u}{b_{n,\rho }}\right) ^n\right| du\right) . \end{aligned}$$

Given the range of *n* in the statement, we use (41) to obtain

$$\begin{aligned} \lim _{\rho \rightarrow 0}\left| 4\pi ^2\,\int _{ [-\delta _0 b_{n,\rho },\delta _0 b_{n,\rho }]^2} e^{-iu\frac{N}{b_{n,\rho }}} \lambda _{\rho } \left( \frac{u}{b_{n,\rho }}\right) ^n\, du-\Phi _\Sigma \left( \frac{N}{b_{n,\rho }}\right) \right| =0. \end{aligned}$$

To deal with the big *O* term in (43), we use that by (41) there exists a uniform constant *C* so that

$$\begin{aligned} \frac{\rho ^{-2}}{(b_{n,\rho })^3}\int _{ [-\delta _0 b_{n,\rho },\delta _0 b_{n,\rho }]^2} |u|^{\varepsilon } \left| \lambda _{\rho } \left( \frac{u}{b_{n,\rho }}\right) ^n\right| \,du\le \frac{\rho ^{-2}}{(b_{n,\rho })^{2+\varepsilon }}\int _{ [-\delta _0 b_{n,\rho },\delta _0 b_{n,\rho }]^2} |u|^{\varepsilon } e^{-C|u|^2}\, du. \end{aligned}$$

Since $M(\rho )=o(\log n)$, we have $n\gg \exp \left( C\rho ^{-2}\right)$. Thus, $\frac{\rho ^{-2}}{(b_{n,\rho })^{2+\varepsilon }}\ll \frac{\log n}{(b_{n,\rho })^{2+\varepsilon }}=o\left( \frac{1}{(b_{n,\rho })^2}\right)$ as $\rho \rightarrow 0$. Putting these together and using (43),

$$\begin{aligned} \lim _{\rho \rightarrow 0}\left| 4\pi ^2\, I(\rho ,t) -\Phi _\Sigma \left( \frac{N}{b_{n,\rho }}\right) \, \int _{{{\mathcal {M}}}} v \, d\mu \,\int _{{{\mathcal {M}}}} w \, d\mu \,\right| =0. \end{aligned}$$

This together with (42) gives that as $\rho \rightarrow 0$,

$$\begin{aligned}&\left| \int _{{{\mathcal {M}}}} v1_{\{\kappa _{n,\rho }=N\}} w\circ T_\rho ^n\, d\mu -\frac{1}{(b_{n,\rho })^2} \Phi _\Sigma \left( \frac{N}{b_{n,\rho }}\right) \,\int _{{{\mathcal {M}}}} v \, d\mu \,\int _{{{\mathcal {M}}}} w \, d\mu \,\right| \\&\qquad =O\left( (b_{n,\rho })^2\ C_\rho \,(1-\gamma _{\rho })^n \right) =o(1), \end{aligned}$$

where in the last equation we used that $M(\rho )=o(\log n)$. This concludes the proof. $\square$

It is known that the local limit theorem for $\kappa _\rho$ and the billiard map $T_{\rho }$ (with $\rho$ fixed) implies mixing for the planar Lorentz map ${\hat{T}}_{\rho }$ (again $\rho$ fixed), see [28]. In fact, sharp error rates in local limit theorems and mixing are also known, see [28] for the finite horizon case and [29] for the infinite horizon case.

We recall from Sect. 1 that the Lorentz map ${\hat{T}}_{\rho }$ defined on ${\widehat{{{\mathcal {M}}}}}={{\mathcal {M}}}\times {{\mathbb {Z}}}^2$ is given by ${\hat{T}}_{\rho }(\theta ,\phi ,\ell ) = ( T_{\rho }(\theta ,\phi ), \ell +\kappa _\rho (\theta ,\phi ))$ for $(\theta ,\phi ) \in {{\mathcal {M}}},\ \ell \in {{\mathbb {Z}}}^2$. Let ${\hat{\mu }}=\mu \times \text{ Leb}_{{{\mathbb {Z}}}^2}$, where $\text{ Leb}_{{{\mathbb {Z}}}^2}$ is the counting measure on ${{\mathbb {Z}}}^2$. An immediate consequence of Theorem 7.3 is

### Corollary 7.5

Assume the assumptions and notation of Theorem 7.3. Let $\rho \rightarrow 0$ and simultaneously $n\rightarrow \infty$ in such a way that $M(\rho )=o(\log n)$. Then

$$\begin{aligned} \lim _{\rho \rightarrow 0}\left| (b_{n,\rho })^2\int _{{\widehat{{{\mathcal {M}}}}}}v\, w\circ {\hat{T}}_{\rho }\, d{\hat{\mu }}-\int _{{\widehat{{{\mathcal {M}}}}}} v \, d{\hat{\mu }}\,\int _{{\widehat{{{\mathcal {M}}}}}} w \, d{\hat{\mu }}\,\right| =0. \end{aligned}$$

### Remark 7.6

The class of functions in Corollary 7.5 is rather restrictive as the functions *v*, *w* are supported on the cell ${{\mathcal {M}}}$. Given the work [28] (see also [29, Sect. 6]), it is very plausible that the present mixing result can be generalized to a suitable class of dynamically Hölder functions supported on the whole of ${\widehat{{{\mathcal {M}}}}}$. Since the involved argument is rather delicate and not a main concern of the present work, we omit this.

## Figures and Tables

**Fig. 5:**
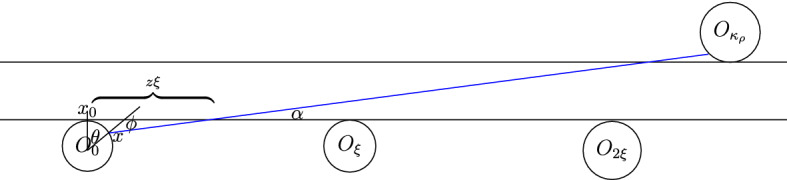
A corridor and coordinates $(\alpha ,\theta )$

**Fig. 6:**
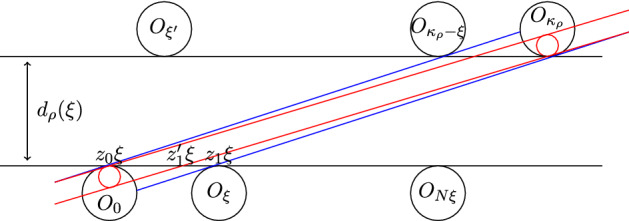
$[z_0,z_1]$ given by two tangent lines for $2\rho > \frac{d_{\rho }(\xi )}{N}$ (blue) or $2\rho < \frac{d_{\rho }(\xi )}{N}$ (red)

**Fig. 7:**
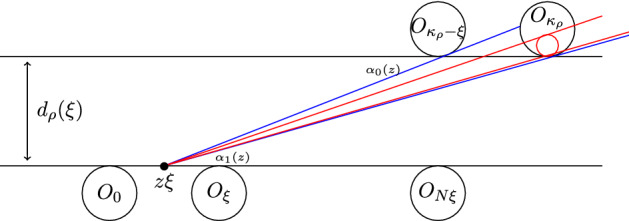
The parameter interval $[\alpha _0(z),\alpha _1(z)]$ given by angles between two tangent lines

## Data Availability

Data sharing not applicable to this article as no datasets were generated or analysed during the current study.

## A Estimates on corridors

### A.1 Estimating ${{\mathbb {P}}}(\kappa _\rho = \xi '+N\xi )$

Given a corridor associated to $\xi$, there a neighborhood $U_0$ of $x_0 = x_0(\xi )$ in $\partial O_0 \times [-\frac{\pi }{2},\frac{\pi }{2}]$ of initial conditions *x* such that the next collision occurs at a scatterer on the opposite side of the corridor. For this situation, [31] use the coordinates $(\alpha , z)$, where $\alpha$ is the angle the trajectory of some $x \in \partial O_0$ makes with the tangent line at $x_0$, and the intersection point is $y = x_0+z \xi$, see Fig. 5.

#### Lemma A.1

In coordinates $(z,\alpha )$ the volume form in a neighborhood of $x_0 = x_0(\xi )$ is

$$\begin{aligned} \frac{|\xi |}{4\pi \rho } \sin \alpha \, d\alpha \, dz = \frac{1}{4\pi } \cos \phi \, d\theta \, d\phi . \end{aligned}$$

#### Proof

The part $\sin \alpha \, d\alpha \, dz$ can be understood because the Liouville measure of the billiard flow projects to a form $\cos \varphi \, d\varphi \, dr$ for any transversal section parametrised by arc-length *r* and with $\varphi$ the angle of the trajectory to the normal vector at the collision point. When this section is the line $y = x_0 + x \xi$, we have $\alpha = \frac{\pi }{2}-\varphi$, so $\cos \varphi = \sin \alpha$. But to get the correct normalizing constant, we give a more extensive argument. From Fig. 5 we have

44 $$\begin{aligned} \frac{\pi }{2} = \theta + \alpha + \phi , \qquad \tan \alpha = \frac{\rho (1-\cos \theta )}{z|\xi | - \rho \sin \theta }. \end{aligned}$$

After making $\alpha$ and *z* subject of these equations, we see that the change of coordinates involved is

$$\begin{aligned} (\alpha , z) = F(\theta ,\phi ) = \left( \frac{\pi }{2} - \theta - \phi , \frac{\rho }{|\xi |} \left( \frac{1-\cos \theta }{\tan (\frac{\pi }{2}- \theta -\phi )} + \sin \theta \right) \right) . \end{aligned}$$

The Jacobian determinant is

$$\begin{aligned} |\det (dF)| = \left| \det \begin{pmatrix} -1 &{} -1 \\ \frac{\partial F_2}{\partial \theta } &{} \frac{\partial F_2}{\partial \phi } \end{pmatrix} \right| = \left| \frac{\partial F_2}{\partial \theta } - \frac{\partial F_2}{\partial \phi } \right| = \frac{\rho }{|\xi |} \left( \frac{\cos \theta }{\tan (\frac{\pi }{2}-\theta -\phi )} + \cos \theta \right) . \end{aligned}$$

Thus, using (44) and some trigonometric formulas,

$$\begin{aligned} \frac{|\xi |}{4\pi \rho } \sin \alpha \, d\alpha \, dz= & {} \frac{|\xi | \sin \alpha }{4\pi \rho } \frac{\rho }{|\xi |} \left( \frac{\sin \theta }{\tan (\frac{\pi }{2} - \theta -\phi )} + \cos \theta \right) \, d\theta \, d\phi \\= & {} \frac{1}{4\pi } (\cos \alpha \sin \theta + \sin \alpha \cos \theta ) \, d\theta \, d\phi \\= & {} \frac{1}{4\pi } \sin (\alpha + \theta ) \, d\theta \, d\phi = \frac{1}{4\pi }\cos (\phi ) \, d\theta \, d\phi , \end{aligned}$$

as claimed. $$\square $$

The following is [31, Proposition 6] in more detail:

#### Lemma A.2

Suppose that the scatterers have radius $\rho > 0$ and the width of the corridor given by $\xi$ is $d_\rho (\xi )$. Then

$$\begin{aligned}{} & {} \mu (\{ x \in \partial O_0 \times [-\frac{\pi }{2}, \frac{\pi }{2}]: \kappa _\rho (x)\\{} & {} \quad = N |\xi | + \xi ' \}) = \frac{1}{4\pi N |\xi | \rho } \min \{ 4\rho ^2, d_{\rho }(\xi )^2 N^{-2}\} (1+{{\mathcal {O}}}(N^{-1})), \end{aligned}$$

where $\xi '$ as in Remark 2.2 is the integer vector on the boundary of the corridor opposite to the $\xi$-boundary.

#### Proof

We take the region in $(z,\alpha )$-coordinates where $\kappa _\rho = N \xi + \xi '$. In the *z*-direction this is an interval $[z_0, z_1]$, where for $z = z_0$, there is only one line connecting $O_0$ and $O_{\kappa _\rho }$, namely the common tangent line of $O_0$ and $O_{\kappa _\rho -\xi }$. For $z = z_1$ there is also is only one line, namely the common tangent line of $O_\xi$ and $O_{\kappa _\rho }$, see Fig. 6. These two lines are obtained from each other by translation over one unit $\xi$, so $z_1\xi - z_0\xi = |\xi |$. However, if $\rho$ is small compared to *N*, these two tangent lines are the common tangent lines at the upper sides of $O_0$ and $O_{\kappa _\rho }$ and at the lower sides of $O_0$ and $O_{\kappa _\rho }$. In this case

45 $$\begin{aligned} |z_1\xi -z_0\xi | = \frac{2\rho }{\sin \alpha } = \frac{2 \rho (N|\xi |+|\xi '|)}{d_{\rho }(\xi )+2\rho } + {{\mathcal {O}}}\left( \frac{\rho }{d_{\rho }(\xi )+2\rho }\right) . \end{aligned}$$

This also shows that the transition between the two cases is when $2\rho = \frac{d_\rho (\xi )}{N}$.

For each $z \in [z_0, z_1]$, the range of possible values of $\alpha$ is again bounded by the $\alpha$’s obtained at the tangent lines to $O_{\kappa _\rho -\xi }$ and $O_{\kappa _\rho }$. Therefore, see Fig. 7,

$$\begin{aligned} \alpha \in [\alpha _0(z),\alpha _1(z)]:= \left[ \arctan \left( \frac{d_\rho (\xi )}{N|\xi |+|\xi '|-z}\right) \, \ \arctan \left( \frac{d_\rho (\xi )}{N|\xi | - |\xi | + |\xi '| - z}\right) \right] . \end{aligned}$$

Since $|\xi '| \le |\xi |$ (see Remark 2.2) and $z \le |\xi |$ as well, each $\alpha$ in this interval satisfies $\alpha = \frac{d_\rho (\xi )}{N\, |\xi |}(1 + {{\mathcal {O}}}(N^{-1}))$ and

46 $$\begin{aligned} \alpha _1(z) - \alpha _0(z) = \frac{d_\rho (\xi )}{N^2|\xi |} (1+ {{\mathcal {O}}}(N^{-1})). \end{aligned}$$

Integrating the density given in Lemma A.1 for the case $2\rho \ge \frac{d_\rho (\xi )}{N}$ (so $|z_1-z_0| = |\xi |$) and using $|z_1-z_0|=|\xi |$ and the approximation $\cos \alpha _0 - \cos \alpha _1 \sim \frac{1}{2} (\alpha _1+\alpha _0) (\alpha _1-\alpha _0)$ gives:

$$\begin{aligned} \int _{z_0}^{z_1} \int _{\alpha _0(z)}^{\alpha _1(z)} \frac{|\xi |}{4\pi \rho } \sin \alpha \, d\alpha \, dz= & {} \frac{|\xi |}{4\pi \rho } \int _{z_0}^{z_1} \cos (\alpha _0(z)) - \cos (\alpha _1(z)) \, dz \\= & {} \frac{|\xi |}{4\pi \rho } \frac{d_{\rho }(\xi ) }{ N |\xi | } \frac{d_{\rho }(\xi ) }{ N^2 |\xi | } ( 1+{{\mathcal {O}}}(N^{-1}) ) \\= & {} \frac{1}{4\pi N \rho } \, \frac{d_\rho (\xi )^2}{|\xi |N^2} \left( 1 + {{\mathcal {O}}}(N^{-1}) \right) . \end{aligned}$$

Now for the case $2\rho < \frac{d_\rho (\xi )}{N}$, see Fig. 7 with small version of $O_{\kappa _\rho }$, we have

$$\begin{aligned}{} & {} \alpha \in [\alpha _0(z), \alpha _1(z)]\\ {}{} & {} \quad := \left[ \arctan \left( \frac{d_\rho (\xi )}{N|\xi |+Q-z-2\rho \sin \alpha }\right) \, \ \arctan \left( \frac{d_\rho (\xi )+2\rho \cos \alpha _1(z)}{N|\xi |+Q - z-2\rho \sin \alpha }\right) \right] , \end{aligned}$$

so still $\alpha = \frac{d_\rho (\xi )}{N\, |\xi |} + {{\mathcal {O}}}(N^{-2})$ and $\alpha _1(z) - \alpha _0(z) = \frac{2\rho }{N|\xi |} (1+ {{\mathcal {O}}}(N^{-1})$.

Integrating as before gives, using (45) and the fact that $d_{\rho }(\xi )+2\rho =|\xi |^{-1}$ from Lemma 2.1:

$$\begin{aligned} \int _{z_0}^{z_1} \int _{\alpha _0(z)}^{\alpha _1(z)} \frac{|\xi |}{4\pi \rho } \sin \alpha \, d\alpha \, dz= & {} \frac{|\xi |}{4\pi \rho } \int _{z_0}^{z_1} \cos (\alpha _0(z)) - \cos (\alpha _1(z)) \, dz \\= & {} \frac{|\xi |}{4\pi \rho }\, \frac{2 \rho N }{ d_{\rho }(\xi )+2\rho }\, \frac{ d_{\rho }(\xi ) }{ N |\xi | } \frac{ 2\rho }{ N |\xi | } ( 1+{{\mathcal {O}}}(N^{-1}) ) \\= & {} \frac{ 4\rho ^2 }{4\pi |\xi | N \rho } \, \left( 1 + {{\mathcal {O}}}(N^{-1}) \right) \end{aligned}$$

as required. $$\square $$

### A.2 Corridors sums

Let $\varphi$ be Euler’s totient function, i.e., the number of integers $1 \le q \le p$ coprime with *p*. The following lemma is classical number theory, but we couldn’t locate a proof of the full statement.

#### Lemma A.3

For every $a > -2$, we have

$$\begin{aligned} \sum _{n=1}^N n^a \varphi (n) = \frac{N^{a+2}}{a+2} \, \frac{1}{\zeta (2)} (1+o(1)), \end{aligned}$$

where $\zeta$ is the Riemann $\zeta$-function, so $\zeta (2) = \frac{\pi ^2}{6}$.

#### Proof

Let $\mu$ be the Möbius function. A standard equality is $\varphi (n) = \sum _{d|n} \mu (d) \frac{n}{d}$. Therefore

$$\begin{aligned} \sum _{n=1}^N n^a \varphi (n)= & {} \sum _{n=1}^N \sum _{d|n} n^a \mu (d) \frac{n}{d} = \sum _{n=1}^N \sum _{d|n} d^a \mu (d) \left( \frac{n}{d}\right) ^{a+1} \\= & {} \sum _{d=1}^N \sum _{m=1}^{\frac{N}{d}} d^a \mu (d) m^{a+1} = \sum _{d=1}^N d^a \mu (d) \frac{1}{a+2} \left( \frac{N}{d}\right) ^{a+2} (1+o(1)) \\= & {} \frac{N^{a+2}}{a+2} \, \sum _{d=1}^N \frac{\mu (d)}{d^2} (1+o(1)) = \frac{N^{a+2}}{a+2} \, \frac{1}{\zeta (2)} (1+o(1)), \end{aligned}$$

where we used the Dirichlet series identity $\sum _{d=1}^\infty \frac{\mu (d)}{d^s} = \frac{1}{\zeta (s)}$ for $s=2$.

As an aside, there are asymptotic formulas for $s > 2$

47 $$\begin{aligned} \sum _{p \ge 1} \frac{\varphi (p)}{p^s} = \frac{\zeta (s-1)}{\zeta (s)} \quad \text {and} \quad \sum _{p=1}^N \frac{\varphi (p)}{p} = \frac{N}{\zeta (2)} + {{\mathcal {O}}}((\log N)^{\frac{2}{3}} (\log \log N)^{\frac{4}{3}}),\qquad \end{aligned}$$

see [19, Theorem 288]. $$\square $$

In the course of this paper we denote, for a fixed value of $\rho$, the set of corridors that are “visible” from the origin by ${\Psi }$. As described in Lemma 2.1, these can be characterized by pairs $(\xi ,\xi ')\in {{\mathbb {Z}}}^2 \times {{\mathbb {Z}}}^2$ where $\xi = (p,q)$, $\gcd (p,q) = 1$ and $|\xi | \le (2\rho )^{-1}$, while $\xi '$ may denote either the first or the second convergent preceding $\xi$ in the continued fraction expansion of *p*/*q*, see Remark 2.2. Sums of the type in the following lemma are used throughout the paper.

#### Lemma A.4

We have

$$\begin{aligned} \sum _{(\xi ,\xi ') \in {\Psi }} |\xi |^a {\left\{ \begin{array}{ll} \sim \frac{2}{a+2} \frac{2\pi }{\zeta (2)}(2\rho )^{-(a+2)} &{} \text { if } a > -2;\\ \asymp |\log \rho | &{} \text { if } a = -2;\\ \le -\frac{4\pi }{a+2} &{} \text { if } a < -2. \end{array}\right. } \end{aligned}$$

#### Proof

Using the two coordinate axes and their bisectrices, we divide the plane into eight sectors and for each sector, we sum the scatterers in ${{\mathcal {S}}}$. Circular sections of radius *R* have asymptotically $\frac{\pi }{4}$ as many points as triangular sectors with base *R*. Also, every corridor direction in this sector comes with two corridors $(\xi ,\xi ')$ and $(\xi ,\xi '')$. By Lemma A.3, their sum is, for $a > -2$,

$$\begin{aligned} \sum _{(\xi ,\xi ') \in {\Psi }} |\xi |^a\sim & {} \frac{16\pi }{4} \sum _{0 \le q \le p \le (2\rho )^{-1}} |\xi |^a = 4\pi \sum _{1 \le p \le (2\rho )^{-1}} \phi (p) p^a \sim \frac{4\pi }{2+a} \frac{1}{\zeta (2)} (2\rho )^{-(2+a)}. \end{aligned}$$

If $a = -2$, then a similar computation gives $\asymp |\log \rho |$, and for $a < -2$, the series is summable: $4\pi \sum _{1 \le p \le (2\rho )^{-1}} \phi (p) p^a \le 4\pi \int _1^\infty x^a \, dx = -\frac{4\pi }{2+a}$. $\square$

#### Lemma A.5

For $p \in [1,2)$, the *p*-norm of the displacement function satisfies

$$\begin{aligned} \Vert \kappa _\rho \Vert _{L^p} \ll (p(2-p))^{-1/p} \ \rho ^{-1}. \end{aligned}$$

#### Proof

Take $p \in [1,2)$. We estimate over all $\xi$-corridors similarly as in Lemma A.4:

$$\begin{aligned} \int |\kappa _\rho |^p \, d\mu\ll & {} 2\sum _{|\xi | \le (2\rho )^{-1} } \sum _{N \ge 1} |\xi |^p N^p \frac{1}{4\pi |\xi | N \rho } \min \{ 4\rho ^2, d_{\rho }(\xi )^2 N^{-2}\} \\\le & {} \frac{1}{2\pi \rho } \sum _{|\xi | \le (2\rho )^{-1}} |\xi |^{p-1} \left( \sum _{N=1}^{\lfloor d_{\rho }(\xi )/(2\rho ) \rfloor } 4\rho ^2 N^{p-1} + \sum _{N=\lfloor d_{\rho }(\xi )/(2\rho )\rfloor }^\infty d_{\rho }(\xi )^2 N^{p-3}\right) \\\le & {} \frac{1}{2\pi \rho } \left( \frac{1}{p} (2\rho )^{2-p} + \frac{1}{2-p} (2\rho )^{2-p}\right) \sum _{|\xi | \le (2\rho )^{-1}} |\xi |^{-1} \\\sim & {} \frac{2}{\zeta (2)} \left( \frac{1}{p} + \frac{1}{2-p} \right) \, ( 2\rho )^{-p}. \end{aligned}$$

Taking the *p*-th root gives the result. $\square$

#### Lemma A.6

Let $W \in {{\mathcal {W}}}^s$ be a stable leaf, and let ${\Psi }_W$ stand for all lattice points $\xi = (p,q) \in {\Psi }$ that can be reached from $O_0$ with coordinates in *W*. Then for every $a \in (\frac{1}{2},1)$,

$$\begin{aligned} \sum _{(\xi ,\xi ') \in {\Psi }_W} |\xi |^{-a} \ll \rho ^{a-2} |W| + \rho ^{a-1} \log (1/\rho ) + \rho ^{a-1}|W|^{-1}. \end{aligned}$$

#### Proof

There is an arc ${\tilde{W}} \in {{\mathbb {S}}}^1$ of length $|{\tilde{W}}| \ll |W|$ such that every lattice point that can be reached from $O_0$ with coordinates in *W* has its polar angle in ${\tilde{W}}$. Due to the symmetries in the ${{\mathbb {Z}}}^2$, it suffices to study ${\tilde{W}} \subset [0, \pi /2]$, so the lattice point $\xi = (p,q)$ in this sector satisfy $0 \le q \le p$ and $\tan ({\tilde{W}}) \subset [0,1]$. In fact, we will start by assuming that $\tan ({\tilde{W}}) \in [\frac{1}{10}, \frac{9}{10}]$.

Because $p^2+q^2 \ge 2pq$ for all $(p,q) = \xi$, we have $\sum _{(\xi ,\xi ') \in {\Psi }_W} |\xi |^{-a} \ll 2^{-a/2} \sum _{(\xi ,\xi ') \in {\Psi }} \frac{1}{(pq)^{a/2}} 1_{{\tilde{W}}}(\frac{q}{p})$. We will apply an estimate from [33, Theorem 2.2], which, in our terminology, reduces to

48 $$\begin{aligned} \sum _{(\xi ,\xi ') \in {\Psi }} \frac{1}{(pq)^{a/2}}\, \psi \left( \frac{p}{q}\right)= & {} C_a\, \rho ^{a-2} \int \psi (x) \, dx + O(\rho ^{1-a} \log (1/\rho )) \nonumber \\{} & {} +\ O\left( \sum _{\ell \ne 0} c_\psi (\ell ) \sum _{{\mathop {d | \ell }\limits ^{d \le (2\rho )^{-1}}}} d^{1-a} \sum _{k \le (2\rho d)^{-1}} \frac{\mu (k)}{k^a} \right) ,\qquad \end{aligned}$$

where $C_a$ is a constant depending only on *a*, and $c_{\psi }(\ell )$ is the $\ell$-th Fourier coefficient of $x \mapsto \psi (x) x^{-a}$.

If $\psi = 1_{{\tilde{W}}}$, then these Fourier coefficients are not summable, so we first smoothen $1_{{\tilde{W}}}$ to a function $\psi$ with $\mathop {\text{ supp }}(\psi )$ concentric to ${\tilde{W}}$ and $|\mathop {\text{ supp }}(\psi )| = |{\tilde{W}}|= 3|W|$. On ${\tilde{W}}$ itself, $\psi \equiv 1$ and on the two interval components $\psi$ is a translated copy of the function $f_W:[-\frac{|W|}{2}, \frac{|W|}{2}] \rightarrow {{\mathbb {R}}}$ defined by

$$\begin{aligned} f_W(x) = \frac{1}{2}-\frac{1}{2\pi } \sin \frac{2\pi x}{|W|} + \frac{x}{|W|}. \end{aligned}$$

Then $\int \psi \, dx = 2|W|$ and integrating by parts twice gives an estimate of the Fourier coefficients of $x \mapsto \psi (x) x^{-a}$.

$$\begin{aligned} |c_{\psi }(\ell )| \ll \left| \int \frac{(\psi (x)x^{-a})''}{(2\pi \ell )^2} \, e^{2\pi i \ell x} \, dx \right| \ll \frac{1}{|W|\ell ^2} \end{aligned}$$

because $\mathop {\text{ supp }}(\psi )$ is bounded away from $\{ 0,1\}$ (so $x^{-a}$ doesn’t blow up) and $(\psi (x) x^{-a})'' = 0$ outside $\mathop {\text{ supp }}(\psi )$.

The Dirichlet series of the Möbius function can be estimated as $\left| \sum _{k=1}^{\lfloor 1/(2\rho d)\rfloor } \mu (k) k^{-a}\right| \le (2\rho d)^{1-a}$. We use this and the fact that $\ell \in {{\mathbb {N}}}$ has $O(\ell ^{1/2})$ divisors to estimate the last big *O*-term in (48).

$$\begin{aligned} \sum _{\ell \in {{\mathbb {N}}}} |c_\psi (\ell )| \sum _{{\mathop {d | \ell }\limits ^{d \le (2\rho )^{-1}}}} d^{1-a} \sum _{k \le (2\rho d)^{-1}} \frac{\mu (k)}{k^a}\ll & {} \frac{(2\rho )^{a-1}}{|W|} \sum _{\ell \in {{\mathbb {N}}}} |c_\psi (\ell )| \sum _{{\mathop {d | \ell }\limits ^{d \le (2\rho )^{-1}}}} 1 \\\ll & {} \frac{(2\rho )^{1-a}}{|W|} \sum _{\ell \ne 0} |\ell |^{-\frac{3}{2}} \le \frac{(2\rho )^{a-1}}{|W|}. \end{aligned}$$

Hence (48) becomes

$$\begin{aligned} \sum _{(\xi ,\xi ') \in {\Psi }} \frac{1}{(pq)^{a/2}} 1_{{\tilde{W}}}\left( \frac{q}{p}\right)\le & {} \sum _{(\xi ,\xi ') \in {\Psi }} \frac{1}{(pq)^{a/2}}\, \psi \left( \frac{q}{p}\right) \\\ll & {} \rho ^{a-2} |W| + \rho ^{a-1} \log (1/\rho ) + \rho ^{a-1} |W|^{-1}, \end{aligned}$$

as required.

It remains to consider the cases that $\tan ({\tilde{W}}) \not \subset [\frac{1}{10}, \frac{9}{10}]$. Suppose instead that $\tan ({\tilde{W}}) \subset (0, \frac{1}{10}]$ (we ignore $\xi = (0,1)$ and $\xi = (1,0)$). In this case, we give an injection between the lattice points in the ${\tilde{W}}$-sector with coprime coordinates to the set of lattice points (with coprime coordinates and comparable norm) in a sector of comparable width, but near polar angle $\frac{1}{2}$. Indeed, set ${{\mathbb {Q}}}_{cp} = \{ q/p: 0 \ne p, q \in {{\mathbb {Z}}}, \gcd (p,q) = 1\} \cup \{0\}$ and ${{\mathbb {Z}}}_{cp}:= \{ (p, q) \in {{\mathbb {Z}}}^2: \gcd (p,q) = 1\}$, and define the Calkin-Wilf map $f:{{\mathbb {Q}}}_{cp} \rightarrow {{\mathbb {Q}}}_{cp}$ as well as $g:{{\mathbb {Z}}}_{cp} \rightarrow {{\mathbb {Z}}}_{cp}$ by

$$\begin{aligned} f: x \mapsto \frac{1}{1-x-2\lfloor x \rfloor },\qquad \qquad g: (p,q) \mapsto ( p-q+2p \lfloor q/p \rfloor \, \ p). \end{aligned}$$

The *f*-orbit of 0 enumerates all non-negative lowest-term rationals, see [8], and *g* is the same map expressed on the collection of lattice points. Since $f^2( (0,\frac{1}{10}] ) \subset (\frac{1}{2},\frac{10}{21}]$ and $|g(\xi )| \le 4|\xi |$, the second iterate $g^2$ provides the required injection. In case $\tan (\tilde{W}) \subset [\frac{9}{10},1)$ we use $g^3$. $\square$

## B Distortion properties

Throughout, a uniform constant is a constant that is independent of $\rho$.

Let us recall some terminology and notations from [10, Chapter 4]. Unstable curves generate dispersing wavefronts, which are evolved by the free flight, and then leave traces of unstable curves on the scatterer at the next collision. For wavefronts it is convenient to use the Jacobi coordinates $(d\xi ,d\omega )$, and an important quantity^5^$\Omega =\frac{d\omega }{d\xi }$, the curvature of the wavefront. Let $\Omega ^-$ and $\Omega ^+$ denote its value immediately before and after a particular collision, respectively.

On the scatterer, the traditional coordinates are $(r,\phi )$ yet, we prefer to use the $\rho$-independent $(\theta ,\phi )$ and take advantage of

$$\begin{aligned} \frac{d}{d\theta }=(2\pi \rho ) \frac{d}{dr}. \end{aligned}$$

First we relate $\Omega ^-$ to the slope of the unstable curve: $\frac{1}{2\pi } \frac{d\phi }{d\theta } =\rho \Omega ^- \cos \phi +1$. Differentiating with respect to $\theta$ gives

49 $$\begin{aligned} \frac{1}{2\pi } \frac{d^2\phi }{d\theta ^2}=\frac{d\Omega ^-}{d\theta } \rho \cos \phi -\rho \Omega ^- \sin \phi \frac{d\phi }{d\theta }. \end{aligned}$$

### Lemma B.1

There exists a uniform constant $C>0$ such that for any $C^2$ smooth unstable curve *W* there exists $n_W$ such that for $n\ge n_W$ on all components of $T_{\rho }^nW$ we have

50 $$\begin{aligned} \left| \frac{d^2\phi }{d\theta ^2}\right| \le C\rho . \end{aligned}$$

Thus we may restrict to the class of *regular* unstable curves for which (50) holds. Also, this shows that as $\rho \rightarrow 0$, the unstable curves limit in a $C^2$ sense to straight lines of slope $2\pi$.

### Proof

The properties of the free flight are not effected by shrinking the scatterers or using the $\theta$-coordinate. Thus

$$\begin{aligned} 0\le \Omega ^-\le (\tau _{min})^{-1} \end{aligned}$$

and, by (49), it is enough to show

$$\begin{aligned} \left| \frac{d\Omega ^-}{d\theta }\right| \le C \end{aligned}$$

to prove the lemma. Now $\frac{d\Omega ^-}{d\theta }=(2\pi \rho ) \frac{d\Omega ^-}{dr}$, and the evolution of $\frac{d\Omega ^-}{dr}$ is discussed in [10, section 4.6]. Following the notation there, introduce

$$\begin{aligned} {{\mathcal {E}}}_1=\frac{d\Omega }{d\xi };\qquad F_1=\frac{{{\mathcal {E}}}_1}{\Omega ^3} \end{aligned}$$

and use superscripts − and $+$ to denote pre- and post-collision values of these quantities, respectively. [10, Formula (4.37)] states

$$\begin{aligned} -F_1^+=\left( \frac{\Omega ^-}{\Omega ^+}\right) ^3 F_1^- +H_1, \end{aligned}$$

where

$$\begin{aligned} H_1=\frac{6\rho ^{-2}\sin \phi +6\rho ^{-1} \Omega ^- \cos \phi \sin \phi }{(2\rho ^{-1}+\Omega ^-\cos \phi )^3} \end{aligned}$$

and by the analysis of [10, page 81]:

- $F_1$ remains constant between collisions
- there exists a uniform constant $\Theta <1$ such that $\frac{\Omega ^-}{\Omega ^+}\le \Theta$,
- there exists a uniform constant $C_1>0$ such that $|H_1|\le C_1$. This remains valid for shrinking $\rho$ as the denominator scales with $\rho ^{-3}$ while the numerator scales with $\rho ^{-2}$.

Hence it follows that $|F_1(n+1)|\le \Theta ^3 |F_1(n)| +C$, where $F_1(n)$ is the value of $F_1$ between the *n*-th and the $(n+1)$st collision. This implies that there exists $C_2>0$ and $n_W$ (depending on the curve *W*) such that for any $n\ge n_W$ we have $|F_1(n)|\le C_2$.

Now $|{{\mathcal {E}}}_1^-|=|F_1^-|\cdot (\Omega ^-)^3\le C_3$ for some uniform $C_3>0$, and finally [10, Formula (4.24)] states

$$\begin{aligned} \frac{d\Omega ^-}{dr}={{\mathcal {E}}}_1^- \cos \phi - (\Omega ^-)^2\sin \phi , \end{aligned}$$

which thus implies that $\left| \frac{d\Omega ^-}{dr}\right| \le C_4$ for some uniform constant $C_4>0$. This bound completes the proof of the lemma. $\square$

It follows that regular unstable curves can be parametrised by the coordinate $\theta$, and for any smooth function $f:W\rightarrow {{\mathbb {R}}}$, $\frac{df}{d\theta }\asymp \frac{df}{dx}$, where *x* is (Euclidean) arc-length along the curve—$dx^2=d\theta ^2+d\phi ^2$ (not to be confused with the arc-length *r* along the scatterer).

Let us also recall that an unstable curve is homogeneous if it is regular and contained in one of the homogeneity strips ${{\mathbb {H}}}_k=\{(\theta ,\phi ): \frac{\pi }{2}-k^{-r_0}< \phi < \frac{\pi }{2}-(k+1)^{-r_0} \}$. For such curves, analogous to [10, Formula (5.13)], we have

51 $$\begin{aligned} |W|\le C \cos ^{\frac{r_0+1}{r_0}} \phi \end{aligned}$$

for some uniform constant $C>0$, where $\phi$ corresponds to any point of *W*. (This follows as the slope of the curve is uniformly bounded away from 0 and $\infty$.)

Distortion bounds are stated as follows. Let *W* be a homogeneous unstable curve, and assume that for some $N\ge 1$, $W_n=T_{\rho }^{-n}W$ is a homogeneous unstable curve for $n=0,1,\dots ,N$. For $x\in W$, let $x_n=T_{\rho }^{-n}x\in W_n$. Let $J_WT_{\rho }^{-n}(x)$ and $J_{W_n} T_{\rho }^{-1}(x_n)$ denote the respective Jacobians.

### Lemma B.2

Consider *W* and *N* as above and $y,z\in W$ arbitrary. There exists a uniform constant $C_d>0$ such that

$$\begin{aligned} |\log J_WT_{\rho }^{-N}(y)-\log J_WT_{\rho }^{-N}(z)|\le C_d|W|^{\frac{1}{r_0+1}}. \end{aligned}$$

### Proof

The lemma relies on the inequality

52 $$\begin{aligned} \left| \frac{d}{dx_n} \log J_{W_n} T_{\rho }^{-1}(x_n) \right| \le \frac{C}{\cos \phi _n} \end{aligned}$$

for some uniform $C>0$, cf. [10, Formula (5.8)].

Using this formula the argument in the proof of [10, Lemma 5.27] can be repeated literally:

53 $$\begin{aligned} |\log J_WT_{\rho }^{-N}(y)-\log J_WT_{\rho }^{-N}(z)|\le & {} \sum _{n=0}^{N-1} |\log J_{W_n}T_{\rho }^{-1}(y_n)-\log J_{W_n}T_{\rho }^{-1}(z_n)| \nonumber \\\le & {} \sum _{n=0}^{N-1} |W_n| \max \left| \frac{d}{dx_n} \log J_{W_n} T_{\rho }^{-1}(x_n) \right| \nonumber \\\le & {} C \sum _{n=0}^{N-1} \frac{|W_n|}{\cos \phi _n} \le C \sum _{n=0}^{N-1} |W_n|^{\frac{1}{r_0+1}}\le C |W|^{\frac{1}{r_0+1}},\nonumber \\ \end{aligned}$$

where we have used the chain rule, (52), and (51) and the uniform hyperbolicity.

It remains to prove (52). Here we essentially follow [10, pp. 106–107]. We have

$$\begin{aligned} \log J_{W_n} T_{\rho }^{-1}(x_n)= & {} \log \cos \phi _n + \frac{1}{2} \log \left( 4\pi ^2\rho ^2 +\left( \frac{d\phi _n}{d\theta _n}\right) ^2 \right) \\ {}{} & {} -\frac{1}{2} \log \left( 4\pi ^2\rho ^2 +\left( \frac{d\phi _{n+1}}{d\theta _{n+1}}\right) ^2 \right) \nonumber \\{} & {} - \log \left( 2\rho ^{-1} \tau _{n+1}+ \cos \phi _{n+1}(1+\tau _{n+1}\Omega ^-_{n+1}) \right) .\nonumber \end{aligned}$$

We consider the derivatives of these terms separately. As noted above, differentiation with respect to $\theta _n$ and $x_n$ can be interchanged. By Lemma B.1, the derivative of the second term w.r.t. $\theta _n$ is uniformly bounded. The same applies to the derivative of the third term with respect to $\theta _{n+1}$, while

$$\begin{aligned} \frac{dx_{n+1}}{dx_n}= J_{W_n} T_{\rho }^{-1}(x_n) \end{aligned}$$

is uniformly bounded from above. The first term gives the main contribution: as $\cos \phi _n$ is not bounded away from 0, the derivative of its logarithm is

$$\begin{aligned} \left| \frac{d(\log \cos \phi _n)}{dx_n}\right| \le C \left| \frac{d(\log \cos \phi _n)}{d\theta _n}\right| \le \frac{C}{\cos \phi _n}. \end{aligned}$$

The fourth term is the logarithm of the quantity

$$\begin{aligned} 2\rho ^{-1} \tau _{n+1}+ \cos \phi _{n+1}(1+\tau _{n+1}\Omega ^-_{n+1}) \end{aligned}$$

which is bounded from below, but not from above. It is thus (more than) enough to show that, when taking the derivative, all contributions to the numerator are uniformly bounded. This holds immediately by the previous discussion for all the terms except $\frac{2}{\rho } \frac{d\tau _{n+1}}{dx_n}$ which requires further investigation. Note that

$$\begin{aligned} \tau _{n+1}=\mathop {dist}(P(x_n), P(x_{n+1})) \end{aligned}$$

where $P(x_n)$ and $P(x_{n+1})$ are points on the billiard table (and thus on ${{\mathbb {R}}}^2$) associated to the points $x_n\in W_n$ and $x_{n+1}\in W_{n+1}$ on the two scatterers, respectively. In an appropriate reference frame $P(x_n)=(\rho \cos \theta _n,\rho \sin \theta _n)$ hence the $\theta _n$-derivatives of both coordinates are $\ll \rho$, and the same holds for the $\theta _{n+1}$-derivatives of the coordinates of $P(x_{n+1})$. Thus

$$\begin{aligned} \left| \frac{d\tau _{n+1}}{dx_n}\right| \le C \rho , \end{aligned}$$

which is sufficient for our purposes. $\square$

## C Decay of correlation for $\kappa _\rho$.

The main result of this section is the justification of (37), that is

### Proposition C.1

There exist ${\hat{C}}_\rho >0$ and ${\hat{\vartheta }}_\rho <1$ such that

- for any $j \ge 1$ we have
  54 $$\begin{aligned}&\Big | \int _{{{\mathcal {M}}}_0} (e^{it\kappa _\rho }-1)\, (e^{it\kappa _\rho }-1)\circ T_{\rho }^{j}\, d\mu - \int _{{{\mathcal {M}}}_0} (e^{it\kappa _\rho }-1) \,d\mu \int _{{{\mathcal {M}}}_0} (e^{it\kappa _\rho }-1)\,d\mu \Big | \nonumber \\&\quad \le {\hat{C}}_\rho |t|^2 {\hat{\vartheta }}_\rho ^j, \quad \end{aligned}$$
- furthermore, there exist $\bar{C}_{\rho }>0$ and $\bar{\vartheta }_{\rho }<1$ such that for any $j, \ell \ge 1$ we have
  55 $$\begin{aligned} \Big | \int _{{{\mathcal {M}}}_0}&(e^{it\kappa _\rho }-1) R_\rho ^{\ell }(e^{it\kappa _\rho }-1)\, (e^{it\kappa _\rho }-1)\circ T_{\rho }^{j}\, d\mu \nonumber \\&-\int _{{{\mathcal {M}}}_0} (e^{it\kappa _\rho }-1) R_\rho ^{\ell }(e^{it\kappa _\rho }-1)\,d\mu \int _{{{\mathcal {M}}}_0} (e^{it\kappa _\rho }-1)\,d\mu \nonumber \\&-\,\Big (\int _{{{\mathcal {M}}}_0} (e^{it\kappa _\rho }-1)\,d\mu \Big ) \int _{{{\mathcal {M}}}_0} (e^{it\kappa _\rho }-1)\, (e^{it\kappa _\rho }-1)\circ T_{\rho }^{j}\, d\mu \nonumber \\&+\,\Big (\int _{{{\mathcal {M}}}_0} (e^{it\kappa _\rho }-1)\,d\mu \Big )^3\Big |\ \le \ \bar{C}_{\rho }|t|^2 \bar{\vartheta }_{\rho }^{\ell +j}. \end{aligned}$$

The $\rho$-dependence of this exponential rate gives the main source of unknown dependence on $\rho$ in the main results of our paper. During the proof we will point out the exact sources of unknown dependence of $\bar{C}_{\rho }>0$ and $\bar{\vartheta }_{\rho }<1$ on $\rho$.

Let us make some comments on the relations of the two estimates of Proposition C.1. We will first prove (54) with some ${\hat{C}}_\rho >0$ and ${\hat{\vartheta }}_\rho <1$ that we can explicitly relate to the correlation decay rates of the map $T_{\rho }$ on Hölder functions, as expressed in (66) below. Then we extend our argument to obtain (55) for some $\bar{C}_{\rho }>{\hat{C}}_\rho$ and $\bar{\vartheta }_{\rho }\in ({\hat{\vartheta }}_\rho ,1)$. Obtaining relations similar to (66) for the constants $\bar{C}_{\rho }$ and $\bar{\vartheta }_{\rho }$ seems quite difficult and we do not push this point.

The proof of Proposition C.1 consists in: a) reconsider [11, Proposition 9.1]; b) only for (55), work with a version of $R_\rho$ with spectral gap in a Banach space embedded in some $L^p$ space with $p>1$. Item a) is needed in order to obtain the bound $|t|^2$ and the decay of correlation in *j*. Item b) is needed to obtain the joint decay in *j* and $\ell$. Item b) is possible because for every $\rho >0$, *there exists* a Young tower $\Delta _\rho$ and a tower map $T_{\Delta _\rho }$ associated with the billiard map $T_\rho$; this is ensured by the construction in [9, 34]. We emphasize that we will not exploit any fine dependence on $\rho$ of $T_{\Delta _\rho }$ (the mere existence is enough), which is why this part of our arguments can be worked on the Young tower $\Delta _\rho$.

### C.1 Standard pair argument

In this section we reconsider [11, Proposition 9.1]. Let us introduce truncation levels $H,{\hat{H}} > 0$ to be fixed later and

$$\begin{aligned} \kappa _\rho '=\kappa _\rho \cdot {\textbf {1}}_{|\kappa _\rho |\le H} \qquad \qquad&\kappa _\rho ''=\kappa _\rho -\kappa _\rho ';\\ \kappa _\rho '''=\kappa _\rho \cdot {\textbf {1}}_{|\kappa _\rho |\le {\hat{H}}} \qquad \qquad&\kappa _\rho ''''=\kappa _\rho -\kappa _\rho '''. \end{aligned}$$

As $|\kappa _\rho | \asymp |\xi | m$ on $D_{\xi ,m}$, the truncation $\kappa _\rho '$ restricts $\kappa _\rho$ to the cells $D_{\xi ,m}$ with $m\le H |\xi |^{-1}$.

The result we will use in the proof of Proposition C.1 below is

#### Lemma C.2

For any $c_0>2$ we have

(i)’ $$\int |\kappa _\rho '| \cdot |\kappa _\rho ''''| \circ T_{\rho }^j \, d\mu \le CH^2 {\hat{H}}^{-1} \rho ^{-3}$$,
(ii)’ $$\int |\kappa _\rho '' | \cdot |\kappa _\rho | \circ T_{\rho }^j \, d\mu \le C |\log \rho |\cdot \left( H^{-\frac{1}{2} +\frac{1}{2r_0}} \, \log H \, \rho ^{-3-\nu }+ H^{2-c_0} \rho ^{-2-c_0}\right) $$.

Furthermore, for any $q\in \left( 1,\frac{8}{7}-\frac{6}{7(7r_0-1)}\right)$ and $c \in (\frac{q+1}{2-q}\,, \, \frac{1-1/r_0}{2q-2}-1)$,

(i) $$\int |\kappa _\rho '|^q \cdot |\kappa _\rho ''''|^q \circ T_{\rho }^j \, d\mu \le CH^{q+1} {\hat{H}}^{q-2} \rho ^{-3}$$,
(ii) $$\int |\kappa _\rho '' |^q \cdot |\kappa _\rho |^q \circ T_{\rho }^j \, d\mu \le C \Big (H^{-\frac{3}{2}+q+c(q-1)+\frac{1}{2r_0}} \rho ^{c(q-1)-q-2-\nu } + H^{c(q-2)+q+1} \rho ^{-1-q-c(2-q)}\Big )$$.

#### Remark C.3

Let $q(r_0)=\frac{8}{7}-\frac{6}{7(7r_0-1)}$, the upper bound on *q* for $r_0$ fixed. Furthermore, let $c_1(q)=\frac{q+1}{2-q}$ and $c_2(q)=\frac{1-1/r_0}{2q-2}-1$, the lower and upper bounds on *c* for *q* fixed. Note that $c_1(q)$ is increasing in *q*, while $c_2(q)$ is decreasing in *q*, and $c_1(q(r_0))=c_2(q(r_0))$. Also $c_1(1)=2$ and $c_2(1)=\infty$, which is in accordance with the conditions on $c_0$. Note also that:

- The condition $c<c_2(q)=\frac{1-\frac{1}{r_0}}{2q-2}-1$ is equivalent to $q+c(q-1)<\frac{3}{2}-\frac{1}{2r_0}$. This ensures that the power of *H* in the first term of (ii) is negative.
- Since $c>c_1(q)=\frac{q+1}{2-q}$, the power of *H* in the second term of (ii) is negative.
- Choosing ${\hat{H}}=H^c$, the power of *H* in (i) is also negative, again for $c>c_1(q)=\frac{q+1}{2-q}$.

*Standard pairs and families* Let us recall some terminology related to standard pairs, see also [11, page 29]. A *standard pair* $\ell =(W,h_W)$ is a regular unstable curve *W* that supports a dynamically log-Hölder continuous probability density $h_W$. As such, it can be regarded as a probability measure on the phase space ${{\mathcal {M}}}$, which will be denoted by $\ell$, too.

A *standard family* is a collection of standard pairs ${{\mathcal {G}}}=\{\ell _a\}$, $a\in {{\mathcal {A}}}$ equipped with a probability factor measure $\lambda _{{{\mathcal {G}}}}$ on ${{\mathcal {A}}}$. This induces a probability measure ${{\mathbb {P}}}_{{{\mathcal {G}}}}$ on ${{\mathcal {M}}}$.

For a standard pair $\ell =(W,h_W)$ any $x\in W$ splits *W* into two subcurves, let $r_W(x)$ denote the length of the shorter, and let ${{\mathcal {Z}}}_{\ell }=\sup _{\varepsilon >0} \varepsilon ^{-1} \ell (r_W\le \varepsilon )$. By Hölder continuity of $\log h_W$, $\ell$ is equivalent to the normalized Lebesgue measure on *W* and thus ${{\mathcal {Z}}}_{\ell } \asymp |W|^{-1}$. This generalizes for the *Z*-function of a standard family ${{\mathcal {Z}}}_{{{\mathcal {G}}}} \asymp \int \frac{\lambda _{{{\mathcal {G}}}}(a)}{|W_a|} dm_W$.

The $T_{\rho }$-image of a standard pair is a countable collection of standard pairs. Hence, the image of a standard family is a standard family. Given a standard family ${{\mathcal {G}}}$, for $n\ge 1$, ${{\mathcal {G}}}_n$ denotes the $T_{\rho }^n$-image of ${{\mathcal {G}}}$. It follows from the growth lemma (Proposition 3.1) that there exists $\vartheta <1$ and $C_1,C_2>0$ such that

$$\begin{aligned} {{\mathcal {Z}}}_{{{\mathcal {G}}}_n}\le C_1\vartheta ^n {{\mathcal {Z}}}_{{{\mathcal {G}}}} + C_2 \delta _0^{-1} \end{aligned}$$

where $\delta _0 \asymp \rho ^{\nu }$ (see (16) and Remark 3.2, part (i)). As consequence, for any standard pair and $n\ge 1$

56 $$\begin{aligned} {{\mathcal {Z}}}_{{{\mathcal {G}}}_n}\le C \max ({{\mathcal {Z}}}_{{{\mathcal {G}}}_1}, \rho ^{-\nu }). \end{aligned}$$

*Cells* For $\xi \in {{\mathbb {Z}}}^2$ such that the corridor is opened up, and for $m\in {{\mathbb {Z}}}$ let $D_{\xi ,m}\subset {{\mathcal {M}}}$ denote the set of points for which $\kappa _\rho =m\xi +\xi '$. The geometric properties of $D_{\xi ,m}$ and its image $T_{\rho }D_{\xi ,m}$ will play an important role in the argument. $T_{\rho }D_{\xi ,m}$ is depicted in Fig. 2. A similar description applies to $D_{\xi ,m}$; it is delimited by a long singularity curve, decreasing in the $(\theta ,\varphi )$ coordinates, which is connected to the boundary of ${{\mathcal {M}}}$ by two shorter decreasing singularity curves, of length $\asymp (|\xi |\rho m)^{-1/2}$, running at a distance $\asymp (|\xi | m)^{-2}$ from each other. Further properties:

- $\mu (D_{\xi ,m})=\mu (T_{\rho }D_{\xi ,m})\asymp \rho ^{-1} |\xi |^{-3}m^{-3}$ (due to the factor $\cos \phi$ in the measure);
- an unstable curve may intersect $D_{\xi ,m}$ in a subcurve of length $\le C (|\xi | m)^{-2}$;
- $T_{\rho }D_{\xi ,m}$ intersects homogeneity strips of index $k\ge C(\rho |\xi | m)^{\frac{1}{2r_0}}$

If $\ell =(W,h_W)$ is a standard pair, then it can intersect $D_{\xi ,m}$ in a subcurve of length $\le C (|\xi | m)^{-2}$, thus the intersection has probability bounded above by $C (|\xi | m)^{-2}|W|^{-1} \asymp {{\mathcal {Z}}}_{\ell } (|\xi | m)^{-2}$. It follows that for a standard family ${{\mathcal {G}}}$ we have

57 $$\begin{aligned} {{\mathbb {P}}}_{{{\mathcal {G}}}} (D_{\xi ,m})\le C (|\xi | m)^{-2} {{\mathcal {Z}}}_{{{\mathcal {G}}}}. \end{aligned}$$

Our argument below follows the proof of [11, Proposition 9.1] taking into account that the corridor structure depends on $\rho$.

#### Proof of Lemma C.2

For item (i), using $\mu (T_{\rho }^{-j}D_{\hat{\xi },{\hat{m}}}) \ll \rho ^{-1} {\hat{m}}^{-3} |\hat{\xi }|^{-3}$ as well as Lemma A.4 several times, we get

$$\begin{aligned} \int |\kappa _\rho '|^q \cdot |\kappa _\rho ''''\circ T_{\rho }^j|^q \, d\mu&\le C \sum _{\xi }\sum _{\hat{\xi }}|\xi |^q |\hat{\xi }|^q \sum _{m=1}^{\frac{H}{|\xi |}} \sum _{\hat{m}=\frac{{\hat{H}}}{|\hat{\xi }|}}^{\infty } m^q \hat{m}^q \mu (D_{\xi ,m}\cap T_{\rho }^{-n}D_{\hat{\xi },\hat{m}}) \\&\le C \rho ^{-1} \sum _{\xi }\sum _{\hat{\xi }}|\xi |^q |\hat{\xi }|^q \sum _{m=1}^{\frac{H}{|\xi |}} m^q \sum _{\hat{m}=\frac{{\hat{H}}}{|\hat{\xi }|}}^{\infty } \hat{m}^{q-3} |\hat{\xi }|^{-3} \\&\le C \rho ^{-1} \sum _{\xi }H^{q+1} |\xi |^{-1} \sum _{\hat{\xi }}{\hat{H}}^{q-2} |\hat{\xi }|^{-1} \le CH^{q+1} {\hat{H}}^{q-2} \rho ^{-3}. \end{aligned}$$

We will take ${\hat{H}}=H^c$ for $c>0$ to be determined. To get a negative power of *H*, we need $q<2$ and $c>\frac{q+1}{2-q}$.

For the proof of (ii), we need to estimate

58 $$\begin{aligned} \int |\kappa _\rho '' |^q\cdot |\kappa _\rho \circ T_{\rho }^j|^q \, d\mu \le C \sum _{\xi }\sum _{\hat{\xi }}|\xi |^q |\hat{\xi }|^q \sum _{m=\frac{H}{|\xi |}}^{\infty } m^q \sum _{\hat{m}=1}^{\infty } \hat{m}^q \mu (D_{\xi ,m}\cap T_{\rho }^{-j}D_{\hat{\xi },\hat{m}}).\nonumber \\ \end{aligned}$$

For different ranges of the indices, we will use two different estimates to bound $\mu (D_{\xi ,m}\cap T_{\rho }^{-j}D_{\hat{\xi },\hat{m}})$. On the one hand, as before, we have

59 $$\begin{aligned} \mu (D_{\xi ,m}\cap T_{\rho }^{-n}D_{\hat{\xi },\hat{m}})\le \mu (D_{\hat{\xi },\hat{m}})\le C \rho ^{-1} |\hat{\xi }|^{-3} \hat{m}^{-3}. \end{aligned}$$

For the other estimate, foliate $D_{\xi ,m}$ with unstable curves |*W*| of length $\asymp (|\xi |m)^{-2}$. The image of any such curve stretches along $T_{\rho }D_{\xi ,m}$, crossing homogeneity strips with indices $k\ge C(\rho |\xi | m)^{\frac{1}{2r_0}}$. The piece of $T_{\rho }W$ in the *k*-th homogeneity strip will be denoted by $T_{\rho }W_k$, it has length $\asymp k^{-r_0-1}$, and its preimage has length

$$\begin{aligned} |W_k|\asymp k^{-r_0-1} \frac{\rho }{|\xi | m k^{r_0}}=\frac{\rho }{|\xi | m k^{2r_0+1}} \end{aligned}$$

as the expansion factor of $T_{\rho }$ on $W_k$ is $\asymp \rho ^{-1} |\xi | m k^{r_0}$. Equipped with the conditional measure induced by $\mu$, *W* is a standard pair $\ell =(W,h_W)$, and its image is a standard family $T_{\rho }\ell$ associated to the curves $T_{\rho }W_k$. To obtain the Z function, we use that the weight of $|T_{\rho }W_k|$ within this family is $\frac{|W_k|}{|W|}$, thus

$$\begin{aligned} {{\mathcal {Z}}}_{T_{\rho }\ell }&\asymp \sum _{k\ge C(\rho |\xi | m)^{\frac{1}{2r_0}}} \frac{|W_k|}{|W|} |T_{\rho }W_k|^{-1} \asymp \sum _{k\ge C(\rho |\xi | m)^{\frac{1}{2r_0}}} \frac{\rho |\xi |^2 m^2}{|\xi | m k^{2r_0+1}} k^{r_0+1} \\&\asymp \rho m |\xi | \sum _{k\ge C(\rho |\xi | m)^{\frac{1}{2r_0}}} k^{-r_0} \asymp (\rho m |\xi |)^{\frac{1}{2}+\frac{1}{2r_0}}. \end{aligned}$$

This analysis applies to all the curves in the foliation. Accordingly, $\mu$ conditioned on $D_{\xi ,m}$ can be regarded as a standard family ${{\mathcal {G}}}$, and the ${{\mathcal {Z}}}$-function of its $T_{\rho }$-image satisfies

$$\begin{aligned} {{\mathcal {Z}}}_{{{\mathcal {G}}}_1} \asymp C (\rho m |\xi |)^{\frac{1}{2}+\frac{1}{2r_0}}. \end{aligned}$$

For further iterates, it follows form (56) that

$$\begin{aligned} {{\mathcal {Z}}}_{{{\mathcal {G}}}_n} \le C \rho ^{-\nu } (m |\xi |)^{\frac{1}{2}+\frac{1}{2r_0}}. \end{aligned}$$

Now we apply (57) to get

60 $$\begin{aligned} \mu (D_{\xi ,m}\cap T_{\rho }^{-n}D_{\hat{\xi },\hat{m}})&= \mu (D_{\xi ,m}) {{\mathbb {P}}}_{{{\mathcal {G}}}_n} (D_{\hat{\xi },\hat{m}}) \le C \mu (D_{\xi ,m}) {{\mathcal {Z}}}_{{{\mathcal {G}}}_n} |\hat{\xi }|^{-2} \hat{m}^{-2} \nonumber \\&\le C |\hat{\xi }|^{-2}\hat{m}^{-2} |\xi |^{-\frac{5}{2}+\frac{1}{2r_0}} m^{-\frac{5}{2}+\frac{1}{2r_0}} \rho ^{-1-\nu }. \end{aligned}$$

We split (58) into two parts. If $\hat{m}\le m^c$ (for some $c > 0$ to be determined), we use (60) and get

$$\begin{aligned} \sum _{\xi }\sum _{\hat{\xi }}&|\xi |^q |\hat{\xi }|^q \sum _{m=\frac{H}{|\xi |}}^{\infty } m^q \sum _{\hat{m}=1}^{m^c} \hat{m}^q \mu (D_{\xi ,m}\cap T_{\rho }^{-n}D_{\hat{\xi },\hat{m}}) \\&\le C \rho ^{-1-\nu } \sum _{\xi }\sum _{\hat{\xi }}|\xi |^{-\frac{5}{2}+q+\frac{1}{2r_0}} |\hat{\xi }|^{q-2} \sum _{m=\frac{H}{|\xi |}}^{\infty } m^{-\frac{5}{2}+q+\frac{1}{2r_0}} m^{c(q-1)} \\&\le C \rho ^{-1-\nu } H^{-\frac{3}{2}+q+c(q-1)+\frac{1}{2r_0}} \left( \sum _{\xi }|\xi |^{-1-c(q-1)} \right) \left( \sum _{\hat{\xi }}|\hat{\xi }|^{q-2}\right) \\&\le C H^{-\frac{3}{2}+q+c(q-1)+\frac{1}{2r_0}} \rho ^{c(q-1)-q-2-\nu }, \end{aligned}$$

where we have used that because $q+c(q-1)<\frac{3}{2}-\frac{1}{2r_0}$, the contribution of *m* is summable (this condition is equivalent to $c<c_2(q)=\frac{1-\frac{1}{r_0}}{2q-2}$, cf. Remark C.3). Note that if $q=1$ then this contribution is independent of *c*; however, there is an additional factor of $|\log \rho |\cdot \log H$.

For $m > m^c$ we use (59) and get

$$\begin{aligned} \sum _{\xi }\sum _{\hat{\xi }}&|\xi |^q |\hat{\xi }|^q \sum _{m=\frac{H}{|\xi |}}^{\infty } m^q \sum _{\hat{m}=m^c}^{\infty } \hat{m}^q \mu (D_{\xi ,m}\cap T_{\rho }^{-n}D_{\hat{\xi },\hat{m}}) \\&\le C \rho ^{-1} \sum _{\xi }\sum _{\hat{\xi }}|\xi |^q |\hat{\xi }|^{q-3} \sum _{m=\frac{H}{|\xi |}}^{\infty } m^q \sum _{\hat{m}=m^c}^{\infty } \hat{m}^{q-3}\\ {}&\le C \rho ^{-1} \sum _{\xi }\sum _{\hat{\xi }}|\xi |^q |\hat{\xi }|^{q-3} \sum _{m=\frac{H}{|\xi |}}^{\infty } m^{c(q-2)+q} \\&\le C H^{c(q-2)+q+1} \rho ^{-1} \left( \sum _{\xi }|\xi |^{c(2-q)-1} \right) \left( \sum _{\hat{\xi }}|\hat{\xi }|^{q-3}\right) \\ {}&\le C H^{c(q-2)+q+1} \rho ^{-1-q-c(2-q)}, \end{aligned}$$

and in case $q=1$ we still have an additional $|\log \rho |$ factor. The condition of summability $c(q-2)+q<-1$ is satisfied because $c>\frac{q+1}{2-q}$. Summarizing, we need

$$\begin{aligned} 1\le q<2, \qquad q+c(q-1)<\frac{3}{2}-\frac{1}{2r_0}, \qquad \frac{q+1}{2-q}<c. \end{aligned}$$

First we may fix *q* such that

$$\begin{aligned} \frac{3}{2}-\frac{1}{2r_0}>q+\frac{q+1}{2-q}(q-1)=\frac{2q-1}{2-q} \quad \Leftrightarrow \quad q<2-\frac{6}{7-1/r_0} \end{aligned}$$

and then we can fix *c* slightly larger than $\frac{q+1}{2-q}$, such that the conditions are still met. The range of allowed *q* depends on $r_0$, it can never exceed $\frac{8}{7}$; for the traditional $r_0=2$ the upper bound is $\frac{14}{13}$, while for $r_0=5$ the upper bound is $\frac{19}{17}$. $\square$

### C.2 Exploiting the existence of a Young tower for $T_\rho$

Let $({\bar{\Delta }}_\rho , T_{{\bar{\Delta }}_\rho }, \mu _{{\bar{\Delta }}_\rho })$ be the corresponding one-sided Young tower (i.e., with stable leaves quotiented out) and let $R_{{\bar{\Delta }}_\rho }$ be the transfer operator of $T_{{\bar{\Delta }}_\rho }$. Let ${\hat{\kappa }}_\rho$ be the version of $\kappa _\rho$ on ${\bar{\Delta }}_\rho$. We will also use the notations ${\hat{\kappa }}_\rho ', {\hat{\kappa }}_\rho '', {\hat{\kappa }}_\rho ''', {\hat{\kappa }}_\rho ''''$ for the Young tower versions of the truncations $\kappa _\rho ', \kappa _\rho '', \kappa _\rho ''', \kappa _\rho ''''$, respectively, hence for example ${\hat{\kappa }}_\rho '={\hat{\kappa }}_\rho \cdot {\textbf {1}}_{|{\hat{\kappa }}_\rho |\le H}$. Since $\kappa _\rho$ is constant on stable leaves, we have for any $j, \ell \ge 0$,

61 $$\begin{aligned}&\int _{{{\mathcal {M}}}_0} (e^{it\kappa _\rho }-1)\, R_\rho ^{\ell }(e^{it\kappa _\rho }-1)\, (e^{it\kappa _\rho }-1)\circ T_{\rho }^{j}\, d\mu \nonumber \\&\quad =\int _{{\bar{\Delta }}_\rho } (e^{it{\hat{\kappa }}_\rho }-1)\, R_{{\bar{\Delta }}_\rho }^{\ell }(e^{it{\hat{\kappa }}_\rho }-1)\, (e^{it{\hat{\kappa }}_\rho }-1)\circ T_{{\bar{\Delta }}_\rho } ^{j}\, d\mu _{{\bar{\Delta }}_\rho }. \end{aligned}$$

Let *r* be the roof function of the tower $({\bar{\Delta }}_{\rho },\mu _{{\bar{\Delta }}_{\rho }})$. We recall that if $d:= \gcd (r) > 1$, then for every $\rho >0$, $R_{{\bar{\Delta }}_\rho }$, when viewed as an operator acting on the Young Banach space ${{\mathcal {B}}}_{{\bar{\Delta }}_\rho }\subset L^p(\mu _{{\bar{\Delta }}_\rho })$, $p>1$, has a spectral gap (see [9, 34]). As clarified in Remarks C.4 and C.5, the decomposition of $R_{{\bar{\Delta }}_\rho }$ we shall need in the proof below holds when $d>1$.

Before proceeding to the proof of Proposition C.1, we recall one property of the norm the Young space ${{\mathcal {B}}}_{{\bar{\Delta }}_\rho }\subset L^p(\mu _{{\bar{\Delta }}_\rho })$, $p>1$ that we shall need in the proof below. (The precise definition of ${{\mathcal {B}}}_{{\bar{\Delta }}_\rho }$ is not important in the proof below, and we omit it.) For any function that is constant on the partition elements of the Young tower, the involved seminorm is zero. This is the case for ${\hat{\kappa }}_\rho '$ and thus, $\Vert {\hat{\kappa }}_\rho '\Vert _{{{\mathcal {B}}}_{{\bar{\Delta }}_\rho }}\le H$ (and a similar version holds for ${\hat{\kappa }}_\rho '''$).

#### Proof of Proposition C.1

We first prove the statement for the case when $\ell =0$ and point out the required modifications when $\ell \ge 1$.

**Case** ${\varvec{\ell }}=\textbf{0}$. Given (61), in this case we need to show that

62 $$\begin{aligned} \left| \int _{{\bar{\Delta }}_\rho } (e^{it{\hat{\kappa }}_\rho }-1)\, (e^{it{\hat{\kappa }}_\rho }-1)\circ T_{{\bar{\Delta }}_\rho } ^{j}\, \mu _{{\bar{\Delta }}_\rho }-\Big (\int _{{\bar{\Delta }}_\rho } (e^{it{\hat{\kappa }}_\rho }-1)\, \mu _{{\bar{\Delta }}_\rho }\Big )^2\right| \le {\hat{C}}_\rho |t|^2 {\hat{\vartheta }}_\rho ^{j}, \end{aligned}$$

for some $\rho$-dependent constants ${\hat{\vartheta }}_\rho <1$ and ${\hat{C}}_\rho > 0$.

Throughout this proof, we let $\kappa _\rho ', \kappa _\rho '', \kappa _\rho ''', \kappa _\rho ''''$ also denote their corresponding versions on the tower $\Delta _\rho$ and the context in which they appear will make it clear which version we are referring to.

Write

$$\begin{aligned}&\int _{{\bar{\Delta }}_\rho } (e^{it{\hat{\kappa }}_\rho }-1)\, (e^{it{\hat{\kappa }}_\rho }-1)\circ T_{{\bar{\Delta }}_\rho } ^{j}\, d \mu _{{\bar{\Delta }}_\rho }= \int _{{\bar{\Delta }}_\rho }(e^{i{\hat{\kappa }}_\rho t}-e^{i\kappa _\rho 't}) \cdot (e^{i{\hat{\kappa }}_\rho t}-1) \circ T_{{\bar{\Delta }}_\rho }^j \, d \mu _{{\bar{\Delta }}_\rho }\\&\qquad + \int _{{\bar{\Delta }}_\rho } (e^{i\kappa _\rho ' t}-1) \cdot (e^{i{\hat{\kappa }}_\rho t}-e^{i\kappa _\rho '''t}) \circ T_{{\bar{\Delta }}_\rho }^j \, d \mu _{{\bar{\Delta }}_\rho }\\ {}&\qquad + \int _{{\bar{\Delta }}_\rho } (e^{i\kappa _\rho ' t}-1) \cdot (e^{i\kappa _\rho ''' t}-1) \circ T_{{\bar{\Delta }}_\rho }^j \, d \mu _{{\bar{\Delta }}_\rho }\\&\quad = \int _{{\bar{\Delta }}_\rho } e^{i\kappa _\rho 't} \cdot (e^{i\kappa _\rho '' t}-1) \cdot (e^{i{\hat{\kappa }}_\rho t}-1) \circ T_{{\bar{\Delta }}_\rho }^j \, d \mu _{{\bar{\Delta }}_\rho }\\ {}&\qquad + \int _{{\bar{\Delta }}_\rho } (e^{i\kappa _\rho ' t}-1) \cdot e^{i\kappa _\rho '''t} \circ T_{{\bar{\Delta }}_\rho }^j \cdot (e^{i\kappa _\rho '''' t}-1) \circ T_{{\bar{\Delta }}_\rho }^j \, d \mu _{{\bar{\Delta }}_\rho }\\&\qquad + \int _{{\bar{\Delta }}_\rho } (e^{i\kappa _\rho ' t}-1) \cdot (e^{i\kappa _\rho ''' t}-1) \circ T_{{\bar{\Delta }}_\rho }^j\, d \mu _{{\bar{\Delta }}_\rho } =I_1(t,\rho )+I_2(t,\rho )+I_3(t,\rho ). \end{aligned}$$

For $I_3(t,\rho )$ we use the exponential decay of correlation (see Remark C.4 below for the case that the roof function *r* of the tower has $\gcd (r) > 1$). This gives the only source of unknown dependence on $\rho$ in the case $m=0$. More precisely, for every $\rho >0$, there exists ${\hat{\theta }}_\rho <1$ and $C_\rho >0$ so that

63 $$\begin{aligned}{} & {} \left| I_3(t,\rho ) - \int _{{\bar{\Delta }}_\rho } (e^{i\kappa _\rho 't}-1) \, d \mu _{{\bar{\Delta }}_\rho } \int _{{\bar{\Delta }}_\rho } (e^{i\kappa _\rho '''t}-1) \, d \mu _{{\bar{\Delta }}_\rho } \right| \nonumber \\{} & {} \quad \le C_{\rho } \, {\hat{\theta }}^j_{\rho }\, \Vert e^{it\kappa _\rho ' t}-1 \Vert _{{{\mathcal {B}}}_{\Delta _\rho }} \, \Vert e^{it\kappa _\rho ''' t}-1 \Vert _{{{\mathcal {B}}}_{\Delta _\rho }} \nonumber \\{} & {} \quad \le C_{\rho }\, {\hat{\theta }}^j_{\rho } H \, {\hat{H}} \, |t|^2. \end{aligned}$$

Thus,

$$\begin{aligned}&\left| I_3(t,\rho )-\Big (\int _{{\bar{\Delta }}_\rho } (e^{it{\hat{\kappa }}_\rho }-1)\, \mu _{{\bar{\Delta }}_\rho }\Big )^2\right| \le C_{\rho }\, {\hat{\theta }}^j_{\rho } H \, {\hat{H}} \, |t|^2\\&\qquad + \Big | \int _{{\bar{\Delta }}_\rho } (e^{i\kappa _\rho 't}-1) \, d \mu _{{\bar{\Delta }}_\rho } \int _{{\bar{\Delta }}_\rho } (e^{i\kappa _\rho '''t}-1) \, d \mu _{{\bar{\Delta }}_\rho }\\ {}&\qquad - \int _{{\bar{\Delta }}_\rho } (e^{i{\hat{\kappa }}_\rho t}-1) \, d \mu _{{\bar{\Delta }}_\rho } \int _{{\bar{\Delta }}_\rho } (e^{i{\hat{\kappa }}_\rho t}-1) \, d \mu _{{\bar{\Delta }}_\rho }\Big |\\&\quad =C_{\rho }\, {\hat{\theta }}^j_{\rho } H \, {\hat{H}} \, |t|^2+ |J(t,\rho )|. \end{aligned}$$

By definition,

$$\begin{aligned} |J(t,\rho )|= & {} \Big | \int _{{{\mathcal {M}}}_0}(e^{i\kappa _\rho 't}-1) \, d \mu \int _{{{\mathcal {M}}}_0} (e^{i\kappa _\rho '''t}-1) \, d \mu \\ {}{} & {} - \int _{{{\mathcal {M}}}_0} (e^{i{\hat{\kappa }}_\rho t}-1) \, d \mu \int _{{{\mathcal {M}}}_0} (e^{i{\hat{\kappa }}_\rho t}-1) \,d \mu \Big | \end{aligned}$$

and we note that $J(t,\rho )$ is bounded by the sum of

$$\begin{aligned} \int _{{{\mathcal {M}}}_0} |e^{i\kappa _\rho ' t} \cdot (e^{i t\kappa _\rho ''}-1)| \, d \mu \,\int _{{{\mathcal {M}}}_0} |e^{i t\kappa _\rho ''' t}-1| \, d \mu \le |t|^2 \int |\kappa _\rho | 1_{\{ \kappa _\rho > H\}} \, d \mu \int _{{{\mathcal {M}}}_0}|\kappa _\rho '''| \, d \mu \end{aligned}$$

and a similar term with ${\hat{H}}$ instead of *H*. Using the Hölder inequality (with exponents $\frac{2}{1+\delta }$ and $\frac{2}{1-\delta }$), the tail behaviour of $\kappa _\rho$ and Lemma A.5, we obtain that

$$\begin{aligned} \int _{{{\mathcal {M}}}_0} |\kappa _\rho | 1_{\{|\kappa _\rho |> H\}} \, d\mu \le \Vert \kappa _\rho \Vert _{L^{2/(1+\delta )}} \, \mu (|\kappa _\rho |>H)^{(1-\delta )/2} \ll \rho ^{-1} H^{-(1-\delta )}. \end{aligned}$$

Also $$\int _{{{\mathcal {M}}}_0} |\kappa _\rho '''| d\mu \le \Vert \kappa _\rho \Vert _{L^1(\mu )} \ll \rho ^{-1}$$. Hence,

64 $$\begin{aligned} |J(t,\rho )| \ll |t|^2 \rho ^{-2} \left( H^{-(1-\delta )} + {\hat{H}}^{-(1-\delta )} \right) . \end{aligned}$$

Finally, note that

65 $$\begin{aligned} |I_1(t,\rho )+I_2(t,\rho )|&\le |t|^2 \int _{{\bar{\Delta }}_\rho } |\kappa _\rho ''| \cdot |\kappa _\rho |\circ T_{{\bar{\Delta }}_\rho }^j \, d \mu _{{\bar{\Delta }}_\rho } + |t|^2 \int _{{\bar{\Delta }}_\rho } |\kappa _\rho '| \cdot |\kappa _\rho ''''|\circ T_{{\bar{\Delta }}_\rho }^j \, d \mu _{{\bar{\Delta }}_\rho }\nonumber \\&=|t|^2\left( \int _{{{\mathcal {M}}}_0} |\kappa _\rho ''| \cdot |\kappa _\rho |\circ T_{{\bar{\Delta }}_\rho }^j \, d \mu +\int _{{{\mathcal {M}}}_0} |\kappa _\rho '| \cdot |\kappa _\rho ''''|\circ T_{{\bar{\Delta }}_\rho }^j \, d \mu \right) . \end{aligned}$$

For this $\ell =0$ case, if we fix any $r_0\ge 2$ (taking into account that $\hat{H}=H^{c_0}$), then we may bound the coefficients of $|t|^2$ in $|J(t,\rho )|$ from (64), $|I_1(t,\rho )|$ and $|I_2(t,\rho )|$ from (65), respectively by

$$\begin{aligned} \rho ^{-2} H^{-(1-\delta )}; \qquad H^{-\frac{1}{5}} \rho ^{-4} + H^{2-c_0}\rho ^{-\frac{11}{5}-c_0}, \qquad H^{2-c_0}\rho ^{-3}, \end{aligned}$$

where in the bound for $|I_1(t,\rho )|$ the exponents of *H* and $\rho$ have been slightly decreased to bound the logarithmic factors. Fixing $c_0=\frac{11}{5}$ and $\delta =\frac{4}{5}$, all these are dominated by $H^{-\frac{1}{5}}\rho ^{-\frac{22}{5}}$. On the other hand the coefficient of $|t|^2$ in $|I_3(t,\rho )|$ is $C_{\rho }\, {\hat{\theta }}^j_{\rho } H^{c_0+1}=C_{\rho }\, {\hat{\theta }}^j_{\rho } H^{\frac{16}{5}}$. Thus letting $H=\left( C_{\rho }^{-1}\, {\hat{\theta }}^{-j}_{\rho } \rho ^{-\frac{22}{5}}\right) ^{\frac{5}{17}}$ we conclude that all terms are dominated by

66 $$\begin{aligned} \rho ^{-\frac{352}{85}} C_{\rho }^{\frac{1}{17}}\, ({\hat{\theta }}_{\rho }^{\frac{1}{17}})^j;\qquad \text {thus we let}\qquad \hat{C}_{\rho }=\rho ^{-\frac{352}{85}} C_{\rho }^{\frac{1}{17}}, \ \hat{\vartheta }_{\rho }={\hat{\theta }}_{\rho }^{\frac{1}{17}}. \end{aligned}$$

**Case** $\ell \ge 1$. The main differences in this case come down to dealing with integrals containing unbounded terms $\kappa _\rho ''$ and $\kappa _\rho ''''$ in such a way that can gain exponential decay in $\ell$ and then proceed as in the case $\ell =0$ treated above. To do this, we exploit that ${{\mathcal {B}}}_{{\bar{\Delta }}_\rho }\subset L^p(\mu _{{\bar{\Delta }}_\rho })$.

Using (61), we need to estimate

$$\begin{aligned} J(t,\rho )&:= \int _{{\bar{\Delta }}_\rho } (e^{it{\hat{\kappa }}_\rho }-1)\, R_{{\bar{\Delta }}_\rho }^{\ell }(e^{it\kappa _\rho }-1)\, (e^{it{\hat{\kappa }}_\rho }-1)\circ T_{{\bar{\Delta }}_\rho } ^{j}\, d\mu _{{\bar{\Delta }}_\rho }\\&\quad -\int _{{\bar{\Delta }}_\rho } (e^{it{\hat{\kappa }}_\rho }-1) R_{{\bar{\Delta }}_\rho }^{\ell }(e^{it{\hat{\kappa }}_\rho }-1)\, d\mu _{{\bar{\Delta }}_\rho }\, \int _{{\bar{\Delta }}_\rho } (e^{it{\hat{\kappa }}_\rho }-1)\,d\mu _{{\bar{\Delta }}_\rho }\\&\quad -\int _{{\bar{\Delta }}_\rho } (e^{it{\hat{\kappa }}_\rho }-1)\,d\mu _{{\bar{\Delta }}_\rho }\, \int _{{\bar{\Delta }}_\rho } (e^{it{\hat{\kappa }}_\rho }-1)\, (e^{it{\hat{\kappa }}_\rho }-1)\circ T_{\rho }^{j}\, d\mu _{{\bar{\Delta }}_\rho }\\&\quad +\Big (\int _{{\bar{\Delta }}_\rho } (e^{it{\hat{\kappa }}_\rho }-1)\,d\mu _{{\bar{\Delta }}_\rho }\Big )^3. \end{aligned}$$

By Remark C.4, for every $\rho >0$ and for every $\ell \ge 1$,

67 $$\begin{aligned} R_{{\bar{\Delta }}_\rho }^{\ell }(e^{it{\hat{\kappa }}_\rho }-1)-\int _{{\bar{\Delta }}_\rho } (e^{it\kappa _\rho }-1)\,d\mu _{{\bar{\Delta }}_\rho }&=Q_{{\bar{\Delta }}_\rho }^{\ell }(e^{it\kappa _\rho }-1),\quad \quad \Vert Q_{{\bar{\Delta }}_\rho }^{\ell }(e^{it{\hat{\kappa }}_\rho }-1)\Vert _{{{\mathcal {B}}}_{{\bar{\Delta }}_\rho }}\nonumber \\ {}&\le C_{\rho }\, {\hat{\theta }}^\ell _{\rho }, \end{aligned}$$

for some $\rho$-dependent $C_\rho$ and ${\hat{\theta }}_{\rho }<1$. This is the first source of unknown dependence on $\rho$. Since ${{\mathcal {B}}}_{{\bar{\Delta }}_\rho }\subset L^p(\mu _{{\bar{\Delta }}_\rho })$,

68 $$\begin{aligned} \Vert Q_{{\bar{\Delta }}_\rho }^{\ell }(e^{it{\hat{\kappa }}_\rho }-1)\Vert _{L^p(\mu _{{\bar{\Delta }}_\rho })}\le C_{\rho }^0\, {\hat{\theta }}^\ell _{\rho }, \end{aligned}$$

for some $\rho$-dependent $C_\rho ^0$. This is the second source of unknown dependence on $\rho$.

With these specified, we can write

$$\begin{aligned} J(t,\rho )&=\int _{{\bar{\Delta }}_\rho } (e^{it{\hat{\kappa }}_\rho }-1)\, Q_{{\bar{\Delta }}_\rho }^{\ell }(e^{it{\hat{\kappa }}_\rho }-1)\, (e^{it{\hat{\kappa }}_\rho }-1)\circ T_{{\bar{\Delta }}_\rho } ^{j}\, d\mu _{{\bar{\Delta }}_\rho }\\&\quad -\int _{{\bar{\Delta }}_\rho } (e^{it{\hat{\kappa }}_\rho }-1) Q_{{\bar{\Delta }}_\rho }^{\ell }(e^{it{\hat{\kappa }}_\rho }-1)\, d\mu _{{\bar{\Delta }}_\rho }\, \int _{{\bar{\Delta }}_\rho } (e^{it{\hat{\kappa }}_\rho }-1)\,d\mu _{{\bar{\Delta }}_\rho }\\ {}&=E(t,\rho ) -G(t,\rho ). \end{aligned}$$

Rearranging as in the case $\ell =0$,

$$\begin{aligned} E(t,\rho )&= \int _{{\bar{\Delta }}_\rho }(e^{i{\hat{\kappa }}_\rho t}-e^{i{\hat{\kappa }}_\rho 't}) \,Q_{{\bar{\Delta }}_\rho }^{\ell }(e^{it\kappa _\rho }-1)\, \, (e^{i{\hat{\kappa }}_\rho t}-1) \circ T_{{\bar{\Delta }}_\rho }^j \, d \mu _{{\bar{\Delta }}_\rho }\\&\qquad + \int _{{\bar{\Delta }}_\rho } (e^{i\kappa _\rho ' t}-1) \,Q_{{\bar{\Delta }}_\rho }^{\ell }(e^{it{\hat{\kappa }}_\rho }-1)\, (e^{i{\hat{\kappa }}_\rho t}-e^{i\kappa _\rho '''t}) \circ T_{{\bar{\Delta }}_\rho }^j \, d \mu _{{\bar{\Delta }}_\rho }\\&\qquad + \int _{{\bar{\Delta }}_\rho } (e^{i\kappa _\rho ' t}-1)\, Q_{{\bar{\Delta }}_\rho }^{\ell }\,(e^{it{\hat{\kappa }}_\rho }-1)\, (e^{i\kappa _\rho ''' t}-1) \circ T_{{\bar{\Delta }}_\rho }^j \, d \mu _{{\bar{\Delta }}_\rho }\\&\quad = \int _{{\bar{\Delta }}_\rho } e^{i\kappa _\rho 't} \,Q_{{\bar{\Delta }}_\rho }^{\ell }(e^{it{\hat{\kappa }}_\rho }-1)\, (e^{i\kappa _\rho '' t}-1) \cdot (e^{i{\hat{\kappa }}_\rho t}-1) \circ T_{{\bar{\Delta }}_\rho }^j \, d \mu _{{\bar{\Delta }}_\rho }\\&\qquad + \int _{{\bar{\Delta }}_\rho } (e^{i\kappa _\rho ' t}-1) \,Q_{{\bar{\Delta }}_\rho }^{\ell }(e^{it{\hat{\kappa }}_\rho }-1)\, e^{i\kappa _\rho '''t} \circ T_{{\bar{\Delta }}_\rho }^j \cdot (e^{i\kappa _\rho '''' t}-1) \circ T_{{\bar{\Delta }}_\rho }^j \, d \mu _{{\bar{\Delta }}_\rho }\\&\qquad +\int _{{\bar{\Delta }}_\rho } (e^{i\kappa _\rho ' t}-1) \,Q_{{\bar{\Delta }}_\rho }^{\ell }(e^{it{\hat{\kappa }}_\rho }-1)\, (e^{i\kappa _\rho ''' t}-1) \circ T_{{\bar{\Delta }}_\rho }^j\, d \mu _{{\bar{\Delta }}_\rho }\\ {}&=E_1(t,\rho )+E_2(t,\rho )+E_3(t,\rho ). \end{aligned}$$

Let $q\in (1,\frac{8}{7}-\frac{6}{7r_0-1})$ so that Lemma C.2 holds. By the Hölder inequality with $\frac{1}{p}+\frac{1}{q}=1$ and (68),

$$\begin{aligned} |E_1(t,\rho )+E_2(t,\rho )|&\le \Vert Q_{{\bar{\Delta }}_\rho }^{\ell }(e^{it{\hat{\kappa }}_\rho }-1)\Vert _{L^p(\mu _{{\bar{\Delta }}_\rho })}\, |t|^2\Vert \, |\kappa _\rho ''| \cdot |{\hat{\kappa }}_\rho |\circ T_{{\bar{\Delta }}_\rho }^j \Vert _{L^q(\mu _{{\bar{\Delta }}_\rho })}\\&\quad +\Vert Q_{{\bar{\Delta }}_\rho }^{\ell }(e^{it{\hat{\kappa }}_\rho }-1)\Vert _{L^p(\mu _{{\bar{\Delta }}_\rho })}\, |t|^2\Vert \, |\kappa _\rho '| \cdot |\kappa _\rho ''''|\circ T_{{\bar{\Delta }}_\rho }^j \Vert _{L^q(\mu _{{\bar{\Delta }}_\rho })}\\&\le C_{\rho }^0\, {\hat{\theta }}^{\ell }_{\rho }\,|t|^2\, \left( \Vert |\kappa _\rho ''| \cdot |{\hat{\kappa }}_\rho |\circ T_{{\bar{\Delta }}_\rho }^j \Vert _{L^q(\mu _{{\bar{\Delta }}_\rho })} +\Vert |\kappa _\rho '| \cdot |\kappa _\rho ''''|\circ T_{{\bar{\Delta }}_\rho }^j \Vert _{L^q(\mu _{{\bar{\Delta }}_\rho })}\right) . \end{aligned}$$

Similar to estimating (65), using Lemma C.2 and Remark C.3 and without trying for optimal bounds, we can pick *q* close to 1 and $c_0 < \frac{5}{2}$ such that $c_0(q-2)+q+1 = -\frac{1}{5}$. For these values,

69 $$\begin{aligned} |E_1(t,\rho )+E_2(t,\rho )|\le C\,C_{\rho }^0\, {\hat{\theta }}^{\ell }_{\rho }\,|t|^2\, H^{-\frac{1}{5q}} \rho ^{\frac{-5}{q}}. \end{aligned}$$

Next, let

$$\begin{aligned} L_1(t,\rho )&=\int _{{\bar{\Delta }}_\rho } (e^{i\kappa _\rho ' t}-1) \,Q_{{\bar{\Delta }}_\rho }^{\ell }(e^{it{\hat{\kappa }}_\rho }-1)\, (e^{i\kappa _\rho ''' t}-1) \circ T_{{\bar{\Delta }}_\rho }^j\, d \mu _{{\bar{\Delta }}_\rho }\\&\quad -\int _{{\bar{\Delta }}_\rho } (e^{it\kappa _\rho '}-1) Q_{{\bar{\Delta }}_\rho }^{\ell }(e^{it{\hat{\kappa }}_\rho }-1)\, d\mu _{{\bar{\Delta }}_\rho }\, \int _{{\bar{\Delta }}_\rho } (e^{it\kappa _\rho '''}-1)\,d\mu _{{\bar{\Delta }}_\rho } \end{aligned}$$

and note that

$$\begin{aligned} E_3(t,\rho )-G(t,\rho )&=L_1(t,\rho )-\int _{{\bar{\Delta }}_\rho } (e^{it{\hat{\kappa }}_\rho }-e^{it\kappa _\rho '}) Q_{{\bar{\Delta }}_\rho }^{\ell }(e^{it{\hat{\kappa }}_\rho }-1)\, d\mu _{{\bar{\Delta }}_\rho }\, \int _{{\bar{\Delta }}_\rho } (e^{it{\hat{\kappa }}_\rho }-1)\,d\mu _{{\bar{\Delta }}_\rho }\\&\quad -\int _{{\bar{\Delta }}_\rho } (e^{it{\hat{\kappa }}_\rho }-1) Q_{{\bar{\Delta }}_\rho }^{\ell }(e^{it{\hat{\kappa }}_\rho }-1)\, d\mu _{{\bar{\Delta }}_\rho }\, \int _{{\bar{\Delta }}_\rho } (e^{it{\hat{\kappa }}_\rho }-e^{it\kappa _\rho '''})\,d\mu _{{\bar{\Delta }}_\rho }\\&=L_1(t,\rho )-L_2(t,\rho )-L_3(t,\rho ). \end{aligned}$$

By the exponential decay of correlations as in (63) as well as (68):

$$\begin{aligned} |L_1(t,\rho )|&\le C_{\rho }\, {\hat{\theta }}^j_{\rho } H \, {\hat{H}} \, |t|^2\Vert Q_{{\bar{\Delta }}_\rho }^{\ell }(e^{it{\hat{\kappa }}_\rho }-1)\Vert _{L^p(\mu _{{\bar{\Delta }}_\rho })} \le C_{\rho }\,C_{\rho }^0\, {\hat{\theta }}^{\ell }_{\rho } \, |t|^2 \, H^{1+c_0}, \end{aligned}$$

where as before $c_0 < \frac{5}{2}$. Finally, by the equation before (64), we have

$$\begin{aligned} |L_2(t,\rho )|\le |t^2|\,\rho ^{-1} H^{-(1-\delta )}\Vert Q_{{\bar{\Delta }}_\rho }^{\ell }(e^{it{\hat{\kappa }}_\rho }-1)\Vert _{L^p(\mu _{{\bar{\Delta }}_\rho })} \le C_{\rho }\, \,C_{\rho }^0\,{\hat{\theta }}^{\ell }_{\rho }|t^2|\,\rho ^{-1} H^{-(1-\delta )}. \end{aligned}$$

A similar argument applies to $L_3(t,\rho )$.

The conclusion follows with a similar choice of *H* as in the case $\ell =0$ treated above. $\square$

#### Remark C.4

Let *r* be the roof function of the one-sided tower map $({\bar{\Delta }}_{\rho },\mu _{{\bar{\Delta }}_{\rho }})$. If $d:= \gcd (r) > 1$, then $T_{{\bar{\Delta }}}$ is not mixing on the Banach space ${{\mathcal {B}}}_{{\bar{\Delta }}_\rho }$. However, the underlying billiard map $T_{\rho }$ is mixing and thus,

70 $$\begin{aligned} \int _{{{\mathcal {M}}}} R_{\rho }^n \phi \cdot \overline{\psi } \, d\mu \rightarrow 0 \text { as } n \rightarrow \infty , \end{aligned}$$

for $\phi , \psi \in {{\mathcal {B}}}$ with $\int _{{{\mathcal {M}}}} \phi \, d\mu = 0$. If $\gcd (r) = d > 1$, then the eigenvalues on the unit circle are the *d*-th roots of unity. Hence,

$$\begin{aligned} R_{{\bar{\Delta }}_\rho } = \Pi _{{\bar{\Delta }}_\rho } + Q_{{\bar{\Delta }}_\rho }:= \sum _{\lambda ^d=1} \lambda \Pi _\lambda + Q_{{\bar{\Delta }}}, \qquad \qquad \Pi _{{\bar{\Delta }}_\rho } Q_{{\bar{\Delta }}_\rho } = Q_{{\bar{\Delta }}_\rho } \Pi _{{\bar{\Delta }}_\rho }= 0, \end{aligned}$$

where $\Pi _\lambda$ denotes the projection on the (generalised) eigenspace ${{\mathcal {B}}}_{{\bar{\Delta }}_\rho ,\lambda }$ of eigenvalue $\lambda$, and $Q_{{\bar{\Delta }}_\rho }$ is the complementary projection. The Banach space ${{\mathcal {B}}}_{{\bar{\Delta }}_{\rho }}$ on ${\bar{\Delta }}_{\rho }$ can be written as the direct sum

71 $$\begin{aligned} {{\mathcal {B}}}_{{\bar{\Delta }}_{\rho }} = {{\mathcal {B}}}_{\Pi } \oplus {{\mathcal {B}}}_{Q} \quad \text { for } \quad {{\mathcal {B}}}_{\Pi }:= \oplus _{\lambda ^d = 1} {{\mathcal {B}}}_{{\bar{\Delta }}_\rho ,\lambda } = \ker (Q_{{\bar{\Delta }}_\rho }) \quad \text { and } \quad {{\mathcal {B}}}_Q = \ker (\Pi _{{\bar{\Delta }}_\rho }),\nonumber \\ \end{aligned}$$

As the kernels of projections, ${{\mathcal {B}}}_{\Pi }$ and ${{\mathcal {B}}}_Q$ are closed $R_{{\bar{\Delta }}_{\rho }}$-invariant subspaces of ${{\mathcal {B}}}_{{\bar{\Delta }}_\rho }$, and hence Banach spaces themselves. Also, as clarified below, for every $\rho >0$, the restriction $R_{{\bar{\Delta }}_{\rho }}$ to ${{\mathcal {B}}}_{Q}$ has spectral radius less than 1. That is, for every $\rho >0$, there exists ${\hat{\theta }}_\rho < 1$ so that

72 $$\begin{aligned} \Vert R^n_{\rho } \phi \Vert _{{{\mathcal {B}}}_{{\bar{\Delta }}_\rho }} \ll {\hat{\theta }}_\rho ^n\,\Vert \phi \Vert _{{{\mathcal {B}}}_{{\bar{\Delta }}_\rho }}. \end{aligned}$$

Consider the lifted version of $\phi$: $\phi _{{\bar{\Delta }}_\rho }(x) = \int _{\ell (x)} \phi \circ \pi \, d\mu _{\Delta _\rho ,\ell (x)}$ where $\ell (x)$ is the stable leaf through $x \in {\bar{\Delta }}$ and $\mu _{\Delta _\rho ,\ell (x)}$ the measure on this leaf emerging from the disintegration of the measure $\mu _{\Delta _\rho }$ of the two-sided tower. The transfer operator $R_{{\bar{\Delta }}_\rho }$ on the one-sided tower satisfies

73 $$\begin{aligned} \int _{{{\mathcal {M}}}} R^n \phi \cdot \overline{\psi } \, d\mu = \int _{{\bar{\Delta }}} R^n_{{\bar{\Delta }}_\rho } \phi _{{\bar{\Delta }}_\rho } \cdot \overline{\psi _{{\bar{\Delta }}_\rho } }\, d\mu _{{\bar{\Delta }}_\rho }. \end{aligned}$$

If $\Pi _{{\bar{\Delta }}_\rho } \phi _{{\bar{\Delta }}_\rho } \ne 0$, then there exists $\psi _{{\bar{\Delta }}_\rho } \in {{\mathcal {B}}}_{{\bar{\Delta }}_\rho }$ such that $\int _{{{\mathcal {M}}}} R^n_{{\bar{\Delta }}_\rho } \phi _{{\bar{\Delta }}_\rho } \cdot \psi _{\Delta _\rho } \, d\mu _{\Delta _\rho } \not \rightarrow 0$. (In fact, taking $\psi _{{\bar{\Delta }}_\rho } = \Pi _{{\bar{\Delta }}_\rho } \phi _{{\bar{\Delta }}_\rho }$, we get $\int _{{{\mathcal {M}}}} R^{dn}_{{\bar{\Delta }}_\rho } \phi _{{\bar{\Delta }}_\rho } \cdot \psi _{{\bar{\Delta }}_\rho } \, d\mu _{{\bar{\Delta }}_\rho } \rightarrow \int _{{{\mathcal {M}}}} \Pi _{{\bar{\Delta }}_\rho } \phi _{{\bar{\Delta }}_\rho } \overline{\Pi _{{\bar{\Delta }}_\rho } \phi _{{\bar{\Delta }}_\rho }} \, d\mu _{{\bar{\Delta }}_\rho } \ne 0$.) This contradicts (70) and/or (73). Hence $\phi _{{\bar{\Delta }}_\rho } \in {{\mathcal {B}}}_{Q}$ and, for the operator norm $\Vert R^n_{{\bar{\Delta }}_\rho } \phi _{{\bar{\Delta }}_\rho } \Vert _{{{\mathcal {B}}}_Q} \le \Vert \, R_{{\bar{\Delta }}_{\rho }}|_{{{\mathcal {B}}}_Q}\, \Vert ^n \, \Vert \phi _{{\bar{\Delta }}_\rho }\Vert _{{{\mathcal {B}}}_Q} \ll {\hat{\theta }}_\rho ^n\, \Vert \phi _{{\bar{\Delta }}_\rho }\Vert _{{{\mathcal {B}}}_Q}$. Property (72) follows.

#### Remark C.5

We note that mixing of the underlying map $T_\rho$ is not required for a useful version of (67) to hold. Indeed the property of $Q_{{\bar{\Delta }}_\rho }$ in (67) holds independently of mixing and for this we just need to work with (72), which holds for $d>1$. The downside of using (72) directly is that in assumption (37) we would have to extract $\sum _{\lambda ^d=1} \lambda \Pi _\lambda (e^{it\kappa _\rho }-1)$ instead of $\int _{{{\mathcal {M}}}} (e^{it\kappa _\rho }-1)\, d\mu$. We found it more convenient to work with the ’clean’ assumption (37).

## Footnotes

1 Actually, [26, Theorem 1.2] is stated for the flight time function taking values in ${{\mathbb {R}}}^2$, as opposed to the displacement function taking values in ${{\mathbb {Z}}}^2$, but these are equivalent as the difference between the two processes is uniformly bounded, see Remark 6.5.

2 We will also use the notations $\gamma _{\rho }=1-\hat{\theta }_{\rho }$ and $\hat{\gamma }_{\rho }=1-\hat{\vartheta }_{\rho }$.

3 Note that our set-up fits the conditions (H1)-(H5) in [16, Sect. 2.1] with $f(x)=f(\theta ,\phi )=\cos \phi$ and $\kappa _\rho =1$ in (H1), $r_h=r_0+1$ in (H2), $\xi =\frac{1}{2}$ and $t_0=1$ in (H3), $p_0=\frac{1}{r_0+1}$ in (H4) and $\gamma _0=0$ in (H5).

4 In the definition of the weak norm [16] uses test functions with $|\psi |_{W,\gamma ,p}\le 1$ for some $\gamma >0$, and requires $p<\gamma$. However, this is needed only to ensure that the inclusion ${{\mathcal {B}}}_w\hookrightarrow (C^p)'$ is injective, cf. [16, Lemma 3.8] Since we do not use this property, we can take $\gamma =0$ in the definition of the weak norm, and avoid additional restrictions on $p_0$.
